# Prevalence and associations of lifetime, 12-month, and 30-day suicidal thoughts and behaviours among 3,702 First-Year university students in New Zealand

**DOI:** 10.1007/s00127-026-03069-5

**Published:** 2026-02-27

**Authors:** A. Mason, T. Winter, S. Fortune, M. Taumoepeau, C. M. Rapsey

**Affiliations:** 1 School of Psychological and Social Sciences, University of Waikato, Hamilton, New Zealand; 2 Mathematics and Statistics, Faculty of Engineering, University of Canterbury, Christchurch, New Zealand; 3 Centre for Alcohol Policy Research, La Trobe University, Melbourne, Australia; 4 School of Population Health, Faculty of Medical and Health Sciences, University of Auckland, Auckland, New Zealand; 5 School of Psychological Sciences, Victoria University of Wellington, Wellington, New Zealand; 6 Department of Psychological Medicine, Dunedin School of Medicine, University of Otago, Dunedin, New Zealand

**Keywords:** Suicide prevention, University students, Suicidal thoughts, Suicide attempt, Psychiatric epidemiology

## Abstract

**Background:**

Reliable epidemiological data about the prevalence, age-of-onset, and correlates of suicide-related outcomes are critical for effective suicide prevention. The present study aimed to investigate the lifetime and past 12-month prevalence of suicidal thoughts, plans, attempts. We then investigated whether these outcomes were associated with socio-demographic variables and mental disorders among first-year university students in New Zealand.

**Methods:**

Data were collected between 2021 and 2023 through online self-report surveys as part of New Zealand’s contribution to the World Mental Health International College Surveys initiative. The final sample consisted of *n =* 3,702 first-year university students (overall response rate: 24.2%). Suicide-related outcomes were assessed using the Columbia-Suicide Severity Rating Scale (C-SSRS). Weighted prevalence estimates were calculated and data were analysed using multivariable statistical methods.

**Results:**

Overall, 54.1% of respondents reported experiencing suicidal thoughts across their lifetime, with 29.6% having made a suicide plan, and 10.1% making at least one suicide attempt. Twelve-month prevalence of these outcomes was 37.4% (thoughts), 29.5% (plan), and 2.9% (attempt), respectively, and 12-month persistence among lifetime cases for these outcomes ranged from two-thirds for ideation to one-quarter for attempts. Non-heterosexual orientation and experiences of Major Depressive Disorder, Bipolar Disorder, or Post-Traumatic Stress Disorder were associated with greater likelihood of suicide-related outcomes.

**Conclusions:**

Suicide-related outcomes are highly prevalent and persistent among first-year university students in New Zealand. Our findings demonstrate that specific demographic characteristics and experiences of mental disorder are associated with greater likelihood of suicide-related outcomes and highlight the need for effective initiatives to support first-year university students.

**Supplementary Information:**

The online version contains supplementary material available at https://doi.org/10.1007/s00127-026-03069-5.

Considerable evidence suggests that suicidal thoughts and behaviours are common among university students [1–4]. Findings from the World Mental Health International College Surveys initiative [5], using data collected between 2014 and 2017 from 13,984 first-year university students across eight countries, estimated lifetime prevalence rates of 32.7% (ideation), 17.5% (plans), and 4.3% (attempts), and 12-month prevalence rates of 17.2% (ideation), 8.8% (plans), and 1.0% (attempts) [2]. A recent update from this initiative using data from 72,288 first-year university students across 19 countries found that these prevalence estimates had increased, with the 12-month prevalence almost doubling (ideation: 17.2% vs. 30.6%; plans: 8.8% vs. 14.0%; attempts: 1.0 vs. 2.3%) [6]. Thus, the first year of university may be a critical point for identification, intervention, and prevention of suicidal thoughts and behaviours [7].

An important component of suicide prevention is determining which factors may increase or decrease an individual’s risk of suicide. Various studies present consistent patterns of associations between suicide-related outcomes and demographic characteristics among university students. For example, older age, female sex, and non-heterosexual sexual orientations are all known correlates of suicide-related outcomes across cross-national surveys and individual studies [8–12]. Course of study does not appear to be associated with differences in suicide-related outcomes or onset [9, 13]. Additionally, a wealth of evidence demonstrates that experiences of common mental disorders, particularly internalising disorders (e.g., Major Depressive Disorder) are associated with greater risk of suicide-related outcomes [14–16]. Longitudinal data also suggests that externalising disorders may confer specific risk for suicide attempt wherein experiences of externalising disorders were associated with suicide attempt but not suicidal ideation alone [17].

Although evidence from other countries and large-scale, cross-national surveys is useful, they overshadow country level variations in the prevalence and correlates for suicide-related outcomes [2, 10, 18]. For example, earlier data from Australia, a comparable context to New Zealand, had elevated rates of suicidal ideation (Lifetime, 44.6%, 12-Month: 25.7%) when compared to the pooled cross-national estimates (Lifetime: 32.7%, 12-Month: 17.2%) [2]. Furthermore, within cross-national data, the presence, magnitude, and direction of associations with correlates were observed to vary by country [2]. Finally, previous studies have found associations between ethnicity and 30-day ideation [8] and, for New Zealand, there is consistent evidence that Māori (the Indigenous peoples of New Zealand) are over-represented among suicide statistics [19]. Together, these findings highlight the need to consider the epidemiology of suicide-related outcomes specifically within a New Zealand context so that we can develop targeted prevention initiatives.

Unfortunately, beyond coronial data about rates of suicide deaths, there is limited evidence about the prevalence and correlates of suicidal thoughts and behaviours in university students in New Zealand. Early evidence from Te Rau Hinengaro, a nationally representative survey of mental disorders in New Zealand, estimated past 12 month prevalence rates of 6.6%, 2.0%, and 1.3% for ideation, plans, and attempts, respectively [20]. However, this data was non-specific to university students and was collected over 20 years ago. Robust epidemiological data documenting suicidal thoughts and behaviours in New Zealand is needed to provide contemporary quantitative evidence about the extent of the issue and provide a benchmark from which the effectiveness of future suicide prevention strategies within university contexts can be measured.

Using data from New Zealand’s WMH-ICS surveys, we aim to present the lifetime and 12-month prevalence of suicidal thoughts, plans, and behaviours from a first-year university population in New Zealand. We will also consider persistence (i.e., 12-month prevalence among lifetime cases) and transition (i.e., suicide attempt among those with suicidal ideation) of these outcomes, age of onset, and the associations between socio-demographics and mental disorder with these outcomes.

## Method

### Participants and procedure

All first-year university students from a New Zealand university across 2021–2023 were invited to complete an online survey via Qualtrics. Invitations to participate were disseminated via email using student email addresses, provided by the host university. These emails contained information about the study and a link to the online survey. The main survey items were prefaced by additional information about the study, and consent and survey display logics were used to ensure consent was obtained before respondents saw the main survey items. Non-respondents were sent up to three additional email invitations. Alongside email distribution, the survey was also promoted at residential colleges.To enhance response rate, all Māori (the indigenous of peoples of New Zealand) and Pacific peoples students, as well as a random sample of non-Māori and non-Pacific peoples students, were informed that they would receive a $50 supermarket voucher for completing the survey. Ethics approval was obtained from the host university Human Ethics Committee (Health), (reference: H21/100).

### Measures

The self-report questionnaire (https://www.hcp.med.harvard.edu/wmh/ftpdir/WMH-ICS_Baseline_survey_V3.2_FINAL_20220228.pdf) was developed in English and has been used consistently throughout studies within the WMH-ICS initiative [6, 21].

### Suicidal thoughts and behaviours

A modified version of the Columbia-Suicide Severity Rating Scale [22] was used to assess suicidal thoughts and behaviours. Suicidal ideation was assessed using the questions “Did you ever wish you were dead or would go to sleep and never wake up?” and “Did you ever in your life have thoughts of killing yourself?”, suicide plans using “Did you ever think about how you might kill yourself (e.g., taking pills, shooting yourself) or work out a plan of how to kill yourself?”, and suicide attempts using “have you ever made a suicide attempt (i.e., purposefully hurt yourself with at least some intent to die)?”. Age of onset, number of lifetime years with suicidal thoughts and behaviours, number of months in the past 12 with suicidal thoughts and behaviours were also assessed. Using these variables, additional composite variables were then derived to consider persistence (i.e., 12-month cases among lifetime) and transition along the suicidal trajectory (e.g., suicide plans given ideation, suicide attempts given plan).

### Mental disorders

The approach to assessment of common mental disorders has been detailed in full elsewhere (e.g., see Mason et al., [21]. Briefly, lifetime experiences of major depressive disorder (MDD), generalized anxiety disorder (GAD) and panic disorder (PD) were assessed via the Composite International Diagnostic Interview Screening Scales, Version 3.2 (CIDI-SC) [23]. Bipolar I/II disorder (BP) and drug use disorder (DUD) were assessed using the Composite International Diagnostic Interview for DSM-5 (CIDI-5) modified for self-report administration. Post-traumatic stress disorder (PTSD) was assessed with the 4-Item Short-Form of the PTSD Checklist for DSM-5 (PCL-5) [24]; attention-deficit/hyperactivity disorder (ADHD) with the Adult Self-Report Scale-V1.1 (ASRS-V1.1) Screener [25]; and alcohol use disorder (AUD) with the Alcohol Use Disorders Identification Test (AUDIT) [26].

For the six mental disorders where lifetime prevalence was assessed, respondents were asked lifetime diagnostic stem questions and then, if affirmative, were asked to focus on the time in their life when the symptoms were most severe. Respondents screening positive for lifetime prevalence were then asked a single question (i.e., rather than repeating all symptom questions) about 12-month prevalence. ADHD and AUD, in comparison, were assessed only for the past 6 months or 12 months, respectively.

### Socio-demographics

The socio-demographics included are respondent age (18–36 + years old, categorised to 18 and under, 19, 20 and older), sex at birth (male, female), gender identity (male, female, another gender), sexual orientation (heterosexual/straight, gay/lesbian, other), parent education (highest education of either parent dichotomized into university degree versus less than university degree), course of study (collapsed in the following groupings: arts and sciences, medical sciences, commerce, laws), and ethnicity (European, Māori, Pacific peoples, Asian, and Middle Eastern Latin American African [MELAA]). Dichotomous variables were created for sexual orientation (heterosexual vs. non-heterosexual), gender modality (cisgender vs. transgender, based on alignment between gender identity and sex at birth), enrolment status (full-time vs. part-time/other). A priority coded variable was used for ethnicity (order of prioritisation: Māori, Pacific peoples, Asian, MELAA, and European).

### Statistical analyses

 Analyses were conducted in R (version 4.5.0). Weights were calculated to match the achieved sample to the first-year university population using iterative proportional fitting (raking) implemented through the *autumn* package in R [27, 28]. Benchmarks for raking were gender (male, female, diverse), age (18, 19, 20–24, and over 24 years old), prioritised ethnicity, and socioeconomic quintile based on the New Zealand Deprivation Index (NZDep) [29]. Additionally, sequential multiple imputation by random forest was used to adjust for within-survey item non-response and random internal subsampling of survey sections [30]. Data were multiply imputed 20 times.

Simple weighted mean calculations were used to estimate lifetime and 12-month prevalence and transition rates of suicide-related outcomes as well as 12-month persistence among lifetime cases. To account for respondents whose age-of-onset for suicidal thoughts and behaviours was in the past 12 months, persistence was defined as respondents who continued to experience the suicide-related outcome in the past 12 months, where age-of-onset had occurred at least two years prior to survey completion.

Multivariable Poisson regression models with robust standard errors, implemented using the *svyglm* package in R [31, 32], were used to determine the associations between sociodemographic correlates and experiences of common mental disorders with suicide-related outcomes [33, 34]. Initially, univariable models including the predictor of interest and adjusting for year of survey and incentivisation were considered. Complete multivariable models including all predictors were then considered. In line with Mortier and colleagues [6], these were built in sequential fashion where sociodemographic characteristics were entered into Block 1, experiences of common mental disorders were added in Block 2, and finally, number of co-occurring mental disorders was added in Block 3. All models were computed using temporally aligned variables (i.e., lifetime suicide-related outcomes with lifetime experiences of common mental disorders and past 12-month suicide-related outcomes with past 12-month experiences of common mental disorders). Exponentiated Poisson regression coefficients are reported as Risk Ratios (RRs) with their 95% confidence intervals; confidence intervals that do not cross through 1.00 are considered to be statistically significant at the *p* < 0.05 level.

## Results

### Sample description

Participant demographics are summarised in Table 1. The final sample consisted of *n* = 3,702 participants, representing an overall response rate of 24.2%. Response rate was observed to fluctuate across year of data collection (2021: 16.1%, 2022: 36.3%, 2023: 22.9) and was higher among individuals who were offered an incentive to participate (40.5% vs. 16.7%). Respondents were aged between 16 and 36 years (Median:19, IQR:18–20), the majority of whom were women (60%; men: 37.4%, gender queer: 2.6%), cisgender (96.6%), heterosexual (73.7%), identified as being of Pākehā (European) descent (60.7%; Māori, 14.4%; Pacific peoples, 4.0%; Asian, 15.9%; Middle Eastern Latin American African [MELAA], 5.0%), and studying fulltime (94.4%; parttime, 2.2%; Non-degree/other, 3.4%).Approximately two-thirds were studying Arts and Science (63.6%; Medical Sciences, 12.1%; Commerce, 19.5%; Laws, 4.8%) and had at least one parent with at least a university degree (63.8%).

**Table 1:** Weighted demographic characteristics of the overall sample and those with or without any lifetime suicidal thoughts or behaviours, *n* = 3,702, % (SE)

	Overall sample (Weighted)	with any Suicidal Thoughts or Behaviours (Weighted)	without any Suicidal Thoughts or Behaviours (Weighted)	Suicidal Thoughts or Behaviours given Demographic Category (Weighted)
**Age (Median, IQR)**	19 (18 - 20)	19 (18 - 20)	19 (18 - 19)	-
**Gender**				
Woman	60.0% (1.1)	64.5% (1.5)	54.6% (1.6)	58.2% (1.2)
Man	37.4% (1.1)	31.1% (1.4)	44.8% (1.6)	45.0% (2.0)
Gender Queer	2.6% (0.3)	4.4% (0.5)	0.6% (0.2)	89.7% (3.0)
**Gender Modality**				
Cisgender	96.6% (0.4)	94.4% (0.6)	99.1% (0.2)	52.9% (1.1)
Transgender	3.4% (0.4)	5.6% (0.6)	0.9% (0.2)	88.7% (2.9)
**Sexuality**				
Heterosexual	73.7% (0.9)	61.6% (1.4)	87.8% (1.0)	45.3% (1.2)
Nonheterosexual	26.3% (0.9)	38.4% (1.4)	12.2% (1.0)	78.8% (1.6)
**Parents Education**				
High School or lower	36.2% (1.0)	36.5% (1.4)	35.8% (1.5)	54.7% (1.8)
University or higher	63.8% (1.0)	63.5% (1.4)	64.2% (1.5)	53.9% (1.3)
**Ethnicity**				
NZ European/Pākeha	60.7% (1.0)	61.8% (1.4)	59.5% (1.5)	-
Māori	14.4% (0.6)	13.3% (0.8)	15.6% (1.0)	50.1% (2.3)
Pacific Peoples	4.0% (0.3)	3.7% (0.4)	4.3% (0.5)	50.2% (4.0)
Asian	15.9% (0.8)	16.3% (1.0)	15.4% (1.2)	55.4% (2.8)
MELAA	5.0% (0.6)	4.9% (0.8)	5.2% (0.9)	52.6% (5.9)
**Course of Study**				
Arts & Science	63.6% (1.0)	61.8% (1.4)	65.7% (1.4)	52.6% (1.3)
Medical Sciences	12.1% (0.7)	9.9% (0.8)	14.7% (1.1)	44.2% (2.9)
Commerce	19.5% (0.8)	22.2% (1.2)	16.4% (1.1)	61.5% (2.2)
Laws	4.8% (0.4)	6.1% (0.7)	3.2% (0.5)	68.9% (4.1)
**Enrolment Status**				
Full time study	94.4% (0.5)	93.7% (0.7)	95.2% (0.7)	53.7% (1.1)
Non full time study	5.6% (0.5)	6.3% (0.7)	4.8% (0.7)	60.8% (4.4)

Gender Queer is defined as any reported gender identity that is not man or woman

Ethnicity was priority coded to Māori, Pacific peoples, Asian, MELAA, Pākehā/European. Māori are the indigenous peoples of Aotearoa New Zealand. MELAA is defined as Middle Eastern, Latin American, African

STB given Demographic Category refers to the proportion of people with any lifetime STB, given the demographic category. For example, if a respondent is a woman, the estimate reflects proportion of woman who have screened positive for a lifetime STB

### Prevalence, persistence, and age-of-onset of suicidal thoughts and behaviours

Estimates are reported in Table 2. The lifetime prevalence for suicidal ideation was 54.1% (SE:1.0), suicide plans, 29.6% (SE:1.0), and suicide attempts 10.1% (SE:0.7), and the 12-month prevalence for these outcomes were ideation, 37.0% (SE:1.1), plans, 18.2% (SE:0.9), and attempts, 2.8% (SE:0.4). Of those reporting lifetime prevalence of suicidal thoughts or plans, the majority continued to experience thoughts (68.3%, SE:1.5) or plans (60.3%, SE:2.2) in the past 12 months. Approximately one-quarter of those who had made at least one lifetime suicide attempt had also attempted in the past 12 months (26.5%, SE:3.6). Median age-of-onset for each of these outcomes was 15 years (IQRs:12–16; Fig. 1).

**Table 2:** Lifetime and 12 month prevalence and persistence of suicidal thoughts and behaviours among 3,702 new Zealand university students, % (SE)

Disorder	Median Age of Onset (IQR)	Lifetime	Past 12 Months	12 Months given Lifetime Cases
1. Suicidal Thoughts or Behaviours				
Suicidal Ideation	15 (12,16)	54.1 (1.0)	37.0 (1.1)	68.0 (1.6)
Suicide Plan	15 (13,16)	29.6 (1.0)	18.2 (0.9)	59.1 (2.4)
Suicide Attempt	15 (13,16)	10.1 (0.7)	2.7 (0.4)	23.4 (3.7)
2. Transition Outcomes given Ideation				
Suicidal Ideation without Plan or Attempt	-	42.8 (1.6)	50.6 (2.0)	-
Suicide Plan given Ideation	-	54.8 (1.6)	49.1 (2.0)	-
Suicide Attempt given Ideation	-	18.7 (1.2)	7.4 (1.1)	-
3. Transition Outcomes if Plan				
Suicide Plan without Attempt	-	70.2 (1.9)	85.5 (2.0)	-
Suicide Attempt given Plan	-	29.8 (1.9)	14.5 (2.0)	-

Abbreviations: SE, standard error, IQR, interquartile range

Data were multiply imputed, m = 20

Age of Onset refers to the age (in years) when symptoms were first experienced

Prevalence estimates are weighted using iterative proportional fitting (raking)

12 Months given Lifetime cases represents the proportion of individuals who continue to experience the suicide-related outcome in the past 12 months (i.e., the proportion for whom the distress persists)

**Fig. 1:**
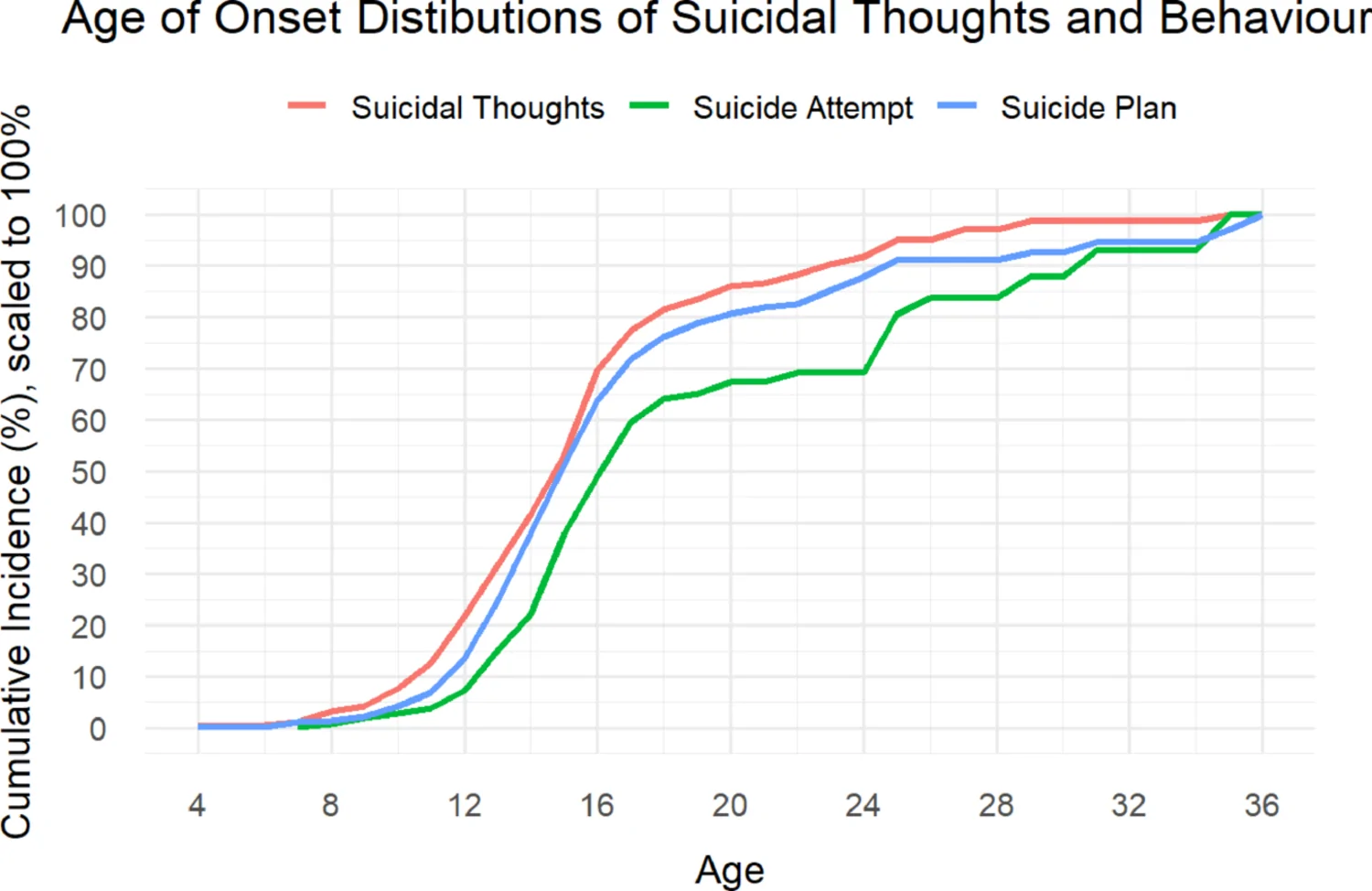
Age of Onset of Suicidal Thoughts and Behaviours

Among those reporting suicidal thoughts, about half of the respondents had made a suicide plan (Lifetime: 54.8%, SE: 1.6; Past 12 Months: 49.1%, SE: 2.0) and a minority had attempted suicide (Lifetime: 18.7%, SE: 1.2; Past 12 Months: 7.4%, SE: 1.1). Among respondents who reported having made a suicide plan, around one-third had also made at least one suicide attempt (Lifetime: 29.8%, SE: 1.9; Past 12 Months: 14.5%, SE: 2.0).

### Associations of socio-demographics variables and mental disorders with lifetime suicidal thoughts and behaviours and progression along the suicidal trajectory

Univariable models (Table 3) show that lifetime experiences of suicidal thoughts and behaviours and progression along the suicidal trajectory were most strongly and generally consistently associated with experiences of co-occurring lifetime mental disorders, and that the size of these associations increased as the number of co-occurring disorders the respondent had experienced also increased (F range 6.58–162.19, *p* < 0.05). These associations were strongest for suicide attempts, particularly for respondents with BP (RR: 5.84, 95% CI: 3.62–9.42) or PTSD (RR: 9.02, 95% CI: 5.82–13.94) who were five and nine times, respectively, more likely to have attempted suicide than their peers without these mental disorders. Among socio-demographic variables, being gender queer, transgender, or having a non-heterosexual orientation were associated with increased risk of suicide-related outcomes. Again, this was most striking for suicide attempts where these respondents were two-to-three times more likely to have attempted suicide than their female (RR: 2.48, 95% CI: 1.48–4.14), cis-gender (RR: 3.21, 95% CI: 1.98–5.20), or heterosexual peers (RR: 2.79, 95% CI: 2.05–3.79). Those who were transgender or non-heterosexual were also more likely to have progressed along the suicidal trajectory if they had thought about suicide, relative to their cisgender (e.g., attempt if ideation, RR: 1.92, 95% CI:1.26–2.92) or heterosexual peers (e.g., attempt if ideation, RR: 1.59, 95% CI:1.22–2.05), though interestingly, were not more likely to have attempted suicide if they have previously made a plan (transgender, RR: 1.46, 95% CI: 0.62–1.68; non-heterosexual, RR: 1.02, 95% CI: 0.79–1.31). Finally, no differences were found for ethnicity on any of the suicide-related outcomes or risk of progressing along the suicidal trajectory.

**Table 3:** The associations of demographics variables and lifetime experiences common mental disorders with lifetime suicide-related outcomes and transition along the suicidal trajectory, RR (95% CI)

	Suicidal Ideation		Suicide Plan		Suicide Attempt	
	Univariable	Multivariable	Univariable	Multivariable	Univariable	Multivariable
**Age (Ref: 18 & Under)**						
19	0.96 (0.87–1.06)	0.93 (0.85–1.02)	0.96 (0.80–1.15)	0.90 (0.77–1.05)	0.95 (0.65–1.38)	0.84 (0.60–1.18)
20 & Older	1.09 (0.99–1.19)	1.01 (0.93–1.10)	1.07 (0.90–1.28)	0.95 (0.81–1.12)	1.34 (0.95–1.88)	1.07 (0.77–1.49)
Age Category: F (df, p)	2.25 (2, *p* = 0.105)	1.45 (2, *p* = 0.234)	0.45 (2, *p* = 0.636)	1.01 (2, *p* = 0.366)	1.65 (2, *p* = 0.194)	0.69 (2, *p* = 0.501)
**Gender (Ref: Female)**						
Male	0.78 (0.71–0.85)*	1.00 (0.92–1.09)	0.73 (0.60–0.88)*	1.08 (0.91–1.27)	0.56 (0.38–0.84)*	0.91 (0.62–1.35)
Gender Queer	1.52 (1.40–1.64)*	1.04 (0.87–1.23)	2.34 (1.80–3.05)*	1.11 (0.78–1.58)	2.48 (1.48–4.14)*	0.75 (0.29–1.94)
Gender: F (df, p)	82.53 (2, *p* < 0.001)	0.10 (2, *p* = 0.902)	37.25 (2, *p* < 0.001)	0.41 (2, *p* = 0.663)	11.61 (2, *p* < 0.001)	0.22 (2, *p* = 0.805)
**Gender Modality (Ref: Cisgender)**						
Transgender	1.65 (1.53–1.78)*	0.96 (0.82–1.12)	2.60 (2.04–3.31)*	1.10 (0.81–1.50)	3.21 (1.98–5.20)*	1.58 (0.66–3.75)
**Sexual Orientation (Ref: Non-heterosexual)**						
Non**-**heterosexual	1.73 (1.62–1.84)*	1.40 (1.31–1.50)*	2.40 (1.98–2.91)*	1.62 (1.41–1.87)*	2.79 (2.05–3.79)*	1.45 (1.10–1.90)*
**Enrolment Status (Ref: Full time)**						
Part-time	1.14 (0.98–1.31)	1.05 (0.92–1.21)	0.96 (0.70–1.32)	0.86 (0.66–1.14)	1.45 (0.85–2.49)	1.11 (0.70–1.77)
**Parental Education (Ref: High School or Less)**						
University or higher	0.98 (0.90–1.06)	1.00 (0.93–1.07)	0.97 (0.83–1.12)	1.01 (0.89–1.16)	0.93 (0.68–1.28)	1.03 (0.76–1.40)
**Ethnicity (Ref: Pākehā/European)**						
Māori	0.98 (0.86–1.10)	0.91 (0.82–1.01)	1.15 (0.92–1.45)	1.01 (0.83–1.24)	1.51 (0.98–2.33)	1.15 (0.78–1.72)
Pacific Peoples	0.98 (0.82–1.17)	1.00 (0.86–1.16)	1.01 (0.70–1.46)	1.03 (0.76–1.39)	1.25 (0.63–2.48)	1.28 (0.72–2.27)
Asian	1.01 (0.91–1.13)	1.17 (1.06–1.30)*	0.90 (0.72–1.12)	1.13 (0.93–1.37)	0.90 (0.57–1.41)	1.27 (0.81–2.00)
MELAA	0.96 (0.77–1.21)	1.02 (0.85–1.21)	1.09 (0.72–1.66)	1.15 (0.82–1.62)	1.17 (0.52–2.66)	1.41 (0.71–2.80)
Ethnicity: F (df, p)	0.09 (4, *p* = 0.986)	3.43 (4, *p* = 0.008)	0.86 (4, *p* = 0.487)	0.50 (4, *p* = 0.739)	1.06 (4, *p* = 0.376)	0.54 (4, *p* = 0.703)
**Course (Ref: Arts & Sciences)**						
Medical Sciences	0.84 (0.73–0.96)*	0.90 (0.80–1.02)	0.71 (0.55–0.92)*	0.77 (0.61–0.98)*	0.67 (0.38–1.16)	0.77 (0.45–1.30)
Commerce	1.16 (1.07–1.27)*	1.10 (1.02–1.19)*	1.27 (1.07–1.52)*	1.15 (0.99–1.34)	1.27 (0.90–1.81)	1.08 (0.78–1.49)
Laws	1.30 (1.14–1.47)*	1.14 (1.00–1.29)	1.46 (1.11–1.91)*	1.19 (0.93–1.53)	1.84 (1.06–3.20)*	1.31 (0.80–2.15)
Course: F (df, p)	12 (3, *p* < 0.001)	4.77 (3, *p* = 0.003)	7.75 (3, *p* < 0.001)	3.65 (3, *p* = 0.012)	3.03 (3, *p* = 0.029)	0.91 (3, *p* = 0.436)
**Experiences of co-occurring Common Mental Disorders**						
MDD	1.82 (1.69–1.96)*	1.21 (1.12–1.31)*	2.54 (1.95–3.30)*	1.45 (1.24–1.70)*	3.11 (1.97–4.92)*	1.80 (1.23–2.64)*
BP	1.72 (1.59–1.87)*	1.27 (1.14–1.41)*	2.56 (1.94–3.39)*	1.61 (1.28–2.02)*	5.84 (3.62–9.42)*	3.07 (1.99–4.73)*
GAD	1.72 (1.61–1.83)*	1.04 (0.97–1.12)	2.53 (2.14–2.98)*	1.16 (0.98–1.37)	3.59 (2.72–4.73)*	1.15 (0.85–1.57)
PD	1.77 (1.64–1.90)*	1.09 (1.00–1.18)	2.84 (2.26–3.57)*	1.36 (1.16–1.60)*	4.10 (2.74–6.13)*	1.33 (0.94–1.89)
PTSD	2.18 (2.00–2.39)*	1.35 (1.21–1.51)*	3.35 (2.76–4.06)*	1.64 (1.33–2.03)*	9.02 (5.83–13.94)*	3.03 (1.77–5.20)*
DUD	1.24 (1.13–1.37)*	0.92 (0.83–1.02)	1.67 (1.38–2.02)*	1.04 (0.86–1.26)	3.05 (2.23–4.19)*	1.31 (0.94–1.83)
AUD	1.05 (0.96–1.14)	0.75 (0.64–0.87)*	1.12 (0.94–1.33)	0.69 (0.53–0.92)*	1.59 (1.15–2.19)*	0.74 (0.43–1.27)
ADHD	1.59 (1.48–1.70)*	0.80 (0.67–0.97)*	2.01 (1.65–2.45)*	0.71 (0.50–1.02)	3.07 (2.18–4.32)*	0.65 (0.32–1.31)
**Number of co-occuring Common Mental Disorders experienced**						
1 Other Disorder	1.80 (1.62–2.00)*	1.54 (1.28–1.85)*	2.29 (1.85–2.83)*	1.61 (1.11–2.32)*	4.43 (2.66–7.39)*	2.20 (0.72–6.69)
2 Other Disorders	2.27 (2.04–2.53)*	2.18 (1.66–2.86)*	3.72 (2.98–4.64)*	2.45 (1.41–4.24)*	10.11 (6.19–16.51)*	4.23 (1.07–16.77)*
3 or more Other Disorders	2.84 (2.57–3.15)*	2.94 (1.95–4.43)*	5.29 (3.97–7.05)*	3.50 (1.52–8.09)*	20.32 (11.55–35.76)*	7.88 (1.29–48.28)*
Number of Disorders experienced: F (df, p)	162.19 (3, *p* < 0.001)	9.53 (3, *p* < 0.001)	55.34 (3, *p* < 0.001)	3.48 (3, *p* = 0.016)	44.26 (3, *p* < 0.001)	2.02 (3, *p* = 0.110)

	Ideation Only		Plan If Ideation		Attempt If Ideation	
	Univariable	Multivariable	Univariable	Multivariable	Univariable	Multivariable
**Age (Ref: 18 & Under)**						
19	1.00 (0.83–1.20)	1.04 (0.88–1.24)	0.99 (0.86–1.15)	0.96 (0.84–1.10)	0.99 (0.69–1.41)	0.89 (0.63–1.24)
20 & Older	1.00 (0.83–1.21)	1.05 (0.87–1.27)	0.98 (0.85–1.14)	0.94 (0.81–1.09)	1.23 (0.89–1.71)	1.06 (0.77–1.47)
Age Category: F (df, p)	0 (2, *p* = 0.998)	0.23 (2, *p* = 0.791)	0.03 (2, *p* = 0.969)	0.45 (2, *p* = 0.640)	0.88 (2, *p* = 0.416)	0.38 (2, *p* = 0.681)
**Gender (Ref: Female)**						
Male	1.09 (0.91–1.31)	0.91 (0.78–1.06)	0.94 (0.80–1.10)	1.09 (0.95–1.27)	0.72 (0.49–1.07)	0.94 (0.65–1.36)
Gender Queer	0.31 (0.13–0.76)*	0.75 (0.20–2.83)	1.52 (1.30–1.79)*	1.11 (0.81–1.52)	1.61 (0.99–2.62)	0.78 (0.30–2.01)
Gender: F (df, p)	4.41 (2, *p* = 0.013)	0.71 (2, *p* = 0.490)	16.85 (2, *p* < 0.001)	0.73 (2, *p* = 0.484)	3.7 (2, *p* = 0.026)	0.15 (2, *p* = 0.862)
**Gender Modality (Ref: Cisgender)**						
Transgender	0.32 (0.16–0.68)*	0.61 (0.20–1.89)	1.56 (1.36–1.78)*	1.10 (0.83–1.47)	1.92 (1.26–2.92)*	1.51 (0.64–3.58)
**Sexual Orientation (Ref: Non-heterosexual)**						
Non**-**heterosexual	0.64 (0.53–0.76)*	0.76 (0.64–0.91)*	1.37 (1.22–1.54)*	1.22 (1.08–1.38)*	1.59 (1.22–2.05)*	1.17 (0.91–1.52)
**Enrolment Status (Ref: Full time)**						
Part-time	1.10 (0.82–1.49)	1.06 (0.81–1.39)	0.84 (0.62–1.13)	0.85 (0.65–1.10)	1.27 (0.77–2.11)	1.13 (0.71–1.78)
**Parental Education (Ref: High School or Less)**						
University or higher	1.01 (0.86–1.18)	1.00 (0.86–1.16)	0.99 (0.87–1.12)	1.00 (0.89–1.13)	0.95 (0.70–1.30)	1.02 (0.76–1.38)
**Ethnicity (Ref: Pākehā/European )**						
Māori	0.77 (0.60–1.00)	0.87 (0.68–1.11)	1.18 (0.98–1.43)	1.08 (0.90–1.29)	1.54 (1.03–2.32)*	1.19 (0.82–1.74)
Pacific Peoples	0.95 (0.67–1.33)	0.92 (0.67–1.27)	1.04 (0.76–1.41)	1.04 (0.79–1.36)	1.28 (0.67–2.44)	1.26 (0.72–2.21)
Asian	1.15 (0.95–1.40)	0.99 (0.82–1.20)	0.88 (0.73–1.06)	1.00 (0.84–1.18)	0.88 (0.57–1.36)	1.21 (0.78–1.87)
MELAA	0.79 (0.45–1.37)	0.85 (0.51–1.41)	1.13 (0.82–1.56)	1.07 (0.80–1.45)	1.21 (0.56–2.63)	1.31 (0.66–2.60)
Ethnicity: F (df, p)	2.14 (4, *p* = 0.074)	0.44 (4, *p* = 0.777)	1.69 (4, *p* = 0.151)	0.25 (4, *p* = 0.908)	1.35 (4, *p* = 0.250)	0.43 (4, *p* = 0.786)
**Course (Ref: Arts & Sciences)**						
Medical Sciences	1.19 (0.97–1.47)	1.19 (0.97–1.47)	0.85 (0.68–1.06)	0.84 (0.68–1.04)	0.80 (0.46–1.37)	0.83 (0.50–1.39)
Commerce	0.86 (0.70–1.06)	0.90 (0.75–1.09)	1.09 (0.95–1.26)	1.07 (0.93–1.22)	1.09 (0.78–1.53)	1.04 (0.76–1.43)
Laws	0.81 (0.55–1.19)	0.89 (0.62–1.28)	1.12 (0.89–1.41)	1.07 (0.86–1.34)	1.41 (0.84–2.38)	1.25 (0.77–2.01)
Course: F (df, p)	2.33 (3, *p* = 0.073)	1.86 (3, *p* = 0.136)	1.72 (3, *p* = 0.162)	1.42 (3, *p* = 0.235)	0.96 (3, *p* = 0.412)	0.53 (3, *p* = 0.659)
**Experiences of co-occurring Common Mental Disorders**						
MDD	0.66 (0.56–0.78)*	0.78 (0.65–0.94)*	1.36 (1.20–1.54)*	1.20 (1.05–1.38)*	1.67 (1.22–2.29)*	1.49 (1.02–2.18)*
BP	0.34 (0.18–0.63)*	0.46 (0.25–0.81)*	1.47 (1.24–1.75)*	1.28 (1.06–1.56)*	3.35 (2.29–4.90)*	2.47 (1.63–3.74)*
GAD	0.55 (0.44–0.70)*	0.84 (0.65–1.08)	1.45 (1.28–1.65)*	1.11 (0.96–1.29)	2.07 (1.56–2.75)*	1.11 (0.82–1.50)
PD	0.43 (0.32–0.59)*	0.61 (0.45–0.83)*	1.58 (1.41–1.78)*	1.25 (1.10–1.43)*	2.28 (1.68–3.12)*	1.23 (0.89–1.70)
PTSD	0.60 (0.50–0.73)*	0.73 (0.59–0.90)*	1.53 (1.27–1.83)*	1.24 (1.04–1.47)*	4.12 (2.68–6.32)*	2.25 (1.37–3.70)*
DUD	0.63 (0.48–0.82)*	0.79 (0.59–1.06)	1.33 (1.17–1.51)*	1.13 (0.96–1.33)	2.43 (1.82–3.24)*	1.38 (1.00–1.90)
AUD	0.88 (0.70–1.09)	0.89 (0.67–1.20)	1.08 (0.94–1.25)	0.90 (0.70–1.17)	1.54 (1.12–2.12)*	0.85 (0.49–1.47)
ADHD	0.70 (0.55–0.88)*	0.90 (0.64–1.27)	1.25 (1.10–1.43)*	0.91 (0.67–1.24)	1.91 (1.42–2.55)*	0.75 (0.37–1.51)
**Number of co-occuring Common Mental Disorders experienced**						
1 Other Disorder	0.79 (0.67–0.93)*	1.16 (0.89–1.51)	1.27 (1.05–1.54)*	1.04 (0.76–1.43)	2.47 (1.49–4.10)*	1.43 (0.48–4.32)
2 Other Disorders	0.54 (0.42–0.68)*	1.29 (0.79–2.10)	1.63 (1.34–1.98)*	1.15 (0.72–1.83)	4.44 (2.73–7.21)*	2.30 (0.59–8.91)
3 or more Other Disorders	0.33 (0.20–0.55)*	1.40 (0.62–3.15)	1.83 (1.48–2.27)*	1.20 (0.59–2.45)	7.03 (4.15–11.93)*	3.43 (0.58–20.32)
Number of Disorders experienced: F (df, p)	13.51 (3, *p* < 0.001)	0.36 (3, *p* = 0.785)	14.48 (3, *p* < 0.001)	0.19 (3, *p* = 0.906)	21.92 (3, *p* < 0.001)	0.87 (3, *p* = 0.458)

		Plan No Attempt		Attempt If Plan		
		Univariable	Multivariable	Univariable	Multivariable	
**Age (Ref: 18 & Under)**						
19		1.01 (0.88–1.17)	1.04 (0.92–1.19)	0.96 (0.65–1.40)	0.90 (0.62–1.28)	
20 & Older		0.90 (0.76–1.07)	0.94 (0.80–1.11)	1.24 (0.90–1.72)	1.13 (0.81–1.59)	
Age Category: F (df, p)		0.78 (2, *p* = 0.460)	0.53 (2, *p* = 0.592)	0.94 (2, *p* = 0.391)	0.53 (2, *p* = 0.589)	
**Gender (Ref: Female)**						
Male		1.10 (0.96–1.27)	1.05 (0.92–1.20)	0.77 (0.51–1.16)	0.85 (0.57–1.26)	
Gender Queer		0.93 (0.71–1.23)	1.22 (0.59–2.51)	1.13 (0.67–1.89)	0.70 (0.27–1.81)	
Gender: F (df, p)		1.19 (2, *p* = 0.307)	0.34 (2, *p* = 0.712)	0.97 (2, *p* = 0.380)	0.48 (2, *p* = 0.618)	
**Gender Modality (Ref: Cisgender)**						
Transgender		0.86 (0.66–1.11)	0.79 (0.40–1.58)	1.34 (0.88–2.06)	1.46 (0.62–3.43)	
**Sexual Orientation (Ref: Non-heterosexual)**						
Non**-**heterosexual		0.92 (0.83–1.03)	0.99 (0.89–1.11)	1.21 (0.94–1.57)	1.02 (0.79–1.31)	
**Enrolment Status (Ref: Full time)**						
Part-time		0.90 (0.67–1.21)	0.97 (0.73–1.28)	1.23 (0.72–2.10)	1.02 (0.62–1.68)	
**Parental Education (Ref: High School or Less)**						
University or higher		1.03 (0.90–1.17)	0.98 (0.87–1.11)	0.94 (0.69–1.27)	1.03 (0.77–1.38)	
**Ethnicity (Ref: Pākehā/European )**						
Māori		0.89 (0.73–1.09)	0.96 (0.80–1.17)	1.28 (0.82–1.98)	1.09 (0.74–1.60)	
Pacific Peoples		0.90 (0.65–1.26)	0.90 (0.67–1.21)	1.23 (0.63–2.40)	1.18 (0.65–2.13)	
Asian		1.00 (0.85–1.18)	0.93 (0.78–1.10)	0.99 (0.65–1.50)	1.26 (0.83–1.94)	
MELAA		0.98 (0.70–1.38)	0.95 (0.70–1.27)	1.02 (0.44–2.35)	1.19 (0.59–2.41)	
Ethnicity: F (df, p)		0.36 (4, *p* = 0.834)	0.28 (4, *p* = 0.888)	0.37 (4, *p* = 0.834)	0.37 (4, *p* = 0.833)	
**Course (Ref: Arts & Sciences)**						
Medical Sciences		1.03 (0.84–1.26)	1.00 (0.82–1.22)	0.93 (0.56–1.55)	0.96 (0.58–1.57)	
Commerce		1.02 (0.89–1.17)	0.98 (0.86–1.12)	0.96 (0.68–1.35)	1.02 (0.74–1.40)	
Laws		0.90 (0.67–1.22)	0.94 (0.73–1.23)	1.21 (0.71–2.07)	1.11 (0.68–1.84)	
Course: F (df, p)		0.22 (3, *p* = 0.884)	0.09 (3, *p* = 0.966)	0.26 (3, *p* = 0.855)	0.09 (3, *p* = 0.967)	
**Experiences of co-occurring Common Mental Disorders**						
MDD		0.90 (0.79–1.02)	0.93 (0.79–1.09)	1.30 (0.93–1.83)	1.27 (0.86–1.87)	
BP		0.54 (0.32–0.92)*	0.63 (0.44–0.90)*	2.26 (1.57–3.24)*	1.89 (1.23–2.88)*	
GAD		0.82 (0.72–0.94)*	0.98 (0.83–1.14)	1.56 (1.17–2.06)*	1.03 (0.75–1.43)	
PD		0.80 (0.69–0.94)*	0.96 (0.81–1.13)	1.60 (1.16–2.19)*	1.06 (0.78–1.45)	
PTSD		0.72 (0.65–0.80)*	0.82 (0.71–0.96)*	3.19 (1.90–5.34)*	1.98 (1.14–3.45)*	
DUD		0.66 (0.53–0.81)*	0.78 (0.63–0.96)*	2.05 (1.56–2.69)*	1.35 (0.98–1.86)	
AUD		0.86 (0.76–0.99)*	0.99 (0.76–1.27)	1.42 (1.03–1.95)*	0.82 (0.48–1.40)	
ADHD		0.79 (0.67–0.93)*	1.01 (0.76–1.33)	1.60 (1.21–2.13)*	0.74 (0.38–1.45)	
**Number of co-occuring Common Mental Disorders experienced**						
1 Other Disorder		0.84 (0.75–0.94)*	1.05 (0.86–1.30)	2.15 (1.22–3.81)*	1.40 (0.40–4.88)	
2 Other Disorders		0.69 (0.60–0.80)*	0.98 (0.64–1.49)	3.29 (1.93–5.60)*	2.35 (0.54–10.22)	
3 or more Other Disorders		0.55 (0.41–0.73)*	0.86 (0.44–1.69)	4.35 (2.50–7.58)*	3.47 (0.56–21.52)	
Number of Disorders experienced: F (df, p)		11.97 (3, *p* < 0.001)	0.71 (3, *p* = 0.544)	10.44 (3, *p* < 0.001)	0.96 (3, *p* = 0.412)	

Abbreviations: RR, Risk Ratio, 95% CI, 95% Confidence Interval

Risk ratios and results are from multivariable Poisson distribution and robust standard errors. Data was multiply imputed, m = 20

All univariable models controlled for year of survey and incentivisation. All multivariable models controlled for all variables included in the model, and for year of survey and incentivisation

* denotes significance difference, *p* < 0.05

The fully adjusted multivariable models showed marked attenuation of independent associations as compared to the univariable associations. Notably, the associations for mental disorders were generally observed to approximately halve in magnitude and the majority of the associations with socio-demographic variables were no longer significant. Sequential multivariable modelling (shown in Supplementary Table A) reveals that these changes were largely explained by the mutual adjustment of mental disorder and to a lesser extent, the mutual adjustment of socio-demographic variables.

Within the fully adjusted models, non-heterosexual orientation was consistently associated with greater risk of lifetime suicide-related outcomes. Most strikingly, compared to their heterosexual peers, non-heterosexual students were approximately 62% more at risk of suicide plan (RR: 1.62, 95% CI: 1.41–1.87) and 45% more likely to attempt suicide (RR: 1.45, 95% CI:1.10–1.90). Additionally, non-heterosexual respondents who were already thinking about suicide were about 24% less likely to *only* experience suicidal thoughts (RR: 0.76, 95% CI: 0.64–0.91) and about 20% more likely to make a suicide plan than their heterosexual peers (RR: 1.22, 95% CI: 1.08–1.38). Modest increases in risk for suicidal ideation were also observed for students who were of Asian ethnicity (RR:1.17, 95% CI: 1.06–1.30) or studying commerce (RR:1.10, 95% CI: 1.02–1.19), and students studying medical sciences had lower risk of making a suicide plan (RR:0.77, 95% CI:0.61–0.98). No associations were found between age, gender, gender modality, enrolment status and parental education and lifetime suicide-related outcomes or progression along the suicidal trajectory.

Among respondents who had experienced a mental disorder, those with BP or PTSD were most at risk of having made a suicide attempt (BP, RR: 3.07, [95% CI:1.99–4.73]; PTSD, RR: 3.03, [95% CI: 1.77–5.20]) or progressing along the suicidal trajectory to attempt (e.g., attempt if plan, BP, RR: 1.89, [95% CI:1.23–2.88]; PTSD, RR: 1.98, [95% CI: 1.14–3.45]). MDD was also consistently, albeit more modestly, associated with increased risk of suicide-related outcomes (e.g., suicide attempt, RR: 1.80 [95% CI: 1.23–2.64]) and progression if the individual had experienced suicidal thoughts (e.g., attempt if ideation, RR: 1.49 [95% CI: 1.02–2.18]) yet lower risk of experiencing suicidal thoughts alone (RR: 0.78, 95% CI:0.65–0.94). Interestingly, the association between ADHD and suicidal ideation flipped between the univariable and fully adjusted multivariable models such that, in the adjusted model, ADHD conferred less risk of suicidal thoughts (RR: 1.59, [95% CI:1.48–1.70] vs. RR: 0.80, [95% CI: 0.67–0.97]). AUD was associated with reduced risk of suicidal ideation (RR: 0.75, 95% CI: 0.64–0.87) and plans (RR: 0.69, 95% CI: 0.53–0.92) within the multivariable models but was not associated with reduced risk within the univariable models (ideation, RR: 1.05 [95% CI:0.96–1.14], plan, RR: 1.12 [95% CI: 0.94–1.33]).

### Associations of socio-demographics variables and mental disorders with 12-month suicidal thoughts and behaviours and progression along the suicidal trajectory

The pattern of associations for past 12-month experiences of suicidal thoughts and behaviours and progression along the suicidal trajectory (shown in Table 4, with sequential models shown in Supplementary Table B) were largely consistent with those observed for lifetime experiences of these outcomes. For this reason, we have only commented on the substantive differences (Table 4).

**Table 4:** The associations of demographics variables and past 12-month experiences of common mental disorders with past 12 month suicide-related outcomes and transition along the suicidal trajectory, RR (95% CI)

	Suicidal Ideation		Suicide Plan		Suicide Attempt	
	Univariable	Multivariable	Univariable	Multivariable	Univariable	Multivariable
**Age (Ref: 18 & Under)**						
19	0.93 (0.80 - 1.08)	0.91 (0.79 - 1.04)	0.96 (0.73 - 1.25)	0.90 (0.72 - 1.14)	0.71 (0.29 - 1.79)	0.67 (0.29 - 1.53)
20 & Older	0.93 (0.81 - 1.08)	0.89 (0.78 - 1.03)	0.97 (0.75 - 1.25)	0.87 (0.69 - 1.10)	1.00 (0.50 - 1.98)	0.92 (0.46 - 1.83)
Age Category: F (df, p)	0.77 (2, p = 0.465)	1.91 (2, p = 0.148)	0.08 (2, p = 0.928)	0.90 (2, p = 0.408)	0.29 (2, p = 0.750)	0.47 (2, p = 0.626)
**Gender (Ref: Female)**						
Male	0.73 (0.64 - 0.84)*	1.01 (0.88 - 1.15)	0.67 (0.51 - 0.87)*	1.05 (0.82 - 1.35)	0.49 (0.22 - 1.09)	0.89 (0.40 - 1.96)
Gender Queer	1.85 (1.55 - 2.20)*	0.99 (0.72 - 1.37)	2.59 (1.88 - 3.58)*	0.87 (0.54 - 1.40)	3.30 (1.43 - 7.62)*	0.64 (0.11 - 3.73)
Gender: F (df, p)	41.5 (2, p < 0.001)	0.01 (2, p = 0.992)	25.17 (2, p < 0.001)	0.36 (2, p = 0.695)	6.24 (2, p = 0.002)	0.13 (2, p = 0.877)
**Gender Modality (Ref: Cisgender)**						
Transgender	2.08 (1.78 - 2.42)*	1.09 (0.83 - 1.44)	3.18 (2.35 - 4.29)*	1.45 (0.95 - 2.20)	4.46 (2.14 - 9.26)*	2.07 (0.46 - 9.34)
**Sexual Orientation (Ref: Non-heterosexual)**						
Non-heterosexual	2.08 (1.86 - 2.32)*	1.50 (1.35 - 1.67)*	2.87 (2.31 - 3.58)*	1.72 (1.40 - 2.11)*	4.28 (2.33 - 7.88)*	1.74 (0.96 - 3.15)
**Enrolment Status (Ref: Full time)**						
Part-time	0.97 (0.75 - 1.25)	0.97 (0.76 - 1.23)	0.95 (0.60 - 1.50)	0.92 (0.61 - 1.37)	1.74 (0.61 - 4.95)	1.31 (0.49 - 3.50)
**Parental Education (Ref: High School or Less)**						
University or higher	0.98 (0.87 - 1.11)	0.99 (0.89 - 1.11)	0.99 (0.80 - 1.22)	1.02 (0.85 - 1.23)	0.77 (0.44 - 1.34)	0.86 (0.48 - 1.53)
**Ethnicity (Ref: Pākehā/European )**						
Māori	1.05 (0.87 - 1.25)	0.92 (0.79 - 1.08)	1.33 (0.97 - 1.82)	1.07 (0.82 - 1.40)	1.93 (0.84 - 4.44)	1.15 (0.53 - 2.47)
Pacific Peoples	1.03 (0.80 - 1.34)	1.02 (0.82 - 1.28)	1.12 (0.68 - 1.85)	1.08 (0.72 - 1.61)	1.73 (0.44 - 6.75)	1.65 (0.51 - 5.30)
Asian	0.97 (0.83 - 1.15)	1.18 (1.00 - 1.38)	0.92 (0.69 - 1.23)	1.16 (0.89 - 1.51)	0.97 (0.44 - 2.18)	1.62 (0.73 - 3.59)
MELAA	1.00 (0.73 - 1.38)	1.01 (0.78 - 1.30)	1.29 (0.73 - 2.28)	1.21 (0.74 - 1.97)	0.40 (0.00 - 320.22)	1.08 (0.16 - 7.35)
Ethnicity: F (df, p)	0.12 (4, p = 0.976)	1.53 (4, p = 0.190)	1.16 (4, p = 0.327)	0.42 (4, p = 0.793)	0.22 (4, p = 0.927)	0.43 (4, p = 0.791)
**Course (Ref: Arts & Sciences)**						
Medical Sciences	0.78 (0.64 - 0.96)*	0.83 (0.69 - 0.99)*	0.70 (0.49 - 1.01)	0.75 (0.54 - 1.04)	0.58 (0.21 - 1.57)	0.64 (0.26 - 1.59)
Commerce	1.21 (1.06 - 1.38)*	1.11 (0.98 - 1.26)	1.39 (1.08 - 1.78)*	1.20 (0.98 - 1.48)	1.11 (0.54 - 2.31)	0.92 (0.47 - 1.80)
Laws	1.34 (1.09 - 1.66)*	1.13 (0.92 - 1.39)	1.60 (1.10 - 2.32)*	1.27 (0.91 - 1.76)	2.56 (0.98 - 6.73)	1.50 (0.56 - 3.98)
Course: F (df, p)	7.43 (3, p < 0.001)	3.38 (3, p = 0.018)	6.06 (3, p = 0.000)	3.09 (3, p = 0.026)	1.85 (3, p = 0.138)	0.58 (3, p = 0.630)
**Experiences of co-occuring Common Mental Disorders**						
MDD	2.44 (2.15 - 2.77)*	1.41 (1.21 - 1.64)*	3.62 (2.74 - 4.80)*	1.87 (1.36 - 2.56)*	3.85 (1.84 - 8.08)*	3.65 (1.37 - 9.73)*
BP	2.30 (1.94 - 2.72)*	1.50 (1.23 - 1.83)*	3.60 (2.53 - 5.12)*	2.24 (1.53 - 3.29)*	10.81 (5.39 - 21.66)*	7.34 (2.20 - 24.45)*
GAD	2.33 (2.10 - 2.59)*	1.13 (1.01 - 1.26)*	3.61 (2.99 - 4.35)*	1.31 (1.06 - 1.62)*	6.12 (3.34 - 11.23)*	1.19 (0.63 - 2.27)
PD	2.37 (2.10 - 2.69)*	1.13 (1.00 - 1.28)	3.64 (2.90 - 4.57)*	1.29 (1.01 - 1.65)*	7.62 (3.87 - 15.02)*	1.67 (0.81 - 3.45)
PTSD	2.59 (2.26 - 2.98)*	1.29 (1.13 - 1.47)*	3.72 (2.88 - 4.80)*	1.34 (1.04 - 1.71)*	9.97 (4.64 - 21.45)*	2.07 (0.94 - 4.57)
DUD	1.53 (1.32 - 1.77)*	1.03 (0.88 - 1.21)	2.05 (1.60 - 2.63)*	1.12 (0.87 - 1.46)	5.84 (3.30 - 10.33)*	1.64 (0.85 - 3.16)
AUD	1.13 (1.00 - 1.28)	0.69 (0.54 - 0.88)*	1.10 (0.88 - 1.38)	0.52 (0.34 - 0.80)*	2.74 (1.31 - 5.74)*	0.84 (0.23 - 3.07)
ADHD	1.93 (1.71 - 2.18)*	0.78 (0.59 - 1.04)	2.48 (1.97 - 3.12)*	0.60 (0.35 - 1.00)	4.72 (2.67 - 8.36)*	0.57 (0.11 - 2.95)
**Number of co-occuring Common Mental Disorders experienced**						
1 Other Disorder	2.20 (1.87 - 2.60)*	1.77 (1.30 - 2.41)*	2.81 (2.01 - 3.92)*	2.02 (1.20 - 3.41)*	5.22 (1.64 - 16.59)*	2.81 (0.00 - 3805.62)
2 Other Disorders	3.29 (2.77 - 3.91)*	2.73 (1.72 - 4.33)*	5.91 (4.42 - 7.91)*	4.05 (1.86 - 8.83)*	22.40 (7.54 - 66.60)*	7.96 (0.01 - 12008.81)
3 or more Other Disorders	4.12 (3.39 - 5.00)*	3.58 (1.81 - 7.08)*	8.06 (5.67 - 11.44)*	6.37 (1.88 - 21.59)*	41.93 (12.58 - 139.79)*	15.75 (0.01 - 38949.98)
Number of Disorders experienced: F (df, p)	91.79 (3, p < 0.001)	7.39 (3, p < 0.001)	53.3 (3, p < 0.001)	4.72 (3, p = 0.003)	19.9 (3, p < 0.001)	0.20 (3, p = 0.893)

	Ideation Only		Plan If Ideation		Attempt If Ideation	
	Univariable	Multivariable	Univariable	Multivariable	Univariable	Multivariable
**Age (Ref: 18 & Under)**						
19	0.97 (0.77 - 1.21)	1.02 (0.84 - 1.24)	1.03 (0.82 - 1.29)	0.98 (0.80 - 1.20)	0.76 (0.31 - 1.85)	0.71 (0.32 - 1.57)
20 & Older	0.97 (0.79 - 1.18)	1.01 (0.83 - 1.23)	1.03 (0.84 - 1.27)	0.98 (0.80 - 1.19)	1.07 (0.54 - 2.13)	1.01 (0.52 - 1.96)
Age Category: F (df, p)	0.08 (2, p = 0.926)	0.03 (2, p = 0.968)	0.06 (2, p = 0.940)	0.04 (2, p = 0.958)	0.24 (2, p = 0.787)	0.39 (2, p = 0.676)
**Gender (Ref: Female)**						
Male	1.10 (0.90 - 1.34)	0.97 (0.81 - 1.16)	0.90 (0.72 - 1.13)	1.04 (0.83 - 1.30)	0.65 (0.29 - 1.47)	0.90 (0.41 - 1.96)
Gender Queer	0.61 (0.36 - 1.03)	1.59 (0.48 - 5.24)	1.39 (1.08 - 1.78)*	0.89 (0.61 - 1.32)	1.78 (0.77 - 4.11)	0.61 (0.10 - 3.59)
Gender: F (df, p)	2.29 (2, p = 0.103)	0.34 (2, p = 0.709)	3.98 (2, p = 0.020)	0.36 (2, p = 0.698)	1.74 (2, p = 0.178)	0.15 (2, p = 0.857)
**Gender Modality (Ref: Cisgender)**						
Transgender	0.53 (0.32 - 0.86)*	0.46 (0.15 - 1.43)	1.51 (1.23 - 1.85)*	1.31 (0.94 - 1.84)	2.14 (1.07 - 4.28)*	2.02 (0.47 - 8.69)
**Sexual Orientation (Ref: Non-heterosexual)**						
Non-heterosexual	0.72 (0.60 - 0.87)*	0.84 (0.70 - 1.00)	1.37 (1.16 - 1.63)*	1.21 (1.02 - 1.43)*	2.07 (1.17 - 3.66)*	1.38 (0.79 - 2.41)
**Enrolment Status (Ref: Full time)**						
Part-time	1.01 (0.70 - 1.46)	1.01 (0.71 - 1.43)	0.98 (0.65 - 1.46)	0.96 (0.67 - 1.37)	1.80 (0.66 - 4.89)	1.36 (0.54 - 3.47)
**Parental Education (Ref: High School or Less)**						
University or higher	0.99 (0.84 - 1.18)	0.99 (0.85 - 1.15)	1.01 (0.85 - 1.20)	1.02 (0.87 - 1.19)	0.79 (0.46 - 1.36)	0.87 (0.50 - 1.49)
**Ethnicity (Ref: Pākehā/European )**						
Māori	0.79 (0.61 - 1.02)	0.83 (0.66 - 1.05)	1.27 (0.98 - 1.63)	1.17 (0.94 - 1.46)	1.81 (0.82 - 4.01)	1.16 (0.55 - 2.44)
Pacific Peoples	0.92 (0.65 - 1.30)	0.92 (0.67 - 1.26)	1.09 (0.73 - 1.63)	1.09 (0.77 - 1.54)	1.68 (0.45 - 6.29)	1.82 (0.63 - 5.26)
Asian	1.05 (0.85 - 1.30)	0.96 (0.77 - 1.20)	0.95 (0.74 - 1.21)	1.04 (0.81 - 1.33)	0.99 (0.45 - 2.18)	1.57 (0.71 - 3.44)
MELAA	0.74 (0.40 - 1.36)	0.81 (0.44 - 1.49)	1.26 (0.83 - 1.91)	1.13 (0.74 - 1.70)	0.37 (0.00 - 397.11)	1.01 (0.15 - 6.71)
Ethnicity: F (df, p)	1.23 (4, p = 0.297)	0.61 (4, p = 0.654)	1.27 (4, p = 0.279)	0.51 (4, p = 0.729)	0.18 (4, p = 0.946)	0.54 (4, p = 0.704)
**Course (Ref: Arts & Sciences)**						
Medical Sciences	1.10 (0.86 - 1.41)	1.10 (0.85 - 1.43)	0.89 (0.64 - 1.23)	0.88 (0.64 - 1.20)	0.72 (0.27 - 1.90)	0.73 (0.30 - 1.81)
Commerce	0.86 (0.68 - 1.09)	0.87 (0.71 - 1.08)	1.16 (0.94 - 1.42)	1.13 (0.94 - 1.36)	0.94 (0.46 - 1.94)	0.96 (0.50 - 1.85)
Laws	0.81 (0.52 - 1.25)	0.83 (0.58 - 1.20)	1.21 (0.87 - 1.67)	1.19 (0.88 - 1.60)	1.94 (0.73 - 5.18)	1.54 (0.57 - 4.12)
Course: F (df, p)	1.19 (3, p = 0.314)	1.19 (3, p = 0.315)	1.33 (3, p = 0.263)	1.26 (3, p = 0.286)	0.9 (3, p = 0.441)	0.44 (3, p = 0.724)
**Experiences of co-occurring Common Mental Disorders**						
MDD	0.69 (0.58 - 0.82)*	0.79 (0.62 - 1.00)	1.47 (1.23 - 1.74)*	1.32 (1.00 - 1.73)	1.55 (0.85 - 2.85)	2.51 (0.98 - 6.46)
BP	0.50 (0.28 - 0.89)*	0.57 (0.32 - 1.02)	1.54 (1.20 - 1.97)*	1.47 (1.06 - 2.04)*	4.62 (2.07 - 10.30)*	4.70 (1.51 - 14.66)*
GAD	0.61 (0.49 - 0.77)*	0.83 (0.66 - 1.05)	1.54 (1.29 - 1.85)*	1.16 (0.97 - 1.39)	2.69 (1.38 - 5.22)*	1.14 (0.62 - 2.10)
PD	0.60 (0.43 - 0.82)*	0.79 (0.61 - 1.04)	1.52 (1.18 - 1.94)*	1.15 (0.95 - 1.38)	3.24 (1.64 - 6.42)*	1.51 (0.74 - 3.09)
PTSD	0.71 (0.55 - 0.91)*	0.92 (0.72 - 1.17)	1.43 (1.08 - 1.90)*	1.07 (0.88 - 1.31)	3.87 (1.82 - 8.19)*	1.72 (0.81 - 3.68)
DUD	0.70 (0.52 - 0.95)*	0.82 (0.61 - 1.09)	1.32 (1.09 - 1.61)*	1.13 (0.90 - 1.41)	3.79 (2.18 - 6.60)*	1.63 (0.86 - 3.11)
AUD	1.02 (0.85 - 1.23)	1.16 (0.84 - 1.60)	0.97 (0.81 - 1.17)	0.71 (0.50 - 1.02)	2.42 (1.15 - 5.09)*	0.98 (0.26 - 3.72)
ADHD	0.75 (0.60 - 0.95)*	0.99 (0.66 - 1.50)	1.27 (1.06 - 1.53)*	0.79 (0.51 - 1.21)	2.45 (1.44 - 4.18)*	0.66 (0.12 - 3.55)
**Number of co-occuring Common Mental Disorders experienced**						
1 Other Disorder	0.85 (0.69 - 1.05)	1.00 (0.72 - 1.38)	1.27 (0.93 - 1.75)	1.12 (0.72 - 1.74)	2.41 (0.75 - 7.68)	1.50 (0.00 - 1812.17)
2 Other Disorders	0.56 (0.43 - 0.73)*	0.90 (0.51 - 1.61)	1.79 (1.35 - 2.38)*	1.53 (0.80 - 2.93)	6.80 (2.25 - 20.48)*	3.37 (0.00 - 4159.24)
3 or more Other Disorders	0.48 (0.29 - 0.77)*	0.96 (0.35 - 2.60)	1.94 (1.37 - 2.73)*	1.81 (0.67 - 4.90)	10.19 (2.93 - 35.49)*	5.73 (0.00 - 10962.60)
Number of Disorders experienced: F (df, p)	7.75 (3, p < 0.001)	0.22 (3, p = 0.885)	9.12 (3, p < 0.001)	0.90 (3, p = 0.440)	8.42 (3, p < 0.001)	0.09 (3, p = 0.968)

	Plan No Attempt			Attempt If Plan		
	Univariable	Multivariable		Univariable	Multivariable	
**Age (Ref: 18 & Under)**						
19	1.04 (0.93 - 1.17)	1.05 (0.94 - 1.17)		0.74 (0.30 - 1.81)	0.69 (0.31 - 1.52)	
20 & Older	0.99 (0.87 - 1.13)	1.00 (0.88 - 1.12)		1.03 (0.52 - 2.05)	1.02 (0.53 - 1.95)	
Age Category: F (df, p)	0.28 (2, p = 0.752)	0.45 (2, p = 0.635)		0.26 (2, p = 0.768)	0.46 (2, p = 0.633)	
**Gender (Ref: Female)**						
Male	1.05 (0.93 - 1.17)	1.01 (0.90 - 1.13)		0.73 (0.33 - 1.64)	0.88 (0.42 - 1.86)	
Gender Queer	0.94 (0.77 - 1.15)	1.00 (0.66 - 1.51)		1.30 (0.56 - 2.99)	0.77 (0.13 - 4.50)	
Gender: F (df, p)	0.58 (2, p = 0.559)	0.01 (2, p = 0.993)		0.58 (2, p = 0.560)	0.08 (2, p = 0.923)	
**Gender Modality (Ref: Cisgender)**						
Transgender	0.93 (0.79 - 1.09)	0.96 (0.69 - 1.34)		1.44 (0.72 - 2.88)	1.48 (0.35 - 6.28)	
**Sexual Orientation (Ref: Non-heterosexual)**						
Non-heterosexual	0.94 (0.85 - 1.03)	0.97 (0.89 - 1.06)		1.49 (0.84 - 2.63)	1.14 (0.66 - 1.95)	
**Enrolment Status (Ref: Full time)**						
Part-time	0.86 (0.64 - 1.17)	0.88 (0.68 - 1.15)		1.81 (0.73 - 4.51)	1.43 (0.62 - 3.32)	
**Parental Education (Ref: High School or Less)**						
University or higher	1.04 (0.95 - 1.15)	1.03 (0.93 - 1.13)		0.79 (0.47 - 1.32)	0.84 (0.50 - 1.43)	
**Ethnicity (Ref: Pākehā/European )**						
Māori	0.94 (0.82 - 1.08)	0.99 (0.86 - 1.13)		1.41 (0.62 - 3.18)	1.10 (0.52 - 2.32)	
Pacific Peoples	0.92 (0.70 - 1.23)	0.91 (0.72 - 1.16)		1.47 (0.36 - 5.99)	1.44 (0.47 - 4.39)	
Asian	0.99 (0.87 - 1.12)	0.93 (0.82 - 1.05)		1.08 (0.51 - 2.26)	1.54 (0.75 - 3.16)	
MELAA	1.04 (0.82 - 1.32)	0.97 (0.76 - 1.25)		0.30 (0.00 - 405.27)	0.99 (0.15 - 6.30)	
Ethnicity: F (df, p)	0.26 (4, p = 0.902)	0.39 (4, p = 0.815)		0.15 (4, p = 0.964)	0.37 (4, p = 0.827)	
**Course (Ref: Arts & Sciences)**						
Medical Sciences	1.02 (0.88 - 1.20)	1.01 (0.87 - 1.17)		0.84 (0.31 - 2.32)	0.83 (0.32 - 2.11)	
Commerce	1.03 (0.92 - 1.14)	1.00 (0.90 - 1.11)		0.84 (0.41 - 1.72)	0.88 (0.48 - 1.63)	
Laws	0.88 (0.67 - 1.15)	0.91 (0.70 - 1.19)		1.67 (0.67 - 4.17)	1.31 (0.49 - 3.49)	
Course: F (df, p)	0.4 (3, p = 0.750)	0.17 (3, p = 0.919)		0.63 (3, p = 0.596)	0.24 (3, p = 0.866)	
**Experiences of co-occuring Common Mental Disorders**						
MDD	0.99 (0.90 - 1.09)	0.93 (0.84 - 1.02)		1.07 (0.57 - 2.01)	2.04 (0.81 - 5.13)	
BP	0.74 (0.56 - 0.97)*	0.75 (0.59 - 0.95)*		3.02 (1.41 - 6.47)*	3.63 (1.24 - 10.65)*	
GAD	0.91 (0.82 - 1.02)	1.00 (0.90 - 1.11)		1.71 (0.92 - 3.20)	0.94 (0.51 - 1.73)	
PD	0.87 (0.77 - 1.00)	0.95 (0.83 - 1.08)		2.08 (1.06 - 4.08)*	1.32 (0.68 - 2.58)	
PTSD	0.87 (0.79 - 0.96)*	0.94 (0.85 - 1.04)		2.69 (1.25 - 5.80)*	1.53 (0.71 - 3.34)	
DUD	0.77 (0.63 - 0.94)*	0.86 (0.71 - 1.03)		2.90 (1.70 - 4.95)*	1.49 (0.81 - 2.74)	
AUD	0.87 (0.78 - 0.96)*	0.91 (0.75 - 1.11)		2.46 (1.18 - 5.12)*	1.16 (0.30 - 4.55)	
ADHD	0.89 (0.80 - 1.00)	0.99 (0.82 - 1.20)		1.83 (1.07 - 3.12)*	0.66 (0.12 - 3.48)	
**Number of co-occuring Common Mental Disorders experienced**						
1 Other Disorder	0.95 (0.87 - 1.03)	1.10 (0.95 - 1.28)		1.88 (0.62 - 5.65)	1.31 (0.00 - 1637.48)	
2 Other Disorders	0.84 (0.75 - 0.95)*	1.11 (0.83 - 1.48)		3.73 (1.28 - 10.89)*	2.50 (0.00 - 3147.70)	
3 or more Other Disorders	0.76 (0.61 - 0.96)*	1.03 (0.63 - 1.66)		5.06 (1.59 - 16.09)*	4.17 (0.00 - 6903.64)	
Number of Disorders experienced: F (df, p)	4.13 (3, p = 0.007)	1.14 (3, p = 0.334)		4.06 (3, p = 0.007)	0.05 (3, p = 0.987)	

Abbreviations: RR, Risk Ratio, 95% CI, 95% Confidence Interval

Risk ratios and results are from multivariable Poisson distribution and robust standard errors. Data was multiply imputed, m = 20.

All univariable models controlled for year of survey and incentivisation. All multivariable models controlled for all variables included in the model, and for year of survey and incentivisation

* denotes significance difference, p < .05.

Of greatest interest was that, in the fully adjusted 12-month models, the risk of suicidal ideation and plans for non-heterosexual respondents increased by about 10% relative to the effect sizes observed in the lifetime models (ideation, RR: 1.50 [95% CI: 1.35–1.67], plans, RR: 1.72 [95% CI: 1.40–2.11]) yet, sexual orientation was no longer associated with risk of suicide attempt (past 12 months, RR: 1.74 [95% CI: 0.96–3.15] vs. lifetime, RR: 1.45 [95% CI: 1.10–1.90]). Similarly, the effect sizes also increased for the associations for MDD and BP, most notably for risk of suicide attempt where the risk doubled (MDD, RR: 3.65 [95% CI: 1.37–9.73] vs. RR: 1.80, [95% CI: 1.23–2.64]; BP, RR: 7.34 [95% CI: 2.20–24.45.20.45] vs. RR: 3.07 [95% CI: 1.99–4.73]). Notably, PTSD was not associated with risk of attempting suicide in the past 12 months, despite being one of the strongest predictors of lifetime suicide attempts (RR: 2.07 [95% CI: 0.94–4.57] vs. RR: 3.03 [95% CI: 1.77–5.20]).

### Associations of socio-demographics variables and mental disorders with persistence of suicidal thoughts and behaviours

Again, we see a similar pattern for 12-month as for the lifetime models when testing associations between socio-demographics and persistence of suicidal ideation (see Table 5, and Supplementary Table C for sequential models). Non-heterosexual orientation and experiences of MDD and BP were associated with persistence of suicidal thoughts across univariable models and, to a lesser degree, multivariable models (e.g., non-heterosexual, univariable, RR: 1.22 [95% CI: 1.11–1.34] vs. multivariable, RR: 1.12 [95% CI: 1.02–1.23]). With the exception of AUD, all mental disorders were associated with greater risk of persistent suicidal ideation in univariable models; the effects for PD (RR: 1.30 [95% CI: 1.20–1.46] vs. RR: 1.05 [95% CI: 0.94–1.17]), PTSD (RR: 1.34 [95% CI: 1.21–1.49] vs. RR: 1.10 [95% CI: 0.98–1.23]), DUD (RR: 1.19 [95% CI: 1.05–1.33] vs. RR: 1.06 [95% CI: 0.93–1.21]), and ADHD (RR: 1.23 [95% CI: 1.11–1.37] vs. RR: 0.99 [95% CI: 0.78–1.26]) did not continue across multivariable models, however. In contrast to the lifetime models, older age was found to be associated with reduced persistence of suicidal thoughts (RR:0.84, 95% CI: 0.74–0.95) while experiences of GAD were found to increase risk of suicidal thoughts (RR:1.11, 95% CI:1.01–1.22). For persistence of suicide plans, only co-occurring BP was found to be associated with increased risk (RR:1.42, 95% CI: 1.06–1.92) and for suicide attempts, no statistically significant associations were observed.

**Table 5:** The associations of demographics variables and past 12-month experiences of common mental disorders with persistence of suicide-related outcomes in the past 12 month, given lifetime cases, RR (95% CI)

	Suicidal Ideation		Suicide Plan		Suicide Attempt	
	Univariable	Multivariable	Univariable	Multivariable	Univariable	Multivariable
Age (Ref: 18 & Under)						
19	0.96 (0.85–1.08)	0.96 (0.85–1.08)	0.99 (0.81–1.22)	0.98 (0.80–1.19)	0.71 (0.25–2.03)	0.66 (0.26–1.67)
20 & Older	0.84 (0.74–0.95)*	0.84 (0.75–0.95)*	0.90 (0.74–1.09)	0.87 (0.72–1.05)	0.72 (0.31–1.67)	0.74 (0.38–1.44)
Age Category: F (df, p)	3.29 (2, *p* = 0.038)	3.61 (2, *p* = 0.028)	0.52 (2, *p* = 0.592)	0.94 (2, *p* = 0.391)	0.43 (2, *p* = 0.652)	0.61 (2, *p* = 0.543)
Gender (Ref: Female)						
Male	0.95 (0.85–1.06)	1.06 (0.95–1.18)	0.87 (0.71–1.07)	0.97 (0.79–1.20)	0.82 (0.31–2.17)	0.98 (0.42–2.31)
Gender Queer	1.23 (1.05–1.44)*	0.99 (0.77–1.28)	1.13 (0.85–1.50)	0.81 (0.56–1.17)	1.44 (0.60–3.44)	0.55 (0.07–4.49)
Gender: F (df, p)	4.39 (2, *p* = 0.013)	0.62 (2, *p* = 0.540)	1.35 (2, *p* = 0.260)	0.58 (2, *p* = 0.560)	0.45 (2, *p* = 0.635)	0.16 (2, *p* = 0.849)
Gender Modality (Ref: Cisgender)						
Transgender	1.27 (1.11–1.46)*	1.08 (0.86–1.36)	1.26 (1.00–1.58)	1.26 (0.93–1.73)	1.47 (0.69–3.13)	1.87 (0.27–13.12)
Sexual Orientation (Ref: Non-heterosexual)						
Non-heterosexual	1.22 (1.11–1.34)*	1.12 (1.02–1.23)*	1.24 (1.06–1.45)*	1.10 (0.94–1.29)	1.61 (0.84–3.10)	1.30 (0.65–2.59)
Enrolment Status (Ref: Full time)						
Part-time	0.86 (0.69–1.08)	0.93 (0.75–1.15)	1.02 (0.71–1.47)	0.96 (0.68–1.36)	0.42 (0.00–643.33)	0.84 (0.26–2.73)
Parental Education (Ref: High School or Less)						
University or higher	1.00 (0.90–1.11)	0.99 (0.90–1.09)	1.02 (0.86–1.21)	1.02 (0.88–1.18)	0.80 (0.42–1.52)	0.75 (0.38–1.50)
Ethnicity (Ref: Pākehā/European)						
Māori	1.08 (0.92–1.27)	1.00 (0.87–1.15)	1.12 (0.85–1.47)	1.06 (0.84–1.35)	1.32 (0.54–3.23)	1.06 (0.48–2.31)
Pacific Peoples	1.04 (0.83–1.31)	1.01 (0.83–1.23)	1.09 (0.70–1.69)	1.05 (0.71–1.54)	1.84 (0.50–6.71)	1.83 (0.59–5.71)
Asian	0.95 (0.82–1.09)	1.04 (0.90–1.19)	1.00 (0.78–1.28)	1.06 (0.83–1.35)	0.82 (0.29–2.36)	1.37 (0.47–3.97)
MELAA	1.07 (0.85–1.34)	1.01 (0.82–1.25)	1.16 (0.76–1.77)	1.09 (0.73–1.63)	0.14 (0.00–2913.43)	0.33 (0.00–960.46)
Ethnicity: F (df, p)	0.57 (4, *p* = 0.686)	0.07 (4, *p* = 0.990)	0.29 (4, *p* = 0.883)	0.13 (4, *p* = 0.970)	0.17 (4, *p* = 0.955)	0.14 (4, *p* = 0.966)
Course (Ref: Arts & Sciences)						
Medical Sciences	0.95 (0.80–1.13)	0.91 (0.77–1.06)	0.97 (0.70–1.33)	0.92 (0.69–1.23)	1.00 (0.36–2.78)	0.83 (0.31–2.17)
Commerce	1.04 (0.93–1.17)	1.02 (0.91–1.13)	1.13 (0.94–1.35)	1.09 (0.92–1.30)	0.86 (0.38–1.97)	0.85 (0.39–1.85)
Laws	1.03 (0.81–1.29)	1.01 (0.85–1.21)	1.13 (0.85–1.51)	1.12 (0.84–1.48)	1.29 (0.50–3.36)	1.44 (0.56–3.70)
Course: F (df, p)	0.34 (3, *p* = 0.794)	0.61 (3, *p* = 0.610)	0.78 (3, *p* = 0.507)	0.66 (3, *p* = 0.574)	0.16 (3, *p* = 0.921)	0.36 (3, *p* = 0.779)
Experiences of co-occuring Common Mental Disorders						
MDD	1.34 (1.21–1.48)*	1.18 (1.04–1.35)*	1.35 (1.13–1.62)*	1.31 (1.00–1.72)	1.04 (0.51–2.12)	1.95 (0.63–6.06)
BP	1.31 (1.13–1.52)*	1.20 (1.02–1.40)*	1.37 (1.10–1.71)*	1.42 (1.06–1.92)*	1.93 (0.83–4.48)	2.73 (0.76–9.85)
GAD	1.36 (1.24–1.48)*	1.11 (1.01–1.22)*	1.39 (1.16–1.67)*	1.12 (0.94–1.33)	1.91 (0.93–3.95)	1.20 (0.61–2.37)
PD	1.32 (1.20–1.46)*	1.05 (0.94–1.17)	1.30 (1.00–1.67)	1.03 (0.86–1.23)	2.04 (0.92–4.54)	1.51 (0.73–3.15)
PTSD	1.34 (1.21–1.49)*	1.10 (0.98–1.23)	1.34 (1.09–1.65)*	1.06 (0.86–1.31)	2.81 (1.04–7.57)*	1.87 (0.72–4.86)
DUD	1.19 (1.05–1.33)*	1.06 (0.93–1.21)	1.24 (1.02–1.49)*	1.15 (0.93–1.43)	1.72 (0.88–3.38)	1.13 (0.58–2.20)
AUD	1.09 (0.98–1.21)	0.92 (0.74–1.15)	1.00 (0.83–1.20)	0.80 (0.55–1.16)	1.66 (0.81–3.40)	0.82 (0.17–4.03)
ADHD	1.23 (1.11–1.37)*	0.99 (0.78–1.26)	1.27 (1.07–1.51)*	0.92 (0.59–1.42)	1.69 (0.89–3.21)	0.61 (0.07–5.25)
Number of co-occuring Common Mental Disorders experienced						
1 Other Disorder	1.34 (1.16–1.54)*	1.20 (0.91–1.58)	1.24 (0.88–1.73)	1.12 (0.73–1.72)	1.37 (0.34–5.41)	1.84 (0.00–50391.69)
2 Other Disorders	1.58 (1.37–1.81)*	1.29 (0.85–1.96)	1.70 (1.29–2.25)*	1.38 (0.73–2.64)	2.76 (0.80–9.50)	3.26 (0.00–83002.22)
3 or more Other Disorders	1.64 (1.38–1.94)*	1.35 (0.73–2.49)	1.71 (1.23–2.38)*	1.45 (0.54–3.91)	2.71 (0.61–12.02)	5.58 (0.00–190282.84)
Number of Disorders experienced: F (df, p)	15.74 (3, *p* < 0.001)	0.74 (3, *p* = 0.527)	6.4 (3, *p* = 0.000)	0.56 (3, *p* = 0.639)	1.79 (3, *p* = 0.149)	0.03 (3, *p* = 0.992)

Abbreviations: RR, Risk Ratio, 95% CI, 95% Confidence Interval

Risk ratios and results are from multivariable Poisson distribution and robust standard errors. Data was multiply imputed, m = 20

All univariable models controlled for year of survey and incentivisation. All multivariable models controlled for all variables included in the model, and for year of survey and incentivisation

* denotes significance difference, *p* < 0.05

## Discussion

The present study presents data collected in New Zealand as part of the WHO WMH-ICS initiative. Approximately one-third of first-year university students reported having suicidal thoughts during the past 12 months, with approximately one-fifth reporting a suicide plan, and three-in-every-hundred reporting a suicide attempt. These findings provide critical epidemiological evidence about suicide-related outcomes within a university sample in New Zealand.

Overall, our prevalence estimates are marginally elevated relative to recent pooled cross-national estimates among first-year university students [6] yet considerably higher than those observed in earlier cross-national estimates [2] or Te Rau Hinengaro, the only nationally representative data, collected over two decades ago [20]. While this may reflect natural increases over time, coronial data indicates that youth suicide rates in New Zealand have been relatively stable over this period [19]. Likewise, relatively recent data from the Australian National Study of Mental Health and Wellbeing (2020–2022) [35] is somewhat equivalent to the earlier Te Rau Hinengaro [20] findings across comparable estimates, despite a 20-year difference in data collection periods (2003-4 vs. 2020-22; 15.7% vs. 16.7%).

In considering why the rates of suicidal thoughts and behaviours appear to be increasing over time, it is important to acknowledge the impact of the COVID-19 pandemic. Although New Zealand’s experience of the pandemic was relatively unique compared to many other parts of the world, particularly other countries included in the reviewed cross-national studies, it has likely impacted young people’s academic and social developmental trajectories [36, 37]. This may alter their ability to respond to the changing academic and social landscapes associated with the transition to university, and adjustment that is already known to cause stress and increase vulnerability to negative psychological health outcomes [38]. Unfortunately, as our data was collected in a pandemic/post-pandemic context, we lack the capacity to explicitly consider whether the prevalence of suicide-related outcomes differ as a function of the COVD-19 pandemic.

An increase in suicide-related outcomes may also reflect increases in general distress among students and young people. Cross-national estimates suggest that two-thirds of first-year university students screened positive for at least one lifetime common mental disorder [21] and there are growing concerns about a youth mental health epidemic [39–42]. Given the increased risk of suicide-related outcomes among those with a co-occurring mental disorder, especially for individuals who had experienced greater progression along the suicidal trajectory, it is reasonable to consider that elevations in general distress among students may account, at least partially, for the observed elevations in prevalence of suicide-related outcomes.

Finally, response rates may also account for some of the variation between studies. While the achieved response rates (overall response rate, 24.2%; unincentivised sample, 16.7%, incentivized sample, 40.5%) are comparable to other university student survey samples exploring suicide-related outcomes [6], they are far lower than nationally representative surveys (e.g., Te Rau Hinengaro, 73%) [43]. Our comparatively low response rate may distort the accuracy of the estimates by increasing the risk for non-response bias [44, 45]. However, differences between the current sample and Te Rau Hinengaro are also likely explained by variation between university and non-university samples [46].

The consistency and commonality of associations demonstrating that individuals who belong to gender or sexual minorities are at greater risk of suicide-related outcomes highlights a clear imperative for innovative and targeted intervention [47, 48]. These associations are likely explained by Minority Stress Theory, which posits higher risk of mental health problems as a result of exposure to multiple experiences of discrimination with reduced social support to buffer such stressors [49]. The complex nature of challenging these structural, socioeconomic, and political determinants for suicide prevention requires connection and partnership between government policies and public health approaches to ensure effective, population-level interventions are delivered [50–52].

Interestingly, of all the ethnicity-based comparisons we made, the only association that was statistically significant within the full models was that Asian respondents were more likely to experience suicidal ideation, relative to their European peers. This was surprising given that university students from Indigenous populations are consistently shown to be more vulnerable to suicide thoughts and behaviours [53, 54] and, within New Zealand, Māori and Pacific peoples both have significantly higher rates of suicide than their European peers [19]. One explanation may be the use of priority coding for ethnicity which precludes identification with multiple ethnicities and thus, may mask associations for individuals who identify across multiple prioritised ethnicities where the impacts of minority stress may be exacerbated [54]. Alternatively, it may also reflect broader inequities between university and non-university populations.

Finally, age-of-onset was found to typically be in mid-adolescence for all suicide-related outcomes. This matches cross-national patterns among university students [2] and aligns with adolescent data showing relatively high proportions of 12-month suicide-related outcomes during the high-school period in New Zealand [55]. Interestingly, the only age-related finding observed within the multivariable models was that students aged 20 years or older were less likely to have persistent suicidal thoughts than their younger peers (aged 18 or under). Further, our findings clearly suggest that over half of first-year students have likely experienced suicidal thoughts in their lifetime and that two-thirds of these students are likely to continue to experience persistent suicidal thoughts within the past year. These findings demonstrate that suicide-related outcomes among university students cannot be seen as just a ‘university’ or ‘young adult’ problem, but rather, as a problem that begins in adolescence and persists into young adulthood.

### Strengths and limitations

The current study achieved a large sample (*n =* 3,702) and relatively strong recruitment among priority communities (response rate = 39.3% of all first year Māori and Pacific peoples students; overall response rate:24.2%). However, some limitations must be noted which may impact the accuracy of our estimations. Our results rely on self-report screening instruments to assess suicide-related outcomes. Although the items were drawn from well-validated measures, there was no objective measurement of what defines a suicide attempt or plan. Similarly, the current approach to imputation meant that we could not distinguish between passive and active suicidal ideation. Further, these data are cross-sectional and rely on retrospective recall across the person’s lifetime or past 12 months.

## Conclusions

To improve suicide prevention about young adults in New Zealand, it is imperative that we better understand the epidemiology of suicide within this context so that we may (1) accurately comprehend the extent of the issue and (2) create baseline prevalence estimates that enable measurement of change and the evaluation strategies to reduce university student suicide in New Zealand. Our findings provide this groundwork and emphasise the need to develop holistic, population-level approaches that increase awareness and support for students who may have increased vulnerability to suicide-related outcomes alongside best practice, individual-level approaches to care that focus on robust psychosocial assessment and provision of quality care [56, 57].

## Supplementary Information

Below is the link to the electronic supplementary material.

Supplementary Material 1

## Data Availability

The data analysed in this study are subject to the following licenses/restrictions: The WMH-ICS data sharing agreement limits access of this data to members of the consortium. The participant data and statistical analysis plan used for this study are available upon reasonable request to author, Rapsey, as long as the main objective of the data sharing request is replicating the analysis and findings as reported in this paper.

## References

[1] Crispim MO, Santos C, Frazao IDS et al (2021) Prevalence of suicidal behavior in young university students: A systematic review with meta-analysis. Rev Lat Am Enfermagem 29:e3495 34755776 10.1590/1518-8345.5320.3495 PMC8584877

[2] Mortier P, Auerbach RP, Alonso J et al (2018b) Suicidal thoughts and behaviors among First-Year college students: results from the WMH-ICS project. J Am Acad Child Adolesc Psychiatry 57(4):263–273e261 29588052 10.1016/j.jaac.2018.01.018 PMC6444360

[3] Mortier P, Cuijpers P, Kiekens G et al (2018c) The prevalence of suicidal thoughts and behaviours among college students: a meta-analysis. Psychol Med 48(4):554–565 28805169 10.1017/S0033291717002215

[4] Sivertsen B, Hysing M, Knapstad M et al (2019) Suicide attempts and non-suicidal self-harm among university students: prevalence study. BJPsych Open 5(2):e26 31068238 10.1192/bjo.2019.4 PMC6401540

[5] Cuijpers P, Auerbach RP, Benjet C et al (2019) The World Health Organization World Mental Health International College Student initiative: an overview. Int J Methods Psychiatr Res 28(2):e1761 30614123 10.1002/mpr.1761 PMC6590455

[6] Mortier P, Yang X, Altwaijri YA et al (2025) The associations of childhood adversities and mental disorders with suicidal thoughts and behaviors - results from the World Mental Health International College Student Initiative. Psychiatr Res. 10.1016/j.psychres.2025.116555 PMC12240005 40450963

[7] Auerbach RP, Mortier P, Bruffaerts R et al (2019) Mental disorder comorbidity and suicidal thoughts and behaviors in the world health organization world mental health surveys international college student initiative. Int J Methods Psychiatr Res 28(2):e1752 30450753 10.1002/mpr.1752 PMC6877246

[8] Bantjes J, Kessler MJ, Hunt X et al (2023) Prevalence and correlates of 30-day suicidal ideation and intent: results of the South African National student mental health survey. S Afr Med J 113(4):e16753 37283153 10.7196/SAMJ.2023.v113i4.16753

[9] Mortier P, Demyttenaere K, Auerbach RP et al (2017) First onset of suicidal thoughts and behaviours in college. J Affect Disord 207:291–299 27741465 10.1016/j.jad.2016.09.033 PMC5460371

[10] O’Neill S, McLafferty M, Ennis E et al (2018) Socio-demographic, mental health and childhood adversity risk factors for self-harm and suicidal behaviour in college students in Northern Ireland. J Affect Disord 239:58–65 29990663 10.1016/j.jad.2018.06.006

[11] Renteria R, Benjet C, Gutierrez-Garcia RA et al (2021) Suicide thought and behaviors, non-suicidal self-injury, and perceived life stress among sexual minority Mexican college students. J Affect Disord 281:891–898 33243555 10.1016/j.jad.2020.11.038 PMC7856251

[12] Ward C, McLafferty M, McLaughlin J et al (2022) Suicidal behaviours and mental health disorders among students commencing college. Psychiatry Res 307:114314 34864232 10.1016/j.psychres.2021.114314

[13] McLafferty M, Brown N, Brady J, McLaughlin J, McHugh R, Ward C et al (2022) Variations in psychological disorders, suicidality, and help-seeking behaviour among college students from different academic disciplines. PLoS One 17(12):e0279618. 10.1371/journal.pone.0279618 36584170 PMC9803302

[14] Crossin R, Cleland L, McLeod GF et al (2022) The association between alcohol use disorder and suicidal ideation in a new Zealand birth cohort. Aust N Z J Psychiatry 56(12):1576–1586 34903072 10.1177/00048674211064183

[15] Sivertsen B, O’Connor RC, Nilsen SA et al (2024) Mental health problems and suicidal behavior from adolescence to young adulthood in college: linking two population-based studies. Eur Child Adolesc Psychiatry 33(2):421–429 36843045 10.1007/s00787-023-02167-y PMC10869414

[16] Soto-Sanz V, Castellvi P, Piqueras JA et al (2019) Internalizing and externalizing symptoms and suicidal behaviour in young people: a systematic review and meta-analysis of longitudinal studies. Acta Psychiatr Scand 140(1):5–19 30980525 10.1111/acps.13036

[17] Commisso M, Temcheff C, Orri M et al (2023) Childhood externalizing, internalizing and comorbid problems: distinguishing young adults who think about suicide from those who attempt suicide. Psychol Med 53(3):1030–1037 34183077 10.1017/S0033291721002464

[18] Owusu-Ansah FE, Addae AA, Peasah BO et al (2020) Suicide among university students: prevalence, risks and protective factors. Health Psychol Behav Med 8(1):220–233 34040869 10.1080/21642850.2020.1766978 PMC8114407

[19] Te Whatu Ora (2024) Suicide Web Tool

[20] Beautrais AL, Wells JE, McGee MA et al (2006) Suicidal behaviour in Te Rau Hinengaro: the New Zealand mental health survey. Aust N Z J Psychiatry 40(10):896–904 16959016 10.1080/j.1440-1614.2006.01909.x

[21] Mason A, Rapsey C, Sampson N et al (2025) Prevalence, age-of-onset, and course of mental disorders among 72,288 first-year university students from 18 countries in the world mental health international college student (WMH-ICS) initiative. J Psychiatr Res 183:225–236 40010072 10.1016/j.jpsychires.2025.02.016 PMC11926851

[22] Posner K, Brown GK, Stanley B et al (2011) The Columbia-Suicide severity rating scale: initial validity and internal consistency findings from three multisite studies with adolescents and adults. Am J Psychiatry 168(12):A28–1362 10.1176/appi.ajp.2011.10111704 PMC3893686 22193671

[23] Kessler RC, Calabrese JR, Farley PA et al (2013) Composite international diagnostic interview screening scales for DSM-IV anxiety and mood disorders. Psychol Med 43(8):1625–1637 23075829 10.1017/S0033291712002334

[24] Weathers FW, Keane BTLTM, Palmieri PA, Marx BP, Schnurr PP (2013) PTSD checklist for DSM-5 (PCL-5). Scale available from the National Center for PTSD at

[25] Kessler RC, Adler LA, Gruber MJ et al (2007) Validity of the world health organization adult ADHD Self-Report scale (ASRS) screener in a representative sample of health plan members. Int J Methods Psychiatr Res 16(2):52–65 17623385 10.1002/mpr.208 PMC2044504

[26] Babor TF, Higgins-Biddle JC, Saunders JB et al (1992) AUDIT: the alcohol use disorders identification test: guidelines for use in primary health care. Reportno. Report Number|, date. Institution|, Place Published|

[27] DeBell M, Krosnick JA (2009) Computing weights for American National election study survey data. Reportno. Report Number|, date. Institution|, Place Published|

[28] Rudkin A (2025) autumn: Fast, Modern, and Tidy-Friendly Iterative Raking. R package version 0.1 ed

[29] Atkinson J, Salmond C, Crampton P et al (2024) NZDep2023 index of socioeconomic deprivation: research Report, 31 October 2024. Reportno. Report Number|, date. Institution|, Place Published|

[30] Mayer M (2023) missRanger: Fast imputation of missing values

[31] Lumley T (2004) Analysis of complex survey samples. J Stat Softw 9(1):1–19

[32] Lumley T (2024) survey: analysis of complex survey samples. R package version 4.4. ed

[33] Lumley T, Kronmal R, Ma S (2006) Relative risk regression in medical research: Models, Contrasts, estimators and Algorithms. Reportno. Report Number|, date. Institution|, Place Published|

[34] Zou G (2004) A modified poisson regression approach to prospective studies with binary data. Am J Epidemiol 159(7):702–706 15033648 10.1093/aje/kwh090

[35] Australian Bureau of Statistics (2020–2022) *Natiuonal Study of Mental Health and Wellbeing*. Available at: https://www.abs.gov.au/statistics/health/mental-health/national-study-mental-health-and-wellbeing/latest-release

[36] Matsuo H, Bruce M, Homvisetvongsa S et al (2024) The impact of COVID-19 on University Students’ Academic Motivation, Social Connection, and Psychological Well-Being. Sch Teach Learn Psychol 10(3):320–330

[37] Pasupathi M, Booker J, Ell M et al (2022) College, interrupted: profiles in first-year college students responses to the COVID-19 pandemic across one year. Emerg Adulthood 10(6):1574–1590 38603297 10.1177/21676968221119945 PMC9535459

[38] Karyotaki E, Cuijpers P, Albor Y et al (2020) Sources of stress and their associations with mental disorders among college students: results of the World Health Organization world mental health surveys international college student initiative. Front Psychol 11:1759 32849042 10.3389/fpsyg.2020.01759 PMC7406671

[39] Beresin EV, Guerrero APS, Morreale MK et al (2024) Addressing the youth mental health epidemic. Acad Psychiatry 48(3):217–221 38632217 10.1007/s40596-024-01967-x

[40] McGorry PD, Mei C, Dalal N et al (2024) The lancet psychiatry commission on youth mental health. Lancet Psychiatry 11(9):731–774 39147461 10.1016/S2215-0366(24)00163-9

[41] Mulder RT, Bastiampillai T, Jorm A et al (2022) New zealand’s mental health crisis, he Ara Oranga and the future. N Z Med J 135(1548):89–93 35728133

[42] Shim R, Szilagyi M, Perrin JM (2022) Epidemic rates of child and adolescent mental health disorders require an urgent response. Pediatrics 149(5):e2022056611. 10.1542/peds.2022-056611 35202473

[43] Wells JE, Oakley Browne MA, Scott KM et al (2006) Te Rau hinengaro: the new Zealand mental health survey: overview of Methdos and findings. Australian New Z J Psychiatry 40(10):835–844 16959009 10.1080/j.1440-1614.2006.01902.x

[44] Groves RM, Peytcheva E (2008) The impact of nonresponse rates on nonresponse bias: a meta-analysis. Pub Opin Q 72(2):167–189

[45] Rindfuss RR, Choe MK, Tsuya NO et al (2015) Do low survey response rates bias results? Evidence from Japan. Demogr Res 32:797–828

[46] Mortier P, Auerbach RP, Alonso J et al (2018a) Suicidal thoughts and behaviors among college students and same-aged peers: results from the world health organization world mental health surveys. Soc Psychiatry Psychiatr Epidemiol 53(3):279–288 29340781 10.1007/s00127-018-1481-6 PMC5896296

[47] Busby DR, Horwitz AG, Zheng K et al (2020) Suicide risk among gender and sexual minority college students: the roles of victimization, discrimination, connectedness, and identity affirmation. J Psychiatr Res 121:182–188 31837538 10.1016/j.jpsychires.2019.11.013 PMC7008002

[48] Horwitz AG, Berona J, Busby DR et al (2020) Variation in suicide risk among subgroups of sexual and gender minority college students. Suicide Life Threat Behav 50(5):1041–1053 32291833 10.1111/sltb.12637 PMC7981781

[49] Frost DM, Meyer IH (2023) Minority stress theory: application, critique, and continued relevance. Curr Opin Psychol 51:101579 37270877 10.1016/j.copsyc.2023.101579 PMC10712335

[50] Marzetti H, Cooper C, Mason A et al (2024) LGBTQ+ suicide - a call to action for researchers and governments on the politics, practices, and possibilities of LGBTQ+ suicide prevention. Crisis 45(2):87–92 38487858 10.1027/0227-5910/a000950

[51] Pirkis J, Dandona R, Silverman M et al (2024) Preventing suicide: a public health approach to a global problem. Lancet Public Health 9(10):e787–e795 39265611 10.1016/S2468-2667(24)00149-X

[52] Pirkis J, Gunnell D, Hawton K et al (2023) A public health, whole-of-government approach to National suicide prevention strategies. Hogrefe Publishing, pp 85–92 10.1027/0227-5910/a000902 36960842

[53] Hop Wo NK, Anderson KK, Wylie L et al (2020) The prevalence of distress, depression, anxiety, and substance use issues among Indigenous post-secondary students in Canada. Transcult Psychiatry 57(2):263–274 31575332 10.1177/1363461519861824

[54] Shepherd BF, Kelly LM, Brochu PM et al (2023) An examination of theory-based suicidal ideation risk factors in college students with multiple marginalized identities. Am J Orthopsychiatry 93(2):107–119 36913274 10.1037/ort0000666 PMC10015593

[55] Fleming T, Ball J, Bavin L et al (2022) Mixed progress in adolescent health and wellbeing in Aotearoa New Zealand 2001–2019: a population overview from the Youth2000 survey series. J R Soc N Z 52(4):426–449 39440321 10.1080/03036758.2022.2072349 PMC11485765

[56] Fortune S (2024) Time for a more evidence-based approach to suicide prevention. N Z Med J 29(137):9–12. 10.26635/6965.e1606 39607959

[57] Fortune S, Hetrick S (2022) Suicide risk assessments: why are we still relying on these a decade after the evidence showed they perform poorly? Aust N Z J Psychiatry 56(12):1529–1534 35786014 10.1177/00048674221107316

