# Supplementary material for: Prevalence and associations of lifetime, 12-month, and 30-day suicidal thoughts and behaviours among 3,702 First-Year university students in New Zealand
Source: Soc Psychiatry Psychiatr Epidemiol. 2026 Feb 27;61(9):1813–31. doi: 10.1007/s00127-026-03069-5 (PMC13578058; doi:10.1007/s00127-026-03069-5)
Supplement: Supplementary file 1 [file 127_2026_3069_MOESM1_ESM.docx]

Supplementary Table A: The associations of demographics variables and common mental disorders with lifetime suicide-related outcomes and transition along the suicidal trajectory, RR (95% CI)

|  | Suicidal Ideation |  |  |  | Suicide Plan |  |  |  |
| --- | --- | --- | --- | --- | --- | --- | --- | --- |
|  | Univariable | Block 1 | Block 2 | Block 3 | Univariable | Block 1 | Block 2 | Block 3 |
| **Age (Ref: 18 & Under)** |  |  |  |  |  |  |  |  |
| 19 | 0.96 (0.87 - 1.06) | 0.97 (0.88 - 1.06) | 0.94 (0.86 - 1.02) | 0.93 (0.85 - 1.02) | 0.96 (0.80 - 1.15) | 0.96 (0.81 - 1.13) | 0.90 (0.77 - 1.05) | 0.90 (0.77 - 1.05) |
| 20 & Older | 1.09 (0.99 - 1.19) | 1.07 (0.98 - 1.17) | 1.02 (0.94 - 1.11) | 1.01 (0.93 - 1.10) | 1.07 (0.90 - 1.28) | 1.08 (0.91 - 1.28) | 0.96 (0.81 - 1.13) | 0.95 (0.81 - 1.12) |
| Age Category: F (df, p) | 2.25 (2, p = 0.105) | 1.61 (2, p = 0.199) | 1.23 (2, p = 0.292) | 1.45 (2, p = 0.234) | 0.45 (2, p = 0.636) | 0.56 (2, p = 0.569) | 0.86 (2, p = 0.425) | 1.01 (2, p = 0.366) |
| **Gender (Ref: Female)** |  |  |  |  |  |  |  |  |
| Male | **0.78 (0.71 - 0.85)*** | **0.85 (0.77 - 0.93)*** | 0.99 (0.91 - 1.08) | 1.00 (0.92 - 1.09) | **0.73 (0.60 - 0.88)*** | **0.81 (0.68 - 0.97)*** | 1.07 (0.91 - 1.27) | 1.08 (0.91 - 1.27) |
| Gender Queer | **1.52 (1.40 - 1.64)*** | 0.91 (0.75 - 1.11) | 1.04 (0.86 - 1.25) | 1.04 (0.87 - 1.23) | **2.34 (1.80 - 3.05)*** | 0.94 (0.65 - 1.35) | 1.11 (0.78 - 1.59) | 1.11 (0.78 - 1.58) |
| Gender: F (df, p) | 82.53 (2, p < 0.001) | 6.18 (2, p = 0.002) | 0.14 (2, p = 0.872) | 0.10 (2, p = 0.902) | 37.25 (2, p < 0.001) | 2.96 (2, p = 0.052) | 0.36 (2, p = 0.701) | 0.41 (2, p = 0.663) |
| **Gender Modality (Ref: Cisgender)** |  |  |  |  |  |  |  |  |
| Transgender | **1.65 (1.53 - 1.78)*** | **1.23 (1.01 - 1.48)*** | 0.95 (0.80 - 1.12) | 0.96 (0.82 - 1.12) | **2.60 (2.04 - 3.31)*** | **1.55 (1.11 - 2.15)*** | 1.08 (0.79 - 1.49) | 1.10 (0.81 - 1.50) |
| **Sexual Orientation (Ref: Non-heterosexual)** |  |  |  |  |  |  |  |  |
| Non-heterosexual | **1.73 (1.62 - 1.84)*** | **1.64 (1.53 - 1.76)*** | **1.40 (1.30 - 1.50)*** | **1.40 (1.31 - 1.50)*** | **2.40 (1.98 - 2.91)*** | **2.11 (1.83 - 2.44)*** | **1.61 (1.39 - 1.87)*** | **1.62 (1.41 - 1.87)*** |
| **Enrolment Status (Ref: Full time)** |  |  |  |  |  |  |  |  |
| Part-time | 1.14 (0.98 - 1.31) | 1.15 (0.99 - 1.32) | 1.04 (0.90 - 1.19) | 1.05 (0.92 - 1.21) | 0.96 (0.70 - 1.32) | 0.99 (0.73 - 1.35) | 0.85 (0.65 - 1.12) | 0.86 (0.66 - 1.14) |
| **Parental Education (Ref: High School or Less)** |  |  |  |  |  |  |  |  |
| University or higher | 0.98 (0.90 - 1.06) | 0.97 (0.89 - 1.04) | 1.00 (0.93 - 1.07) | 1.00 (0.93 - 1.07) | 0.97 (0.83 - 1.12) | 0.95 (0.83 - 1.10) | 1.02 (0.89 - 1.16) | 1.01 (0.89 - 1.16) |
| **Ethnicity (Ref: Pākehā)** |  |  |  |  |  |  |  |  |
| Māori | 0.98 (0.86 - 1.10) | 0.99 (0.88 - 1.10) | 0.92 (0.82 - 1.02) | 0.91 (0.82 - 1.01) | 1.15 (0.92 - 1.45) | 1.17 (0.94 - 1.45) | 1.01 (0.83 - 1.24) | 1.01 (0.83 - 1.24) |
| Pacific Peoples | 0.98 (0.82 - 1.17) | 1.03 (0.87 - 1.22) | 0.98 (0.84 - 1.14) | 1.00 (0.86 - 1.16) | 1.01 (0.70 - 1.46) | 1.12 (0.81 - 1.56) | 1.01 (0.74 - 1.36) | 1.03 (0.76 - 1.39) |
| Asian | 1.01 (0.91 - 1.13) | 1.09 (0.98 - 1.21) | **1.14 (1.03 - 1.26)*** | **1.17 (1.06 - 1.30)*** | 0.90 (0.72 - 1.12) | 1.02 (0.84 - 1.25) | 1.10 (0.91 - 1.33) | 1.13 (0.93 - 1.37) |
| MELAA | 0.96 (0.77 - 1.21) | 1.01 (0.81 - 1.27) | 0.98 (0.82 - 1.17) | 1.02 (0.85 - 1.21) | 1.09 (0.72 - 1.66) | 1.19 (0.81 - 1.75) | 1.11 (0.78 - 1.56) | 1.15 (0.82 - 1.62) |
| Ethnicity: F (df, p) | 0.09 (4, p = 0.986) | 0.77 (4, p = 0.547) | 2.61 (4, p = 0.034) | 3.43 (4, p = 0.008) | 0.86 (4, p = 0.487) | 0.72 (4, p = 0.578) | 0.27 (4, p = 0.894) | 0.50 (4, p = 0.739) |
| **Course (Ref: Arts & Sciences)** |  |  |  |  |  |  |  |  |
| Medical Sciences | **0.84 (0.73 - 0.96)*** | 0.88 (0.78 - 1.01) | 0.89 (0.79 - 1.00) | 0.90 (0.80 - 1.02) | **0.71 (0.55 - 0.92)*** | **0.75 (0.59 - 0.97)*** | **0.77 (0.61 - 0.96)*** | **0.77 (0.61 - 0.98)*** |
| Commerce | **1.16 (1.07 - 1.27)*** | **1.13 (1.04 - 1.23)*** | **1.10 (1.02 - 1.19)*** | **1.10 (1.02 - 1.19)*** | **1.27 (1.07 - 1.52)*** | **1.21 (1.03 - 1.41)*** | 1.15 (0.99 - 1.34) | 1.15 (0.99 - 1.34) |
| Laws | **1.30 (1.14 - 1.47)*** | **1.15 (1.01 - 1.30)*** | 1.14 (1.00 - 1.29) | 1.14 (1.00 - 1.29) | **1.46 (1.11 - 1.91)*** | 1.19 (0.92 - 1.55) | 1.19 (0.92 - 1.53) | 1.19 (0.93 - 1.53) |
| Course: F (df, p) | 12 (3, p < 0.001) | 6.08 (3, p = 0.000) | 4.91 (3, p = 0.002) | 4.77 (3, p = 0.003) | 7.75 (3, p < 0.001) | 4.43 (3, p = 0.004) | 3.77 (3, p = 0.010) | 3.65 (3, p = 0.012) |
| **Experiences of co-occuring Common Mental Disorders** |  |  |  |  |  |  |  |  |
| MDD | **1.82 (1.69 - 1.96)*** | - | **1.35 (1.25 - 1.47)*** | **1.21 (1.12 - 1.31)*** | **2.54 (1.95 - 3.30)*** | - | **1.62 (1.38 - 1.91)*** | **1.45 (1.24 - 1.70)*** |
| BP | **1.72 (1.59 - 1.87)*** | - | **1.36 (1.21 - 1.52)*** | **1.27 (1.14 - 1.41)*** | **2.56 (1.94 - 3.39)*** | - | **1.73 (1.36 - 2.19)*** | **1.61 (1.28 - 2.02)*** |
| GAD | **1.72 (1.61 - 1.83)*** | - | 1.06 (0.98 - 1.14) | 1.04 (0.97 - 1.12) | **2.53 (2.14 - 2.98)*** | - | 1.17 (0.99 - 1.39) | 1.16 (0.98 - 1.37) |
| PD | **1.77 (1.64 - 1.90)*** | - | 1.08 (0.99 - 1.18) | 1.09 (1.00 - 1.18) | **2.84 (2.26 - 3.57)*** | - | **1.36 (1.15 - 1.60)*** | **1.36 (1.16 - 1.60)*** |
| PTSD | **2.18 (2.00 - 2.39)*** | - | **1.69 (1.53 - 1.87)*** | **1.35 (1.21 - 1.51)*** | **3.35 (2.76 - 4.06)*** | - | **2.08 (1.71 - 2.54)*** | **1.64 (1.33 - 2.03)*** |
| DUD | **1.24 (1.13 - 1.37)*** | - | 0.99 (0.89 - 1.09) | 0.92 (0.83 - 1.02) | **1.67 (1.38 - 2.02)*** | - | 1.13 (0.95 - 1.35) | 1.04 (0.86 - 1.26) |
| AUD | 1.05 (0.96 - 1.14) | - | 1.03 (0.95 - 1.12) | **0.75 (0.64 - 0.87)*** | 1.12 (0.94 - 1.33) | - | 1.00 (0.85 - 1.17) | **0.69 (0.53 - 0.92)*** |
| ADHD | **1.59 (1.48 - 1.70)*** | - | **1.12 (1.03 - 1.21)*** | **0.80 (0.67 - 0.97)*** | **2.01 (1.65 - 2.45)*** | - | 1.06 (0.92 - 1.22) | 0.71 (0.50 - 1.02) |
| **Number of co-occuring Common Mental Disorders experienced** |  |  |  |  |  |  |  |  |
| 1 Other Disorder | **1.80 (1.62 - 2.00)*** | - | - | **1.54 (1.28 - 1.85)*** | **2.29 (1.85 - 2.83)*** | - | - | **1.61 (1.11 - 2.32)*** |
| 2 Other Disorders | **2.27 (2.04 - 2.53)*** | - | - | **2.18 (1.66 - 2.86)*** | **3.72 (2.98 - 4.64)*** | - | - | **2.45 (1.41 - 4.24)*** |
| 3 or more Other Disorders | **2.84 (2.57 - 3.15)*** | - | - | **2.94 (1.95 - 4.43)*** | **5.29 (3.97 - 7.05)*** | - | - | **3.50 (1.52 - 8.09)*** |
| Number of Disorders experienced: F (df, p) | 162.19 (3, p < 0.001) | - | - | 9.53 (3, p < 0.001) | 55.34 (3, p < 0.001) | - | - | 3.48 (3, p = 0.016) |
| Abbreviations: RR, Risk Ratio, 95% CI, 95% Confidence Interval  Risk ratios and results are from multivariable Poisson distribution and robust standard errors. Data was multiply imputed, m = 20.  All univariable models controlled for year of survey and incentivisation  All multivariable models controlled for all variables included in the model, and for year of survey and incentivisation  Regression models were built hierarchically with socio-demographic variables entered in block 1, co-occurring mental disorders added in block 2, and number of co-occurring mental disorders added in block 3.  * denotes significance difference, p < .05. | | | | | | | | |

Supplementary Table A CTNE: The associations of demographics variables and common mental disorders with lifetime suicide-related outcomes and transition along the suicidal trajectory, RR (95% CI)

|  | Suicide Attempt |  |  |  | Ideation Only |  |  |  |
| --- | --- | --- | --- | --- | --- | --- | --- | --- |
|  | Univariable | Block 1 | Block 2 | Block 3 | Univariable | Block 1 | Block 2 | Block 3 |
| **Age (Ref: 18 & Under)** |  |  |  |  |  |  |  |  |
| 19 | 0.95 (0.65 - 1.38) | 0.95 (0.67 - 1.36) | 0.84 (0.59 - 1.18) | 0.84 (0.60 - 1.18) | 1.00 (0.83 - 1.20) | 1.02 (0.85 - 1.21) | 1.04 (0.88 - 1.24) | 1.04 (0.88 - 1.24) |
| 20 & Older | 1.34 (0.95 - 1.88) | 1.29 (0.91 - 1.84) | 1.08 (0.78 - 1.50) | 1.07 (0.77 - 1.49) | 1.00 (0.83 - 1.21) | 0.99 (0.83 - 1.19) | 1.06 (0.88 - 1.28) | 1.05 (0.87 - 1.27) |
| Age Category: F (df, p) | 1.65 (2, p = 0.194) | 1.17 (2, p = 0.311) | 0.75 (2, p = 0.473) | 0.69 (2, p = 0.501) | 0 (2, p = 0.998) | 0.02 (2, p = 0.979) | 0.25 (2, p = 0.777) | 0.23 (2, p = 0.791) |
| **Gender (Ref: Female)** |  |  |  |  |  |  |  |  |
| Male | **0.56 (0.38 - 0.84)*** | **0.63 (0.42 - 0.94)*** | 0.92 (0.62 - 1.36) | 0.91 (0.62 - 1.35) | 1.09 (0.91 - 1.31) | 1.05 (0.89 - 1.23) | 0.91 (0.78 - 1.06) | 0.91 (0.78 - 1.06) |
| Gender Queer | **2.48 (1.48 - 4.14)*** | 0.59 (0.24 - 1.44) | 0.77 (0.29 - 2.04) | 0.75 (0.29 - 1.94) | **0.31 (0.13 - 0.76)*** | 0.81 (0.21 - 3.16) | 0.75 (0.20 - 2.84) | 0.75 (0.20 - 2.83) |
| Gender: F (df, p) | 11.61 (2, p < 0.001) | 2.85 (2, p = 0.059) | 0.18 (2, p = 0.835) | 0.22 (2, p = 0.805) | 4.41 (2, p = 0.013) | 0.21 (2, p = 0.809) | 0.73 (2, p = 0.482) | 0.71 (2, p = 0.490) |
| **Gender Modality (Ref: Cisgender)** |  |  |  |  |  |  |  |  |
| Transgender | **3.21 (1.98 - 5.20)*** | **2.57 (1.17 - 5.61)*** | 1.50 (0.62 - 3.62) | 1.58 (0.66 - 3.75) | **0.32 (0.16 - 0.68)*** | 0.51 (0.16 - 1.63) | 0.61 (0.20 - 1.90) | 0.61 (0.20 - 1.89) |
| **Sexual Orientation (Ref: Non-heterosexual)** |  |  |  |  |  |  |  |  |
| Non-heterosexual | **2.79 (2.05 - 3.79)*** | **2.31 (1.76 - 3.05)*** | **1.43 (1.08 - 1.88)*** | **1.45 (1.10 - 1.90)*** | **0.64 (0.53 - 0.76)*** | **0.71 (0.59 - 0.85)*** | **0.76 (0.64 - 0.91)*** | **0.76 (0.64 - 0.91)*** |
| **Enrolment Status (Ref: Full time)** |  |  |  |  |  |  |  |  |
| Part-time | 1.45 (0.85 - 2.49) | 1.38 (0.78 - 2.44) | 1.08 (0.68 - 1.73) | 1.11 (0.70 - 1.77) | 1.10 (0.82 - 1.49) | 1.05 (0.79 - 1.40) | 1.05 (0.80 - 1.37) | 1.06 (0.81 - 1.39) |
| **Parental Education (Ref: High School or Less)** |  |  |  |  |  |  |  |  |
| University or higher | 0.93 (0.68 - 1.28) | 0.95 (0.69 - 1.31) | 1.03 (0.76 - 1.41) | 1.03 (0.76 - 1.40) | 1.01 (0.86 - 1.18) | 1.02 (0.87 - 1.19) | 1.00 (0.86 - 1.16) | 1.00 (0.86 - 1.16) |
| **Ethnicity (Ref: Pākehā)** |  |  |  |  |  |  |  |  |
| Māori | 1.51 (0.98 - 2.33) | **1.56 (1.02 - 2.38)*** | 1.16 (0.78 - 1.72) | 1.15 (0.78 - 1.72) | 0.77 (0.60 - 1.00) | **0.78 (0.61 - 0.99)*** | 0.87 (0.68 - 1.11) | 0.87 (0.68 - 1.11) |
| Pacific Peoples | 1.25 (0.63 - 2.48) | 1.41 (0.74 - 2.69) | 1.22 (0.69 - 2.18) | 1.28 (0.72 - 2.27) | 0.95 (0.67 - 1.33) | 0.88 (0.63 - 1.22) | 0.92 (0.67 - 1.27) | 0.92 (0.67 - 1.27) |
| Asian | 0.90 (0.57 - 1.41) | 0.98 (0.62 - 1.55) | 1.22 (0.78 - 1.91) | 1.27 (0.81 - 2.00) | 1.15 (0.95 - 1.40) | 1.10 (0.91 - 1.32) | 0.98 (0.81 - 1.18) | 0.99 (0.82 - 1.20) |
| MELAA | 1.17 (0.52 - 2.66) | 1.29 (0.61 - 2.72) | 1.32 (0.65 - 2.67) | 1.41 (0.71 - 2.80) | 0.79 (0.45 - 1.37) | 0.77 (0.47 - 1.28) | 0.83 (0.50 - 1.39) | 0.85 (0.51 - 1.41) |
| Ethnicity: F (df, p) | 1.06 (4, p = 0.376) | 1.12 (4, p = 0.345) | 0.37 (4, p = 0.831) | 0.54 (4, p = 0.703) | 2.14 (4, p = 0.074) | 1.87 (4, p = 0.113) | 0.44 (4, p = 0.777) | 0.44 (4, p = 0.777) |
| **Course (Ref: Arts & Sciences)** |  |  |  |  |  |  |  |  |
| Medical Sciences | 0.67 (0.38 - 1.16) | 0.77 (0.44 - 1.33) | 0.76 (0.45 - 1.28) | 0.77 (0.45 - 1.30) | 1.19 (0.97 - 1.47) | 1.19 (0.96 - 1.48) | 1.19 (0.97 - 1.47) | 1.19 (0.97 - 1.47) |
| Commerce | 1.27 (0.90 - 1.81) | 1.17 (0.83 - 1.64) | 1.08 (0.77 - 1.50) | 1.08 (0.78 - 1.49) | 0.86 (0.70 - 1.06) | 0.88 (0.73 - 1.07) | 0.90 (0.75 - 1.09) | 0.90 (0.75 - 1.09) |
| Laws | **1.84 (1.06 - 3.20)*** | 1.42 (0.84 - 2.40) | 1.29 (0.79 - 2.13) | 1.31 (0.80 - 2.15) | 0.81 (0.55 - 1.19) | 0.90 (0.61 - 1.31) | 0.89 (0.62 - 1.28) | 0.89 (0.62 - 1.28) |
| Course: F (df, p) | 3.03 (3, p = 0.029) | 1.23 (3, p = 0.297) | 0.88 (3, p = 0.450) | 0.91 (3, p = 0.436) | 2.33 (3, p = 0.073) | 1.82 (3, p = 0.142) | 1.83 (3, p = 0.141) | 1.86 (3, p = 0.136) |
| **Experiences of co-occuring Common Mental Disorders** |  |  |  |  |  |  |  |  |
| MDD | **3.11 (1.97 - 4.92)*** | - | **2.00 (1.38 - 2.91)*** | **1.80 (1.23 - 2.64)*** | **0.66 (0.56 - 0.78)*** | - | **0.81 (0.69 - 0.96)*** | **0.78 (0.65 - 0.94)*** |
| BP | **5.84 (3.62 - 9.42)*** | - | **3.31 (2.14 - 5.12)*** | **3.07 (1.99 - 4.73)*** | **0.34 (0.18 - 0.63)*** | - | **0.46 (0.26 - 0.83)*** | **0.46 (0.25 - 0.81)*** |
| GAD | **3.59 (2.72 - 4.73)*** | - | 1.16 (0.84 - 1.59) | 1.15 (0.85 - 1.57) | **0.55 (0.44 - 0.70)*** | - | 0.84 (0.65 - 1.09) | 0.84 (0.65 - 1.08) |
| PD | **4.10 (2.74 - 6.13)*** | - | 1.33 (0.93 - 1.89) | 1.33 (0.94 - 1.89) | **0.43 (0.32 - 0.59)*** | - | **0.61 (0.45 - 0.83)*** | **0.61 (0.45 - 0.83)*** |
| PTSD | **9.02 (5.83 - 13.94)*** | - | **4.26 (2.62 - 6.93)*** | **3.03 (1.77 - 5.20)*** | **0.60 (0.50 - 0.73)*** | - | **0.79 (0.68 - 0.91)*** | **0.73 (0.59 - 0.90)*** |
| DUD | **3.05 (2.23 - 4.19)*** | - | **1.52 (1.07 - 2.14)*** | 1.31 (0.94 - 1.83) | **0.63 (0.48 - 0.82)*** | - | 0.81 (0.61 - 1.08) | 0.79 (0.59 - 1.06) |
| AUD | **1.59 (1.15 - 2.19)*** | - | 1.25 (0.89 - 1.75) | 0.74 (0.43 - 1.27) | 0.88 (0.70 - 1.09) | - | 0.99 (0.83 - 1.19) | 0.89 (0.67 - 1.20) |
| ADHD | **3.07 (2.18 - 4.32)*** | - | 1.22 (0.88 - 1.69) | 0.65 (0.32 - 1.31) | **0.70 (0.55 - 0.88)*** | - | 1.00 (0.81 - 1.23) | 0.90 (0.64 - 1.27) |
| **Number of co-occuring Common Mental Disorders experienced** |  |  |  |  |  |  |  |  |
| 1 Other Disorder | **4.43 (2.66 - 7.39)*** | - | - | 2.20 (0.72 - 6.69) | **0.79 (0.67 - 0.93)*** | - | - | 1.16 (0.89 - 1.51) |
| 2 Other Disorders | **10.11 (6.19 - 16.51)*** | - | - | **4.23 (1.07 - 16.77)*** | **0.54 (0.42 - 0.68)*** | - | - | 1.29 (0.79 - 2.10) |
| 3 or more Other Disorders | **20.32 (11.55 - 35.76)*** | - | - | **7.88 (1.29 - 48.28)*** | **0.33 (0.20 - 0.55)*** | - | - | 1.40 (0.62 - 3.15) |
| Number of Disorders experienced: F (df, p) | 44.26 (3, p < 0.001) | - | - | 2.02 (3, p = 0.110) | 13.51 (3, p < 0.001) | - | - | 0.36 (3, p = 0.785) |
| Abbreviations: RR, Risk Ratio, 95% CI, 95% Confidence Interval  Risk ratios and results are from multivariable Poisson distribution and robust standard errors. Data was multiply imputed, m = 20.  All univariable models controlled for year of survey and incentivisation  All multivariable models controlled for all variables included in the model, and for year of survey and incentivisation  Regression models were built hierarchically with socio-demographic variables entered in block 1, co-occurring mental disorders added in block 2, and number of co-occurring mental disorders added in block 3.  * denotes significance difference, p < .05. | | | | | | | | |

Supplementary Table A CTNE: The associations of demographics variables and common mental disorders with lifetime suicide-related outcomes and transition along the suicidal trajectory, RR (95% CI)

|  | Plan No Attempt |  |  |  | Attempt If Plan |  |  |  |
| --- | --- | --- | --- | --- | --- | --- | --- | --- |
|  | Univariable | Block 1 | Block 2 | Block 3 | Univariable | Block 1 | Block 2 | Block 3 |
| **Age (Ref: 18 & Under)** |  |  |  |  |  |  |  |  |
| 19 | 1.01 (0.88 - 1.17) | 1.02 (0.89 - 1.17) | 1.04 (0.92 - 1.19) | 1.04 (0.92 - 1.19) | 0.96 (0.65 - 1.40) | 0.95 (0.66 - 1.37) | 0.89 (0.62 - 1.28) | 0.90 (0.62 - 1.28) |
| 20 & Older | 0.90 (0.76 - 1.07) | 0.90 (0.76 - 1.06) | 0.94 (0.79 - 1.11) | 0.94 (0.80 - 1.11) | 1.24 (0.90 - 1.72) | 1.25 (0.90 - 1.73) | 1.14 (0.82 - 1.60) | 1.13 (0.81 - 1.59) |
| Age Category: F (df, p) | 0.78 (2, p = 0.460) | 0.85 (2, p = 0.429) | 0.56 (2, p = 0.572) | 0.53 (2, p = 0.592) | 0.94 (2, p = 0.391) | 1.02 (2, p = 0.361) | 0.59 (2, p = 0.555) | 0.53 (2, p = 0.589) |
| **Gender (Ref: Female)** |  |  |  |  |  |  |  |  |
| Male | 1.10 (0.96 - 1.27) | 1.11 (0.97 - 1.28) | 1.05 (0.92 - 1.20) | 1.05 (0.92 - 1.20) | 0.77 (0.51 - 1.16) | 0.75 (0.50 - 1.13) | 0.85 (0.58 - 1.27) | 0.85 (0.57 - 1.26) |
| Gender Queer | 0.93 (0.71 - 1.23) | 1.35 (0.67 - 2.70) | 1.22 (0.59 - 2.50) | 1.22 (0.59 - 2.51) | 1.13 (0.67 - 1.89) | 0.59 (0.24 - 1.45) | 0.72 (0.28 - 1.87) | 0.70 (0.27 - 1.81) |
| Gender: F (df, p) | 1.19 (2, p = 0.307) | 1.45 (2, p = 0.237) | 0.40 (2, p = 0.668) | 0.34 (2, p = 0.712) | 0.97 (2, p = 0.380) | 1.30 (2, p = 0.273) | 0.43 (2, p = 0.648) | 0.48 (2, p = 0.618) |
| **Gender Modality (Ref: Cisgender)** |  |  |  |  |  |  |  |  |
| Transgender | 0.86 (0.66 - 1.11) | 0.71 (0.37 - 1.34) | 0.80 (0.40 - 1.59) | 0.79 (0.40 - 1.58) | 1.34 (0.88 - 2.06) | 1.82 (0.85 - 3.93) | 1.42 (0.60 - 3.36) | 1.46 (0.62 - 3.43) |
| **Sexual Orientation (Ref: Non-heterosexual)** |  |  |  |  |  |  |  |  |
| Non-heterosexual | 0.92 (0.83 - 1.03) | 0.96 (0.86 - 1.07) | 0.99 (0.89 - 1.10) | 0.99 (0.89 - 1.11) | 1.21 (0.94 - 1.57) | 1.11 (0.85 - 1.45) | 1.01 (0.78 - 1.29) | 1.02 (0.79 - 1.31) |
| **Enrolment Status (Ref: Full time)** |  |  |  |  |  |  |  |  |
| Part-time | 0.90 (0.67 - 1.21) | 0.93 (0.68 - 1.26) | 0.97 (0.73 - 1.29) | 0.97 (0.73 - 1.28) | 1.23 (0.72 - 2.10) | 1.14 (0.66 - 1.98) | 0.99 (0.60 - 1.64) | 1.02 (0.62 - 1.68) |
| **Parental Education (Ref: High School or Less)** |  |  |  |  |  |  |  |  |
| University or higher | 1.03 (0.90 - 1.17) | 1.01 (0.89 - 1.15) | 0.98 (0.86 - 1.11) | 0.98 (0.87 - 1.11) | 0.94 (0.69 - 1.27) | 0.97 (0.72 - 1.32) | 1.03 (0.77 - 1.39) | 1.03 (0.77 - 1.38) |
| **Ethnicity (Ref: Pākehā)** |  |  |  |  |  |  |  |  |
| Māori | 0.89 (0.73 - 1.09) | 0.89 (0.73 - 1.08) | 0.96 (0.80 - 1.17) | 0.96 (0.80 - 1.17) | 1.28 (0.82 - 1.98) | 1.30 (0.85 - 1.97) | 1.08 (0.73 - 1.59) | 1.09 (0.74 - 1.60) |
| Pacific Peoples | 0.90 (0.65 - 1.26) | 0.91 (0.66 - 1.25) | 0.91 (0.68 - 1.22) | 0.90 (0.67 - 1.21) | 1.23 (0.63 - 2.40) | 1.23 (0.63 - 2.37) | 1.12 (0.62 - 2.05) | 1.18 (0.65 - 2.13) |
| Asian | 1.00 (0.85 - 1.18) | 0.99 (0.84 - 1.17) | 0.92 (0.78 - 1.09) | 0.93 (0.78 - 1.10) | 0.99 (0.65 - 1.50) | 1.01 (0.67 - 1.54) | 1.21 (0.80 - 1.84) | 1.26 (0.83 - 1.94) |
| MELAA | 0.98 (0.70 - 1.38) | 0.95 (0.68 - 1.31) | 0.94 (0.71 - 1.27) | 0.95 (0.70 - 1.27) | 1.02 (0.44 - 2.35) | 1.10 (0.51 - 2.40) | 1.13 (0.55 - 2.30) | 1.19 (0.59 - 2.41) |
| Ethnicity: F (df, p) | 0.36 (4, p = 0.834) | 0.38 (4, p = 0.822) | 0.30 (4, p = 0.878) | 0.28 (4, p = 0.888) | 0.37 (4, p = 0.834) | 0.39 (4, p = 0.818) | 0.24 (4, p = 0.918) | 0.37 (4, p = 0.833) |
| **Course (Ref: Arts & Sciences)** |  |  |  |  |  |  |  |  |
| Medical Sciences | 1.03 (0.84 - 1.26) | 1.00 (0.82 - 1.22) | 1.01 (0.83 - 1.23) | 1.00 (0.82 - 1.22) | 0.93 (0.56 - 1.55) | 0.99 (0.59 - 1.64) | 0.93 (0.57 - 1.53) | 0.96 (0.58 - 1.57) |
| Commerce | 1.02 (0.89 - 1.17) | 1.01 (0.88 - 1.16) | 0.98 (0.86 - 1.12) | 0.98 (0.86 - 1.12) | 0.96 (0.68 - 1.35) | 0.96 (0.69 - 1.34) | 1.02 (0.74 - 1.40) | 1.02 (0.74 - 1.40) |
| Laws | 0.90 (0.67 - 1.22) | 0.93 (0.70 - 1.23) | 0.94 (0.73 - 1.22) | 0.94 (0.73 - 1.23) | 1.21 (0.71 - 2.07) | 1.15 (0.67 - 1.98) | 1.08 (0.66 - 1.78) | 1.11 (0.68 - 1.84) |
| Course: F (df, p) | 0.22 (3, p = 0.884) | 0.11 (3, p = 0.954) | 0.10 (3, p = 0.962) | 0.09 (3, p = 0.966) | 0.26 (3, p = 0.855) | 0.13 (3, p = 0.943) | 0.08 (3, p = 0.972) | 0.09 (3, p = 0.967) |
| **Experiences of co-occuring Common Mental Disorders** |  |  |  |  |  |  |  |  |
| MDD | 0.90 (0.79 - 1.02) | - | 0.93 (0.82 - 1.05) | 0.93 (0.79 - 1.09) | 1.30 (0.93 - 1.83) | - | 1.35 (0.93 - 1.94) | 1.27 (0.86 - 1.87) |
| BP | **0.54 (0.32 - 0.92)*** | - | **0.62 (0.43 - 0.90)*** | **0.63 (0.44 - 0.90)*** | **2.26 (1.57 - 3.24)*** | - | **1.96 (1.29 - 2.99)*** | **1.89 (1.23 - 2.88)*** |
| GAD | **0.82 (0.72 - 0.94)*** | - | 0.98 (0.83 - 1.15) | 0.98 (0.83 - 1.14) | **1.56 (1.17 - 2.06)*** | - | 1.04 (0.75 - 1.44) | 1.03 (0.75 - 1.43) |
| PD | **0.80 (0.69 - 0.94)*** | - | 0.96 (0.81 - 1.12) | 0.96 (0.81 - 1.13) | **1.60 (1.16 - 2.19)*** | - | 1.07 (0.78 - 1.46) | 1.06 (0.78 - 1.45) |
| PTSD | **0.72 (0.65 - 0.80)*** | - | **0.82 (0.74 - 0.91)*** | **0.82 (0.71 - 0.96)*** | **3.19 (1.90 - 5.34)*** | - | **2.32 (1.41 - 3.83)*** | **1.98 (1.14 - 3.45)*** |
| DUD | **0.66 (0.53 - 0.81)*** | - | **0.76 (0.62 - 0.94)*** | **0.78 (0.63 - 0.96)*** | **2.05 (1.56 - 2.69)*** | - | **1.51 (1.10 - 2.06)*** | 1.35 (0.98 - 1.86) |
| AUD | **0.86 (0.76 - 0.99)*** | - | 0.93 (0.81 - 1.07) | 0.99 (0.76 - 1.27) | **1.42 (1.03 - 1.95)*** | - | 1.19 (0.83 - 1.69) | 0.82 (0.48 - 1.40) |
| ADHD | **0.79 (0.67 - 0.93)*** | - | 0.92 (0.78 - 1.09) | 1.01 (0.76 - 1.33) | **1.60 (1.21 - 2.13)*** | - | 1.14 (0.84 - 1.55) | 0.74 (0.38 - 1.45) |
| **Number of co-occuring Common Mental Disorders experienced** |  |  |  |  |  |  |  |  |
| 1 Other Disorder | **0.84 (0.75 - 0.94)*** | - | - | 1.05 (0.86 - 1.30) | **2.15 (1.22 - 3.81)*** | - | - | 1.40 (0.40 - 4.88) |
| 2 Other Disorders | **0.69 (0.60 - 0.80)*** | - | - | 0.98 (0.64 - 1.49) | **3.29 (1.93 - 5.60)*** | - | - | 2.35 (0.54 - 10.22) |
| 3 or more Other Disorders | **0.55 (0.41 - 0.73)*** | - | - | 0.86 (0.44 - 1.69) | **4.35 (2.50 - 7.58)*** | - | - | 3.47 (0.56 - 21.52) |
| Number of Disorders experienced: F (df, p) | 11.97 (3, p < 0.001) | - | - | 0.71 (3, p = 0.544) | 10.44 (3, p < 0.001) | - | - | 0.96 (3, p = 0.412) |
| Abbreviations: RR, Risk Ratio, 95% CI, 95% Confidence Interval  Risk ratios and results are from multivariable Poisson distribution and robust standard errors. Data was multiply imputed, m = 20.  All univariable models controlled for year of survey and incentivisation  All multivariable models controlled for all variables included in the model, and for year of survey and incentivisation  Regression models were built hierarchically with socio-demographic variables entered in block 1, co-occurring mental disorders added in block 2, and number of co-occurring mental disorders added in block 3.  * denotes significance difference, p < .05. | | | | | | | | |

Supplementary Table A CTNE: The associations of demographics variables and common mental disorders with lifetime suicide-related outcomes and transition along the suicidal trajectory, RR (95% CI)

|  | Plan No Attempt |  |  |  | Attempt If Plan |  |  |  |
| --- | --- | --- | --- | --- | --- | --- | --- | --- |
|  | Univariable | Block 1 | Block 2 | Block 3 | Univariable | Block 1 | Block 2 | Block 3 |
| **Age (Ref: 18 & Under)** |  |  |  |  |  |  |  |  |
| 19 | 1.01 (0.88 - 1.17) | 1.02 (0.89 - 1.17) | 1.04 (0.92 - 1.19) | 1.04 (0.92 - 1.19) | 0.96 (0.65 - 1.40) | 0.95 (0.66 - 1.37) | 0.89 (0.62 - 1.28) | 0.90 (0.62 - 1.28) |
| 20 & Older | 0.90 (0.76 - 1.07) | 0.90 (0.76 - 1.06) | 0.94 (0.79 - 1.11) | 0.94 (0.80 - 1.11) | 1.24 (0.90 - 1.72) | 1.25 (0.90 - 1.73) | 1.14 (0.82 - 1.60) | 1.13 (0.81 - 1.59) |
| Age Category: F (df, p) | 0.78 (2, p = 0.460) | 0.85 (2, p = 0.429) | 0.56 (2, p = 0.572) | 0.53 (2, p = 0.592) | 0.94 (2, p = 0.391) | 1.02 (2, p = 0.361) | 0.59 (2, p = 0.555) | 0.53 (2, p = 0.589) |
| **Gender (Ref: Female)** |  |  |  |  |  |  |  |  |
| Male | 1.10 (0.96 - 1.27) | 1.11 (0.97 - 1.28) | 1.05 (0.92 - 1.20) | 1.05 (0.92 - 1.20) | 0.77 (0.51 - 1.16) | 0.75 (0.50 - 1.13) | 0.85 (0.58 - 1.27) | 0.85 (0.57 - 1.26) |
| Gender Queer | 0.93 (0.71 - 1.23) | 1.35 (0.67 - 2.70) | 1.22 (0.59 - 2.50) | 1.22 (0.59 - 2.51) | 1.13 (0.67 - 1.89) | 0.59 (0.24 - 1.45) | 0.72 (0.28 - 1.87) | 0.70 (0.27 - 1.81) |
| Gender: F (df, p) | 1.19 (2, p = 0.307) | 1.45 (2, p = 0.237) | 0.40 (2, p = 0.668) | 0.34 (2, p = 0.712) | 0.97 (2, p = 0.380) | 1.30 (2, p = 0.273) | 0.43 (2, p = 0.648) | 0.48 (2, p = 0.618) |
| **Gender Modality (Ref: Cisgender)** |  |  |  |  |  |  |  |  |
| Transgender | 0.86 (0.66 - 1.11) | 0.71 (0.37 - 1.34) | 0.80 (0.40 - 1.59) | 0.79 (0.40 - 1.58) | 1.34 (0.88 - 2.06) | 1.82 (0.85 - 3.93) | 1.42 (0.60 - 3.36) | 1.46 (0.62 - 3.43) |
| **Sexual Orientation (Ref: Non-heterosexual)** |  |  |  |  |  |  |  |  |
| Non-heterosexual | 0.92 (0.83 - 1.03) | 0.96 (0.86 - 1.07) | 0.99 (0.89 - 1.10) | 0.99 (0.89 - 1.11) | 1.21 (0.94 - 1.57) | 1.11 (0.85 - 1.45) | 1.01 (0.78 - 1.29) | 1.02 (0.79 - 1.31) |
| **Enrolment Status (Ref: Full time)** |  |  |  |  |  |  |  |  |
| Part-time | 0.90 (0.67 - 1.21) | 0.93 (0.68 - 1.26) | 0.97 (0.73 - 1.29) | 0.97 (0.73 - 1.28) | 1.23 (0.72 - 2.10) | 1.14 (0.66 - 1.98) | 0.99 (0.60 - 1.64) | 1.02 (0.62 - 1.68) |
| **Parental Education (Ref: High School or Less)** |  |  |  |  |  |  |  |  |
| University or higher | 1.03 (0.90 - 1.17) | 1.01 (0.89 - 1.15) | 0.98 (0.86 - 1.11) | 0.98 (0.87 - 1.11) | 0.94 (0.69 - 1.27) | 0.97 (0.72 - 1.32) | 1.03 (0.77 - 1.39) | 1.03 (0.77 - 1.38) |
| **Ethnicity (Ref: Pākehā)** |  |  |  |  |  |  |  |  |
| Māori | 0.89 (0.73 - 1.09) | 0.89 (0.73 - 1.08) | 0.96 (0.80 - 1.17) | 0.96 (0.80 - 1.17) | 1.28 (0.82 - 1.98) | 1.30 (0.85 - 1.97) | 1.08 (0.73 - 1.59) | 1.09 (0.74 - 1.60) |
| Pacific Peoples | 0.90 (0.65 - 1.26) | 0.91 (0.66 - 1.25) | 0.91 (0.68 - 1.22) | 0.90 (0.67 - 1.21) | 1.23 (0.63 - 2.40) | 1.23 (0.63 - 2.37) | 1.12 (0.62 - 2.05) | 1.18 (0.65 - 2.13) |
| Asian | 1.00 (0.85 - 1.18) | 0.99 (0.84 - 1.17) | 0.92 (0.78 - 1.09) | 0.93 (0.78 - 1.10) | 0.99 (0.65 - 1.50) | 1.01 (0.67 - 1.54) | 1.21 (0.80 - 1.84) | 1.26 (0.83 - 1.94) |
| MELAA | 0.98 (0.70 - 1.38) | 0.95 (0.68 - 1.31) | 0.94 (0.71 - 1.27) | 0.95 (0.70 - 1.27) | 1.02 (0.44 - 2.35) | 1.10 (0.51 - 2.40) | 1.13 (0.55 - 2.30) | 1.19 (0.59 - 2.41) |
| Ethnicity: F (df, p) | 0.36 (4, p = 0.834) | 0.38 (4, p = 0.822) | 0.30 (4, p = 0.878) | 0.28 (4, p = 0.888) | 0.37 (4, p = 0.834) | 0.39 (4, p = 0.818) | 0.24 (4, p = 0.918) | 0.37 (4, p = 0.833) |
| **Course (Ref: Arts & Sciences)** |  |  |  |  |  |  |  |  |
| Medical Sciences | 1.03 (0.84 - 1.26) | 1.00 (0.82 - 1.22) | 1.01 (0.83 - 1.23) | 1.00 (0.82 - 1.22) | 0.93 (0.56 - 1.55) | 0.99 (0.59 - 1.64) | 0.93 (0.57 - 1.53) | 0.96 (0.58 - 1.57) |
| Commerce | 1.02 (0.89 - 1.17) | 1.01 (0.88 - 1.16) | 0.98 (0.86 - 1.12) | 0.98 (0.86 - 1.12) | 0.96 (0.68 - 1.35) | 0.96 (0.69 - 1.34) | 1.02 (0.74 - 1.40) | 1.02 (0.74 - 1.40) |
| Laws | 0.90 (0.67 - 1.22) | 0.93 (0.70 - 1.23) | 0.94 (0.73 - 1.22) | 0.94 (0.73 - 1.23) | 1.21 (0.71 - 2.07) | 1.15 (0.67 - 1.98) | 1.08 (0.66 - 1.78) | 1.11 (0.68 - 1.84) |
| Course: F (df, p) | 0.22 (3, p = 0.884) | 0.11 (3, p = 0.954) | 0.10 (3, p = 0.962) | 0.09 (3, p = 0.966) | 0.26 (3, p = 0.855) | 0.13 (3, p = 0.943) | 0.08 (3, p = 0.972) | 0.09 (3, p = 0.967) |
| **Experiences of co-occuring Common Mental Disorders** |  |  |  |  |  |  |  |  |
| MDD | 0.90 (0.79 - 1.02) | - | 0.93 (0.82 - 1.05) | 0.93 (0.79 - 1.09) | 1.30 (0.93 - 1.83) | - | 1.35 (0.93 - 1.94) | 1.27 (0.86 - 1.87) |
| BP | **0.54 (0.32 - 0.92)*** | - | **0.62 (0.43 - 0.90)*** | **0.63 (0.44 - 0.90)*** | **2.26 (1.57 - 3.24)*** | - | **1.96 (1.29 - 2.99)*** | **1.89 (1.23 - 2.88)*** |
| GAD | **0.82 (0.72 - 0.94)*** | - | 0.98 (0.83 - 1.15) | 0.98 (0.83 - 1.14) | **1.56 (1.17 - 2.06)*** | - | 1.04 (0.75 - 1.44) | 1.03 (0.75 - 1.43) |
| PD | **0.80 (0.69 - 0.94)*** | - | 0.96 (0.81 - 1.12) | 0.96 (0.81 - 1.13) | **1.60 (1.16 - 2.19)*** | - | 1.07 (0.78 - 1.46) | 1.06 (0.78 - 1.45) |
| PTSD | **0.72 (0.65 - 0.80)*** | - | **0.82 (0.74 - 0.91)*** | **0.82 (0.71 - 0.96)*** | **3.19 (1.90 - 5.34)*** | - | **2.32 (1.41 - 3.83)*** | **1.98 (1.14 - 3.45)*** |
| DUD | **0.66 (0.53 - 0.81)*** | - | **0.76 (0.62 - 0.94)*** | **0.78 (0.63 - 0.96)*** | **2.05 (1.56 - 2.69)*** | - | **1.51 (1.10 - 2.06)*** | 1.35 (0.98 - 1.86) |
| AUD | **0.86 (0.76 - 0.99)*** | - | 0.93 (0.81 - 1.07) | 0.99 (0.76 - 1.27) | **1.42 (1.03 - 1.95)*** | - | 1.19 (0.83 - 1.69) | 0.82 (0.48 - 1.40) |
| ADHD | **0.79 (0.67 - 0.93)*** | - | 0.92 (0.78 - 1.09) | 1.01 (0.76 - 1.33) | **1.60 (1.21 - 2.13)*** | - | 1.14 (0.84 - 1.55) | 0.74 (0.38 - 1.45) |
| **Number of co-occuring Common Mental Disorders experienced** |  |  |  |  |  |  |  |  |
| 1 Other Disorder | **0.84 (0.75 - 0.94)*** | - | - | 1.05 (0.86 - 1.30) | **2.15 (1.22 - 3.81)*** | - | - | 1.40 (0.40 - 4.88) |
| 2 Other Disorders | **0.69 (0.60 - 0.80)*** | - | - | 0.98 (0.64 - 1.49) | **3.29 (1.93 - 5.60)*** | - | - | 2.35 (0.54 - 10.22) |
| 3 or more Other Disorders | **0.55 (0.41 - 0.73)*** | - | - | 0.86 (0.44 - 1.69) | **4.35 (2.50 - 7.58)*** | - | - | 3.47 (0.56 - 21.52) |
| Number of Disorders experienced: F (df, p) | 11.97 (3, p < 0.001) | - | - | 0.71 (3, p = 0.544) | 10.44 (3, p < 0.001) | - | - | 0.96 (3, p = 0.412) |
| Abbreviations: RR, Risk Ratio, 95% CI, 95% Confidence Interval  Risk ratios and results are from multivariable Poisson distribution and robust standard errors. Data was multiply imputed, m = 20.  All univariable models controlled for year of survey and incentivisation  All multivariable models controlled for all variables included in the model, and for year of survey and incentivisation  Regression models were built hierarchically with socio-demographic variables entered in block 1, co-occurring mental disorders added in block 2, and number of co-occurring mental disorders added in block 3.  * denotes significance difference, p < .05. | | | | | | | | |

Supplementary Table B: The associations of demographics variables and common mental disorders with 12-month suicide-related outcomes and transition along the suicidal trajectory, RR (95% CI)

|  | Suicidal Ideation | | | | Suicide Plan | | | |
| --- | --- | --- | --- | --- | --- | --- | --- | --- |
|  | Univariable | Block 1 | Block 2 | Block 3 | Univariable | Block 1 | Block 2 | Block 3 |
| **Age (Ref: 18 & Under)** |  |  |  |  |  |  |  |  |
| 19 | 0.93 (0.80 - 1.08) | 0.94 (0.81 - 1.09) | 0.92 (0.80 - 1.06) | 0.91 (0.79 - 1.04) | 0.96 (0.73 - 1.25) | 0.97 (0.76 - 1.24) | 0.91 (0.72 - 1.15) | 0.90 (0.72 - 1.14) |
| 20 & Older | 0.93 (0.81 - 1.08) | 0.93 (0.80 - 1.08) | 0.91 (0.79 - 1.04) | 0.89 (0.78 - 1.03) | 0.97 (0.75 - 1.25) | 0.97 (0.76 - 1.24) | 0.90 (0.71 - 1.13) | 0.87 (0.69 - 1.10) |
| Age Category: F (df, p) | 0.77 (2, p = 0.465) | 0.68 (2, p = 0.508) | 1.46 (2, p = 0.233) | 1.91 (2, p = 0.148) | 0.08 (2, p = 0.928) | 0.05 (2, p = 0.949) | 0.61 (2, p = 0.543) | 0.90 (2, p = 0.408) |
| **Gender (Ref: Female)** |  |  |  |  |  |  |  |  |
| Male | **0.73 (0.64 - 0.84)*** | **0.81 (0.71 - 0.93)*** | 1.00 (0.87 - 1.14) | 1.01 (0.88 - 1.15) | **0.67 (0.51 - 0.87)*** | **0.73 (0.57 - 0.95)*** | 1.04 (0.81 - 1.33) | 1.05 (0.82 - 1.35) |
| Gender Queer | **1.85 (1.55 - 2.20)*** | 0.84 (0.61 - 1.17) | 0.98 (0.69 - 1.38) | 0.99 (0.72 - 1.37) | **2.59 (1.88 - 3.58)*** | 0.70 (0.43 - 1.12) | 0.85 (0.52 - 1.40) | 0.87 (0.54 - 1.40) |
| Gender: F (df, p) | 41.5 (2, p < 0.001) | 4.18 (2, p = 0.015) | 0.01 (2, p = 0.991) | 0.01 (2, p = 0.992) | 25.17 (2, p < 0.001) | 3.18 (2, p = 0.042) | 0.34 (2, p = 0.712) | 0.36 (2, p = 0.695) |
| **Gender Modality (Ref: Cisgender)** |  |  |  |  |  |  |  |  |
| Transgender | **2.08 (1.78 - 2.42)*** | **1.47 (1.10 - 1.96)*** | 1.10 (0.82 - 1.49) | 1.09 (0.83 - 1.44) | **3.18 (2.35 - 4.29)*** | **2.17 (1.42 - 3.31)*** | 1.46 (0.94 - 2.26) | 1.45 (0.95 - 2.20) |
| **Sexual Orientation (Ref: Non-heterosexual)** |  |  |  |  |  |  |  |  |
| Non-heterosexual | **2.08 (1.86 - 2.32)*** | **1.90 (1.70 - 2.11)*** | **1.50 (1.35 - 1.67)*** | **1.50 (1.35 - 1.67)*** | **2.87 (2.31 - 3.58)*** | **2.42 (1.97 - 2.96)*** | **1.72 (1.39 - 2.12)*** | **1.72 (1.40 - 2.11)*** |
| **Enrolment Status (Ref: Full time)** |  |  |  |  |  |  |  |  |
| Part-time | 0.97 (0.75 - 1.25) | 1.04 (0.81 - 1.34) | 0.95 (0.75 - 1.21) | 0.97 (0.76 - 1.23) | 0.95 (0.60 - 1.50) | 1.05 (0.67 - 1.64) | 0.89 (0.59 - 1.34) | 0.92 (0.61 - 1.37) |
| **Parental Education (Ref: High School or Less)** |  |  |  |  |  |  |  |  |
| University or higher | 0.98 (0.87 - 1.11) | 0.95 (0.85 - 1.07) | 0.99 (0.89 - 1.11) | 0.99 (0.89 - 1.11) | 0.99 (0.80 - 1.22) | 0.95 (0.78 - 1.15) | 1.02 (0.85 - 1.23) | 1.02 (0.85 - 1.23) |
| **Ethnicity (Ref: Pākehā)** |  |  |  |  |  |  |  |  |
| Māori | 1.05 (0.87 - 1.25) | 1.06 (0.89 - 1.25) | 0.92 (0.78 - 1.08) | 0.92 (0.79 - 1.08) | 1.33 (0.97 - 1.82) | **1.37 (1.03 - 1.82)*** | 1.07 (0.82 - 1.40) | 1.07 (0.82 - 1.40) |
| Pacific Peoples | 1.03 (0.80 - 1.34) | 1.10 (0.87 - 1.40) | 1.00 (0.80 - 1.26) | 1.02 (0.82 - 1.28) | 1.12 (0.68 - 1.85) | 1.27 (0.82 - 1.96) | 1.05 (0.70 - 1.58) | 1.08 (0.72 - 1.61) |
| Asian | 0.97 (0.83 - 1.15) | 1.09 (0.94 - 1.28) | 1.14 (0.97 - 1.33) | 1.18 (1.00 - 1.38) | 0.92 (0.69 - 1.23) | 1.09 (0.84 - 1.43) | 1.11 (0.86 - 1.44) | 1.16 (0.89 - 1.51) |
| MELAA | 1.00 (0.73 - 1.38) | 1.08 (0.79 - 1.47) | 0.98 (0.76 - 1.26) | 1.01 (0.78 - 1.30) | 1.29 (0.73 - 2.28) | 1.43 (0.83 - 2.45) | 1.17 (0.72 - 1.89) | 1.21 (0.74 - 1.97) |
| Ethnicity: F (df, p) | 0.12 (4, p = 0.976) | 0.46 (4, p = 0.765) | 1.14 (4, p = 0.334) | 1.53 (4, p = 0.190) | 1.16 (4, p = 0.327) | 1.52 (4, p = 0.195) | 0.26 (4, p = 0.903) | 0.42 (4, p = 0.793) |
| **Course (Ref: Arts & Sciences)** |  |  |  |  |  |  |  |  |
| Medical Sciences | **0.78 (0.64 - 0.96)*** | 0.82 (0.68 - 1.00) | **0.81 (0.68 - 0.97)*** | **0.83 (0.69 - 0.99)*** | 0.70 (0.49 - 1.01) | 0.75 (0.53 - 1.05) | 0.73 (0.53 - 1.01) | 0.75 (0.54 - 1.04) |
| Commerce | **1.21 (1.06 - 1.38)*** | **1.17 (1.03 - 1.33)*** | 1.12 (0.98 - 1.27) | 1.11 (0.98 - 1.26) | **1.39 (1.08 - 1.78)*** | **1.31 (1.05 - 1.62)*** | 1.21 (0.98 - 1.49) | 1.20 (0.98 - 1.48) |
| Laws | **1.34 (1.09 - 1.66)*** | 1.16 (0.94 - 1.44) | 1.13 (0.92 - 1.39) | 1.13 (0.92 - 1.39) | **1.60 (1.10 - 2.32)*** | 1.31 (0.94 - 1.84) | 1.25 (0.89 - 1.74) | 1.27 (0.91 - 1.76) |
| Course: F (df, p) | 7.43 (3, p < 0.001) | 4.39 (3, p = 0.004) | 3.77 (3, p = 0.010) | 3.38 (3, p = 0.018) | 6.06 (3, p = 0.000) | 4.16 (3, p = 0.006) | 3.24 (3, p = 0.021) | 3.09 (3, p = 0.026) |
| **Experiences of co-occuring Common Mental Disorders** |  |  |  |  |  |  |  |  |
| MDD | **2.44 (2.15 - 2.77)*** | - | **1.77 (1.55 - 2.03)*** | **1.41 (1.21 - 1.64)*** | **3.62 (2.74 - 4.80)*** | - | **2.58 (1.90 - 3.49)*** | **1.87 (1.36 - 2.56)*** |
| BP | **2.30 (1.94 - 2.72)*** | - | **1.77 (1.45 - 2.16)*** | **1.50 (1.23 - 1.83)*** | **3.60 (2.53 - 5.12)*** | - | **2.91 (1.95 - 4.35)*** | **2.24 (1.53 - 3.29)*** |
| GAD | **2.33 (2.10 - 2.59)*** | - | **1.18 (1.05 - 1.33)*** | **1.13 (1.01 - 1.26)*** | **3.61 (2.99 - 4.35)*** | - | **1.37 (1.09 - 1.72)*** | **1.31 (1.06 - 1.62)*** |
| PD | **2.37 (2.10 - 2.69)*** | - | 1.13 (0.98 - 1.29) | 1.13 (1.00 - 1.28) | **3.64 (2.90 - 4.57)*** | - | 1.29 (0.99 - 1.68) | **1.29 (1.01 - 1.65)*** |
| PTSD | **2.59 (2.26 - 2.98)*** | - | **1.55 (1.36 - 1.77)*** | **1.29 (1.13 - 1.47)*** | **3.72 (2.88 - 4.80)*** | - | **1.63 (1.25 - 2.11)*** | **1.34 (1.04 - 1.71)*** |
| DUD | **1.53 (1.32 - 1.77)*** | - | 1.08 (0.92 - 1.27) | 1.03 (0.88 - 1.21) | **2.05 (1.60 - 2.63)*** | - | 1.24 (0.96 - 1.62) | 1.12 (0.87 - 1.46) |
| AUD | 1.13 (1.00 - 1.28) | - | 1.04 (0.92 - 1.17) | **0.69 (0.54 - 0.88)*** | 1.10 (0.88 - 1.38) | - | 0.92 (0.74 - 1.14) | **0.52 (0.34 - 0.80)*** |
| ADHD | **1.93 (1.71 - 2.18)*** | - | 1.14 (1.00 - 1.29) | 0.78 (0.59 - 1.04) | **2.48 (1.97 - 3.12)*** | - | 1.07 (0.87 - 1.32) | 0.60 (0.35 - 1.00) |
| **Number of co-occuring Common Mental Disorders experienced** |  |  |  |  |  |  |  |  |
| 1 Other Disorder | **2.20 (1.87 - 2.60)*** | - | - | **1.77 (1.30 - 2.41)*** | **2.81 (2.01 - 3.92)*** | - | - | **2.02 (1.20 - 3.41)*** |
| 2 Other Disorders | **3.29 (2.77 - 3.91)*** | - | - | **2.73 (1.72 - 4.33)*** | **5.91 (4.42 - 7.91)*** | - | - | **4.05 (1.86 - 8.83)*** |
| 3 or more Other Disorders | **4.12 (3.39 - 5.00)*** | - | - | **3.58 (1.81 - 7.08)*** | **8.06 (5.67 - 11.44)*** | - | - | **6.37 (1.88 - 21.59)*** |
| Number of Disorders experienced: F (df, p) | 91.79 (3, p < 0.001) | - | - | 7.39 (3, p < 0.001) | 53.3 (3, p < 0.001) | - | - | 4.72 (3, p = 0.003) |
| Abbreviations: RR, Risk Ratio, 95% CI, 95% Confidence Interval  Risk ratios and results are from multivariable Poisson distribution and robust standard errors. Data was multiply imputed, m = 20.  All univariable models controlled for year of survey and incentivisation  All multivariable models controlled for all variables included in the model, and for year of survey and incentivisation  Regression models were built hierarchically with socio-demographic variables entered in block 1, co-occurring mental disorders added in block 2, and number of co-occurring mental disorders added in block 3.  * denotes significance difference, p < .05. | | | | | | | | |

Supplementary Table B CTNE: The associations of demographics variables and common mental disorders with 12-month suicide-related outcomes and transition along the suicidal trajectory, RR (95% CI)

|  | Suicide Attempt | | | | Ideation Only | | | |
| --- | --- | --- | --- | --- | --- | --- | --- | --- |
|  | Univariable | Block 1 | Block 2 | Block 3 | Univariable | Block 1 | Block 2 | Block 3 |
| **Age (Ref: 18 & Under)** |  |  |  |  |  |  |  |  |
| 19 | 0.71 (0.29 - 1.79) | 0.75 (0.33 - 1.72) | 0.67 (0.29 - 1.53) | 0.67 (0.29 - 1.53) | 0.97 (0.77 - 1.21) | 1.01 (0.82 - 1.23) | 1.02 (0.84 - 1.23) | 1.02 (0.84 - 1.24) |
| 20 & Older | 1.00 (0.50 - 1.98) | 0.96 (0.45 - 2.04) | 0.96 (0.49 - 1.88) | 0.92 (0.46 - 1.83) | 0.97 (0.79 - 1.18) | 0.95 (0.78 - 1.16) | 1.01 (0.83 - 1.23) | 1.01 (0.83 - 1.23) |
| Age Category: F (df, p) | 0.29 (2, p = 0.750) | 0.24 (2, p = 0.789) | 0.48 (2, p = 0.621) | 0.47 (2, p = 0.626) | 0.08 (2, p = 0.926) | 0.13 (2, p = 0.877) | 0.02 (2, p = 0.981) | 0.03 (2, p = 0.968) |
| **Gender (Ref: Female)** |  |  |  |  |  |  |  |  |
| Male | 0.49 (0.22 - 1.09) | 0.55 (0.24 - 1.29) | 0.90 (0.40 - 2.01) | 0.89 (0.40 - 1.96) | 1.10 (0.90 - 1.34) | 1.10 (0.91 - 1.33) | 0.97 (0.81 - 1.17) | 0.97 (0.81 - 1.16) |
| Gender Queer | **3.30 (1.43 - 7.62)*** | 0.49 (0.09 - 2.61) | 0.64 (0.11 - 3.76) | 0.64 (0.11 - 3.73) | 0.61 (0.36 - 1.03) | 1.73 (0.50 - 6.01) | 1.61 (0.49 - 5.32) | 1.59 (0.48 - 5.24) |
| Gender: F (df, p) | 6.24 (2, p = 0.002) | 1.08 (2, p = 0.340) | 0.12 (2, p = 0.884) | 0.13 (2, p = 0.877) | 2.29 (2, p = 0.103) | 0.83 (2, p = 0.438) | 0.35 (2, p = 0.704) | 0.34 (2, p = 0.709) |
| **Gender Modality (Ref: Cisgender)** |  |  |  |  |  |  |  |  |
| Transgender | **4.46 (2.14 - 9.26)*** | 3.45 (0.84 - 14.18) | 1.98 (0.45 - 8.79) | 2.07 (0.46 - 9.34) | **0.53 (0.32 - 0.86)*** | 0.40 (0.12 - 1.32) | 0.46 (0.15 - 1.41) | 0.46 (0.15 - 1.43) |
| **Sexual Orientation (Ref: Non-heterosexual)** |  |  |  |  |  |  |  |  |
| Non-heterosexual | **4.28 (2.33 - 7.88)*** | **3.33 (1.87 - 5.95)*** | 1.71 (0.94 - 3.10) | 1.74 (0.96 - 3.15) | **0.72 (0.60 - 0.87)*** | **0.79 (0.65 - 0.95)*** | 0.84 (0.70 - 1.00) | 0.84 (0.70 - 1.00) |
| **Enrolment Status (Ref: Full time)** |  |  |  |  |  |  |  |  |
| Part-time | 1.74 (0.61 - 4.95) | 1.88 (0.60 - 5.88) | 1.24 (0.47 - 3.28) | 1.31 (0.49 - 3.50) | 1.01 (0.70 - 1.46) | 0.99 (0.69 - 1.44) | 1.01 (0.71 - 1.43) | 1.01 (0.71 - 1.43) |
| **Parental Education (Ref: High School or Less)** |  |  |  |  |  |  |  |  |
| University or higher | 0.77 (0.44 - 1.34) | 0.78 (0.44 - 1.36) | 0.86 (0.48 - 1.54) | 0.86 (0.48 - 1.53) | 0.99 (0.84 - 1.18) | 1.01 (0.86 - 1.18) | 0.99 (0.85 - 1.15) | 0.99 (0.85 - 1.15) |
| **Ethnicity (Ref: Pākehā)** |  |  |  |  |  |  |  |  |
| Māori | 1.93 (0.84 - 4.44) | 1.97 (0.89 - 4.38) | 1.14 (0.53 - 2.46) | 1.15 (0.53 - 2.47) | 0.79 (0.61 - 1.02) | 0.78 (0.61 - 1.00) | 0.84 (0.66 - 1.06) | 0.83 (0.66 - 1.05) |
| Pacific Peoples | 1.73 (0.44 - 6.75) | 2.02 (0.52 - 7.89) | 1.56 (0.50 - 4.91) | 1.65 (0.51 - 5.30) | 0.92 (0.65 - 1.30) | 0.87 (0.62 - 1.20) | 0.92 (0.67 - 1.27) | 0.92 (0.67 - 1.26) |
| Asian | 0.97 (0.44 - 2.18) | 1.18 (0.55 - 2.55) | 1.54 (0.70 - 3.41) | 1.62 (0.73 - 3.59) | 1.05 (0.85 - 1.30) | 1.01 (0.83 - 1.24) | 0.97 (0.78 - 1.20) | 0.96 (0.77 - 1.20) |
| MELAA | 0.40 (0.00 - 320.22) | 0.97 (0.13 - 7.41) | 1.02 (0.15 - 6.98) | 1.08 (0.16 - 7.35) | 0.74 (0.40 - 1.36) | 0.70 (0.38 - 1.29) | 0.82 (0.45 - 1.51) | 0.81 (0.44 - 1.49) |
| Ethnicity: F (df, p) | 0.22 (4, p = 0.927) | 0.71 (4, p = 0.586) | 0.35 (4, p = 0.842) | 0.43 (4, p = 0.791) | 1.23 (4, p = 0.297) | 1.34 (4, p = 0.254) | 0.59 (4, p = 0.669) | 0.61 (4, p = 0.654) |
| **Course (Ref: Arts & Sciences)** |  |  |  |  |  |  |  |  |
| Medical Sciences | 0.58 (0.21 - 1.57) | 0.69 (0.27 - 1.79) | 0.63 (0.25 - 1.58) | 0.64 (0.26 - 1.59) | 1.10 (0.86 - 1.41) | 1.10 (0.86 - 1.40) | 1.11 (0.87 - 1.43) | 1.10 (0.85 - 1.43) |
| Commerce | 1.11 (0.54 - 2.31) | 1.06 (0.50 - 2.22) | 0.91 (0.46 - 1.80) | 0.92 (0.47 - 1.80) | 0.86 (0.68 - 1.09) | 0.87 (0.71 - 1.08) | 0.88 (0.71 - 1.08) | 0.87 (0.71 - 1.08) |
| Laws | 2.56 (0.98 - 6.73) | 2.02 (0.79 - 5.15) | 1.44 (0.55 - 3.80) | 1.50 (0.56 - 3.98) | 0.81 (0.52 - 1.25) | 0.84 (0.59 - 1.20) | 0.84 (0.59 - 1.21) | 0.83 (0.58 - 1.20) |
| Course: F (df, p) | 1.85 (3, p = 0.138) | 1.03 (3, p = 0.379) | 0.56 (3, p = 0.644) | 0.58 (3, p = 0.630) | 1.19 (3, p = 0.314) | 1.14 (3, p = 0.331) | 1.21 (3, p = 0.307) | 1.19 (3, p = 0.315) |
| **Experiences of co-occuring Common Mental Disorders** |  |  |  |  |  |  |  |  |
| MDD | **3.85 (1.84 - 8.08)*** | - | **4.81 (1.78 - 12.95)*** | **3.65 (1.37 - 9.73)*** | **0.69 (0.58 - 0.82)*** | - | **0.77 (0.63 - 0.95)*** | 0.79 (0.62 - 1.00) |
| BP | **10.81 (5.39 - 21.66)*** | - | **9.41 (2.86 - 31.00)*** | **7.34 (2.20 - 24.45)*** | **0.50 (0.28 - 0.89)*** | - | 0.57 (0.32 - 1.00) | 0.57 (0.32 - 1.02) |
| GAD | **6.12 (3.34 - 11.23)*** | - | 1.21 (0.62 - 2.34) | 1.19 (0.63 - 2.27) | **0.61 (0.49 - 0.77)*** | - | 0.82 (0.66 - 1.04) | 0.83 (0.66 - 1.05) |
| PD | **7.62 (3.87 - 15.02)*** | - | 1.69 (0.80 - 3.58) | 1.67 (0.81 - 3.45) | **0.60 (0.43 - 0.82)*** | - | 0.79 (0.61 - 1.03) | 0.79 (0.61 - 1.04) |
| PTSD | **9.97 (4.64 - 21.45)*** | - | **2.34 (1.02 - 5.35)*** | 2.07 (0.94 - 4.57) | **0.71 (0.55 - 0.91)*** | - | 0.90 (0.75 - 1.08) | 0.92 (0.72 - 1.17) |
| DUD | **5.84 (3.30 - 10.33)*** | - | 1.88 (0.93 - 3.82) | 1.64 (0.85 - 3.16) | **0.70 (0.52 - 0.95)*** | - | 0.81 (0.61 - 1.07) | 0.82 (0.61 - 1.09) |
| AUD | **2.74 (1.31 - 5.74)*** | - | 1.69 (0.82 - 3.46) | 0.84 (0.23 - 3.07) | 1.02 (0.85 - 1.23) | - | 1.11 (0.93 - 1.31) | 1.16 (0.84 - 1.60) |
| ADHD | **4.72 (2.67 - 8.36)*** | - | 1.25 (0.69 - 2.26) | 0.57 (0.11 - 2.95) | **0.75 (0.60 - 0.95)*** | - | 0.97 (0.78 - 1.21) | 0.99 (0.66 - 1.50) |
| **Number of co-occuring Common Mental Disorders experienced** |  |  |  |  |  |  |  |  |
| 1 Other Disorder | **5.22 (1.64 - 16.59)*** | - | - | 2.81 (0.00 - 3805.62) | 0.85 (0.69 - 1.05) | - | - | 1.00 (0.72 - 1.38) |
| 2 Other Disorders | **22.40 (7.54 - 66.60)*** | - | - | 7.96 (0.01 - 12008.81) | **0.56 (0.43 - 0.73)*** | - | - | 0.90 (0.51 - 1.61) |
| 3 or more Other Disorders | **41.93 (12.58 - 139.79)*** | - | - | 15.75 (0.01 - 38949.98) | **0.48 (0.29 - 0.77)*** | - | - | 0.96 (0.35 - 2.60) |
| Number of Disorders experienced: F (df, p) | 19.9 (3, p < 0.001) | - | - | 0.20 (3, p = 0.893) | 7.75 (3, p < 0.001) | - | - | 0.22 (3, p = 0.885) |
| Abbreviations: RR, Risk Ratio, 95% CI, 95% Confidence Interval  Risk ratios and results are from multivariable Poisson distribution and robust standard errors. Data was multiply imputed, m = 20.  All univariable models controlled for year of survey and incentivisation  All multivariable models controlled for all variables included in the model, and for year of survey and incentivisation  Regression models were built hierarchically with socio-demographic variables entered in block 1, co-occurring mental disorders added in block 2, and number of co-occurring mental disorders added in block 3.  * denotes significance difference, p < .05. | | | | | | | | |

Supplementary Table B CTNE: The associations of demographics variables and common mental disorders with 12-month suicide-related outcomes and transition along the suicidal trajectory, RR (95% CI)

|  | Plan If Ideation | | | | Attempt If Ideation | | | |
| --- | --- | --- | --- | --- | --- | --- | --- | --- |
|  | Univariable | Block 1 | Block 2 | Block 3 | Univariable | Block 1 | Block 2 | Block 3 |
| **Age (Ref: 18 & Under)** |  |  |  |  |  |  |  |  |
| 19 | 1.03 (0.82 - 1.29) | 1.00 (0.81 - 1.23) | 0.98 (0.80 - 1.21) | 0.98 (0.80 - 1.20) | 0.76 (0.31 - 1.85) | 0.76 (0.35 - 1.68) | 0.70 (0.32 - 1.56) | 0.71 (0.32 - 1.57) |
| 20 & Older | 1.03 (0.84 - 1.27) | 1.04 (0.85 - 1.28) | 0.98 (0.81 - 1.20) | 0.98 (0.80 - 1.19) | 1.07 (0.54 - 2.13) | 1.05 (0.52 - 2.11) | 1.04 (0.54 - 1.99) | 1.01 (0.52 - 1.96) |
| Age Category: F (df, p) | 0.06 (2, p = 0.940) | 0.08 (2, p = 0.921) | 0.02 (2, p = 0.979) | 0.04 (2, p = 0.958) | 0.24 (2, p = 0.787) | 0.26 (2, p = 0.769) | 0.43 (2, p = 0.652) | 0.39 (2, p = 0.676) |
| **Gender (Ref: Female)** |  |  |  |  |  |  |  |  |
| Male | 0.90 (0.72 - 1.13) | 0.90 (0.71 - 1.13) | 1.03 (0.82 - 1.29) | 1.04 (0.83 - 1.30) | 0.65 (0.29 - 1.47) | 0.65 (0.28 - 1.51) | 0.90 (0.41 - 2.00) | 0.90 (0.41 - 1.96) |
| Gender Queer | **1.39 (1.08 - 1.78)*** | 0.82 (0.55 - 1.22) | 0.88 (0.60 - 1.30) | 0.89 (0.61 - 1.32) | 1.78 (0.77 - 4.11) | 0.54 (0.10 - 2.91) | 0.62 (0.11 - 3.66) | 0.61 (0.10 - 3.59) |
| Gender: F (df, p) | 3.98 (2, p = 0.020) | 0.68 (2, p = 0.509) | 0.38 (2, p = 0.682) | 0.36 (2, p = 0.698) | 1.74 (2, p = 0.178) | 0.62 (2, p = 0.541) | 0.14 (2, p = 0.869) | 0.15 (2, p = 0.857) |
| **Gender Modality (Ref: Cisgender)** |  |  |  |  |  |  |  |  |
| Transgender | **1.51 (1.23 - 1.85)*** | **1.47 (1.04 - 2.09)*** | 1.33 (0.94 - 1.86) | 1.31 (0.94 - 1.84) | **2.14 (1.07 - 4.28)*** | 2.45 (0.60 - 9.97) | 1.92 (0.45 - 8.16) | 2.02 (0.47 - 8.69) |
| **Sexual Orientation (Ref: Non-heterosexual)** |  |  |  |  |  |  |  |  |
| Non-heterosexual | **1.37 (1.16 - 1.63)*** | **1.27 (1.07 - 1.51)*** | **1.21 (1.02 - 1.43)*** | **1.21 (1.02 - 1.43)*** | **2.07 (1.17 - 3.66)*** | 1.74 (0.98 - 3.10) | 1.36 (0.78 - 2.40) | 1.38 (0.79 - 2.41) |
| **Enrolment Status (Ref: Full time)** |  |  |  |  |  |  |  |  |
| Part-time | 0.98 (0.65 - 1.46) | 0.99 (0.68 - 1.46) | 0.95 (0.67 - 1.36) | 0.96 (0.67 - 1.37) | 1.80 (0.66 - 4.89) | 1.75 (0.62 - 4.94) | 1.30 (0.51 - 3.31) | 1.36 (0.54 - 3.47) |
| **Parental Education (Ref: High School or Less)** |  |  |  |  |  |  |  |  |
| University or higher | 1.01 (0.85 - 1.20) | 1.00 (0.85 - 1.17) | 1.02 (0.87 - 1.20) | 1.02 (0.87 - 1.19) | 0.79 (0.46 - 1.36) | 0.82 (0.48 - 1.40) | 0.87 (0.50 - 1.51) | 0.87 (0.50 - 1.49) |
| **Ethnicity (Ref: Pākehā)** |  |  |  |  |  |  |  |  |
| Māori | 1.27 (0.98 - 1.63) | 1.27 (1.00 - 1.61) | 1.18 (0.94 - 1.47) | 1.17 (0.94 - 1.46) | 1.81 (0.82 - 4.01) | 1.75 (0.82 - 3.75) | 1.14 (0.54 - 2.41) | 1.16 (0.55 - 2.44) |
| Pacific Peoples | 1.09 (0.73 - 1.63) | 1.15 (0.80 - 1.66) | 1.08 (0.76 - 1.54) | 1.09 (0.77 - 1.54) | 1.68 (0.45 - 6.29) | 1.83 (0.48 - 7.01) | 1.70 (0.59 - 4.89) | 1.82 (0.63 - 5.26) |
| Asian | 0.95 (0.74 - 1.21) | 0.98 (0.78 - 1.25) | 1.01 (0.80 - 1.29) | 1.04 (0.81 - 1.33) | 0.99 (0.45 - 2.18) | 1.03 (0.49 - 2.19) | 1.49 (0.69 - 3.23) | 1.57 (0.71 - 3.44) |
| MELAA | 1.26 (0.83 - 1.91) | 1.30 (0.86 - 1.98) | 1.12 (0.74 - 1.68) | 1.13 (0.74 - 1.70) | 0.37 (0.00 - 397.11) | 0.93 (0.12 - 7.04) | 0.97 (0.15 - 6.46) | 1.01 (0.15 - 6.71) |
| Ethnicity: F (df, p) | 1.27 (4, p = 0.279) | 1.38 (4, p = 0.241) | 0.52 (4, p = 0.721) | 0.51 (4, p = 0.729) | 0.18 (4, p = 0.946) | 0.52 (4, p = 0.721) | 0.46 (4, p = 0.766) | 0.54 (4, p = 0.704) |
| **Course (Ref: Arts & Sciences)** |  |  |  |  |  |  |  |  |
| Medical Sciences | 0.89 (0.64 - 1.23) | 0.89 (0.65 - 1.22) | 0.86 (0.63 - 1.18) | 0.88 (0.64 - 1.20) | 0.72 (0.27 - 1.90) | 0.78 (0.31 - 1.96) | 0.70 (0.28 - 1.75) | 0.73 (0.30 - 1.81) |
| Commerce | 1.16 (0.94 - 1.42) | 1.13 (0.94 - 1.37) | 1.13 (0.93 - 1.36) | 1.13 (0.94 - 1.36) | 0.94 (0.46 - 1.94) | 0.94 (0.46 - 1.93) | 0.95 (0.49 - 1.83) | 0.96 (0.50 - 1.85) |
| Laws | 1.21 (0.87 - 1.67) | 1.17 (0.88 - 1.55) | 1.16 (0.86 - 1.56) | 1.19 (0.88 - 1.60) | 1.94 (0.73 - 5.18) | 1.87 (0.73 - 4.78) | 1.47 (0.55 - 3.91) | 1.54 (0.57 - 4.12) |
| Course: F (df, p) | 1.33 (3, p = 0.263) | 1.15 (3, p = 0.328) | 1.25 (3, p = 0.291) | 1.26 (3, p = 0.286) | 0.9 (3, p = 0.441) | 0.82 (3, p = 0.486) | 0.43 (3, p = 0.732) | 0.44 (3, p = 0.724) |
| **Experiences of co-occuring Common Mental Disorders** |  |  |  |  |  |  |  |  |
| MDD | **1.47 (1.23 - 1.74)*** | - | **1.43 (1.13 - 1.81)*** | 1.32 (1.00 - 1.73) | 1.55 (0.85 - 2.85) | - | **2.72 (1.08 - 6.83)*** | 2.51 (0.98 - 6.46) |
| BP | **1.54 (1.20 - 1.97)*** | - | **1.57 (1.14 - 2.16)*** | **1.47 (1.06 - 2.04)*** | **4.62 (2.07 - 10.30)*** | - | **5.06 (1.67 - 15.27)*** | **4.70 (1.51 - 14.66)*** |
| GAD | **1.54 (1.29 - 1.85)*** | - | 1.18 (0.98 - 1.42) | 1.16 (0.97 - 1.39) | **2.69 (1.38 - 5.22)*** | - | 1.14 (0.61 - 2.12) | 1.14 (0.62 - 2.10) |
| PD | **1.52 (1.18 - 1.94)*** | - | 1.16 (0.96 - 1.40) | 1.15 (0.95 - 1.38) | **3.24 (1.64 - 6.42)*** | - | 1.53 (0.74 - 3.17) | 1.51 (0.74 - 3.09) |
| PTSD | **1.43 (1.08 - 1.90)*** | - | 1.13 (0.93 - 1.38) | 1.07 (0.88 - 1.31) | **3.87 (1.82 - 8.19)*** | - | 1.77 (0.83 - 3.76) | 1.72 (0.81 - 3.68) |
| DUD | **1.32 (1.09 - 1.61)*** | - | 1.18 (0.96 - 1.47) | 1.13 (0.90 - 1.41) | **3.79 (2.18 - 6.60)*** | - | 1.83 (0.94 - 3.58) | 1.63 (0.86 - 3.11) |
| AUD | 0.97 (0.81 - 1.17) | - | 0.89 (0.74 - 1.06) | 0.71 (0.50 - 1.02) | **2.42 (1.15 - 5.09)*** | - | 1.64 (0.81 - 3.30) | 0.98 (0.26 - 3.72) |
| ADHD | **1.27 (1.06 - 1.53)*** | - | 0.99 (0.83 - 1.18) | 0.79 (0.51 - 1.21) | **2.45 (1.44 - 4.18)*** | - | 1.22 (0.70 - 2.12) | 0.66 (0.12 - 3.55) |
| **Number of co-occuring Common Mental Disorders experienced** |  |  |  |  |  |  |  |  |
| 1 Other Disorder | 1.27 (0.93 - 1.75) | - | - | 1.12 (0.72 - 1.74) | 2.41 (0.75 - 7.68) | - | - | 1.50 (0.00 - 1812.17) |
| 2 Other Disorders | **1.79 (1.35 - 2.38)*** | - | - | 1.53 (0.80 - 2.93) | **6.80 (2.25 - 20.48)*** | - | - | 3.37 (0.00 - 4159.24) |
| 3 or more Other Disorders | **1.94 (1.37 - 2.73)*** | - | - | 1.81 (0.67 - 4.90) | **10.19 (2.93 - 35.49)*** | - | - | 5.73 (0.00 - 10962.60) |
| Number of Disorders experienced: F (df, p) | 9.12 (3, p < 0.001) | - | - | 0.90 (3, p = 0.440) | 8.42 (3, p < 0.001) | - | - | 0.09 (3, p = 0.968) |
| Abbreviations: RR, Risk Ratio, 95% CI, 95% Confidence Interval  Risk ratios and results are from multivariable Poisson distribution and robust standard errors. Data was multiply imputed, m = 20.  All univariable models controlled for year of survey and incentivisation  All multivariable models controlled for all variables included in the model, and for year of survey and incentivisation  Regression models were built hierarchically with socio-demographic variables entered in block 1, co-occurring mental disorders added in block 2, and number of co-occurring mental disorders added in block 3.  * denotes significance difference, p < .05. | | | | | | | | |

Supplementary Table B CTNE: The associations of demographics variables and common mental disorders with 12-month suicide-related outcomes and transition along the suicidal trajectory, RR (95% CI)

|  | Plan No Attempt | | | Attempt If Plan | | | |  |  |  |
| --- | --- | --- | --- | --- | --- | --- | --- | --- | --- | --- |
|  | Univariable | Block 1 | Block 2 | | Block 3 | Univariable | Block 1 | | Block 2 | Block 3 |
| **Age (Ref: 18 & Under)** |  |  |  | |  |  |  | |  |  |
| 19 | 1.04 (0.93 - 1.17) | 1.04 (0.93 - 1.16) | 1.06 (0.95 - 1.18) | | 1.05 (0.94 - 1.17) | 0.74 (0.30 - 1.81) | 0.75 (0.34 - 1.67) | | 0.69 (0.31 - 1.51) | 0.69 (0.31 - 1.52) |
| 20 & Older | 0.99 (0.87 - 1.13) | 1.00 (0.88 - 1.13) | 1.00 (0.89 - 1.13) | | 1.00 (0.88 - 1.12) | 1.03 (0.52 - 2.05) | 1.02 (0.52 - 2.00) | | 1.05 (0.56 - 1.99) | 1.02 (0.53 - 1.95) |
| Age Category: F (df, p) | 0.28 (2, p = 0.752) | 0.23 (2, p = 0.797) | 0.50 (2, p = 0.607) | | 0.45 (2, p = 0.635) | 0.26 (2, p = 0.768) | 0.28 (2, p = 0.757) | | 0.50 (2, p = 0.605) | 0.46 (2, p = 0.633) |
| **Gender (Ref: Female)** |  |  |  | |  |  |  | |  |  |
| Male | 1.05 (0.93 - 1.17) | 1.04 (0.93 - 1.17) | 1.01 (0.90 - 1.13) | | 1.01 (0.90 - 1.13) | 0.73 (0.33 - 1.64) | 0.73 (0.32 - 1.68) | | 0.87 (0.40 - 1.88) | 0.88 (0.42 - 1.86) |
| Gender Queer | 0.94 (0.77 - 1.15) | 1.06 (0.72 - 1.56) | 1.00 (0.67 - 1.50) | | 1.00 (0.66 - 1.51) | 1.30 (0.56 - 2.99) | 0.65 (0.13 - 3.37) | | 0.78 (0.14 - 4.43) | 0.77 (0.13 - 4.50) |
| Gender: F (df, p) | 0.58 (2, p = 0.559) | 0.31 (2, p = 0.733) | 0.01 (2, p = 0.992) | | 0.01 (2, p = 0.993) | 0.58 (2, p = 0.560) | 0.33 (2, p = 0.716) | | 0.09 (2, p = 0.917) | 0.08 (2, p = 0.923) |
| **Gender Modality (Ref: Cisgender)** |  |  |  | |  |  |  | |  |  |
| Transgender | 0.93 (0.79 - 1.09) | 0.91 (0.67 - 1.25) | 0.97 (0.70 - 1.34) | | 0.96 (0.69 - 1.34) | 1.44 (0.72 - 2.88) | 1.68 (0.43 - 6.60) | | 1.43 (0.34 - 5.95) | 1.48 (0.35 - 6.28) |
| **Sexual Orientation (Ref: Non-heterosexual)** |  |  |  | |  |  |  | |  |  |
| Non-heterosexual | 0.94 (0.85 - 1.03) | 0.96 (0.87 - 1.05) | 0.97 (0.89 - 1.06) | | 0.97 (0.89 - 1.06) | 1.49 (0.84 - 2.63) | 1.32 (0.76 - 2.30) | | 1.11 (0.65 - 1.91) | 1.14 (0.66 - 1.95) |
| **Enrolment Status (Ref: Full time)** |  |  |  | |  |  |  | |  |  |
| Part-time | 0.86 (0.64 - 1.17) | 0.86 (0.64 - 1.17) | 0.88 (0.68 - 1.15) | | 0.88 (0.68 - 1.15) | 1.81 (0.73 - 4.51) | 1.80 (0.73 - 4.47) | | 1.39 (0.60 - 3.25) | 1.43 (0.62 - 3.32) |
| **Parental Education (Ref: High School or Less)** |  |  |  | |  |  |  | |  |  |
| University or higher | 1.04 (0.95 - 1.15) | 1.04 (0.95 - 1.15) | 1.03 (0.93 - 1.13) | | 1.03 (0.93 - 1.13) | 0.79 (0.47 - 1.32) | 0.81 (0.48 - 1.35) | | 0.84 (0.50 - 1.42) | 0.84 (0.50 - 1.43) |
| **Ethnicity (Ref: Pākehā)** |  |  |  | |  |  |  | |  |  |
| Māori | 0.94 (0.82 - 1.08) | 0.94 (0.82 - 1.08) | 0.99 (0.86 - 1.14) | | 0.99 (0.86 - 1.13) | 1.41 (0.62 - 3.18) | 1.43 (0.66 - 3.08) | | 1.07 (0.51 - 2.26) | 1.10 (0.52 - 2.32) |
| Pacific Peoples | 0.92 (0.70 - 1.23) | 0.91 (0.69 - 1.22) | 0.91 (0.72 - 1.16) | | 0.91 (0.72 - 1.16) | 1.47 (0.36 - 5.99) | 1.58 (0.39 - 6.46) | | 1.35 (0.45 - 4.09) | 1.44 (0.47 - 4.39) |
| Asian | 0.99 (0.87 - 1.12) | 0.99 (0.87 - 1.12) | 0.92 (0.81 - 1.04) | | 0.93 (0.82 - 1.05) | 1.08 (0.51 - 2.26) | 1.06 (0.51 - 2.20) | | 1.49 (0.73 - 3.04) | 1.54 (0.75 - 3.16) |
| MELAA | 1.04 (0.82 - 1.32) | 1.00 (0.78 - 1.28) | 0.97 (0.76 - 1.25) | | 0.97 (0.76 - 1.25) | 0.30 (0.00 - 405.27) | 0.80 (0.11 - 5.98) | | 0.93 (0.15 - 5.97) | 0.99 (0.15 - 6.30) |
| Ethnicity: F (df, p) | 0.26 (4, p = 0.902) | 0.21 (4, p = 0.932) | 0.41 (4, p = 0.799) | | 0.39 (4, p = 0.815) | 0.15 (4, p = 0.964) | 0.25 (4, p = 0.908) | | 0.32 (4, p = 0.863) | 0.37 (4, p = 0.827) |
| **Course (Ref: Arts & Sciences)** |  |  |  | |  |  |  | |  |  |
| Medical Sciences | 1.02 (0.88 - 1.20) | 1.01 (0.87 - 1.17) | 1.02 (0.88 - 1.18) | | 1.01 (0.87 - 1.17) | 0.84 (0.31 - 2.32) | 0.91 (0.36 - 2.31) | | 0.80 (0.31 - 2.08) | 0.83 (0.32 - 2.11) |
| Commerce | 1.03 (0.92 - 1.14) | 1.02 (0.91 - 1.14) | 1.01 (0.90 - 1.12) | | 1.00 (0.90 - 1.11) | 0.84 (0.41 - 1.72) | 0.87 (0.43 - 1.75) | | 0.87 (0.46 - 1.62) | 0.88 (0.48 - 1.63) |
| Laws | 0.88 (0.67 - 1.15) | 0.88 (0.68 - 1.15) | 0.90 (0.70 - 1.17) | | 0.91 (0.70 - 1.19) | 1.67 (0.67 - 4.17) | 1.69 (0.68 - 4.22) | | 1.27 (0.48 - 3.38) | 1.31 (0.49 - 3.49) |
| Course: F (df, p) | 0.4 (3, p = 0.750) | 0.34 (3, p = 0.794) | 0.22 (3, p = 0.881) | | 0.17 (3, p = 0.919) | 0.63 (3, p = 0.596) | 0.63 (3, p = 0.597) | | 0.25 (3, p = 0.860) | 0.24 (3, p = 0.866) |
| **Experiences of co-occuring Common Mental Disorders** |  |  |  | |  |  |  | |  |  |
| MDD | 0.99 (0.90 - 1.09) | - | 0.95 (0.87 - 1.04) | | 0.93 (0.84 - 1.02) | 1.07 (0.57 - 2.01) | - | | 2.08 (0.84 - 5.16) | 2.04 (0.81 - 5.13) |
| BP | **0.74 (0.56 - 0.97)*** | - | **0.76 (0.60 - 0.96)*** | | **0.75 (0.59 - 0.95)*** | **3.02 (1.41 - 6.47)*** | - | | **3.69 (1.32 - 10.33)*** | **3.63 (1.24 - 10.65)*** |
| GAD | 0.91 (0.82 - 1.02) | - | 1.01 (0.91 - 1.11) | | 1.00 (0.90 - 1.11) | 1.71 (0.92 - 3.20) | - | | 0.93 (0.51 - 1.70) | 0.94 (0.51 - 1.73) |
| PD | 0.87 (0.77 - 1.00) | - | 0.94 (0.82 - 1.08) | | 0.95 (0.83 - 1.08) | **2.08 (1.06 - 4.08)*** | - | | 1.34 (0.68 - 2.63) | 1.32 (0.68 - 2.58) |
| PTSD | **0.87 (0.79 - 0.96)*** | - | 0.95 (0.87 - 1.05) | | 0.94 (0.85 - 1.04) | **2.69 (1.25 - 5.80)*** | - | | 1.53 (0.71 - 3.30) | 1.53 (0.71 - 3.34) |
| DUD | **0.77 (0.63 - 0.94)*** | - | 0.85 (0.70 - 1.03) | | 0.86 (0.71 - 1.03) | **2.90 (1.70 - 4.95)*** | - | | 1.65 (0.86 - 3.16) | 1.49 (0.81 - 2.74) |
| AUD | **0.87 (0.78 - 0.96)*** | - | 0.91 (0.83 - 1.01) | | 0.91 (0.75 - 1.11) | **2.46 (1.18 - 5.12)*** | - | | 1.79 (0.93 - 3.45) | 1.16 (0.30 - 4.55) |
| ADHD | 0.89 (0.80 - 1.00) | - | 0.97 (0.86 - 1.09) | | 0.99 (0.82 - 1.20) | **1.83 (1.07 - 3.12)*** | - | | 1.14 (0.64 - 2.03) | 0.66 (0.12 - 3.48) |
| **Number of co-occuring Common Mental Disorders experienced** |  |  |  | |  |  |  | |  |  |
| 1 Other Disorder | 0.95 (0.87 - 1.03) | - | - | | 1.10 (0.95 - 1.28) | 1.88 (0.62 - 5.65) | - | | - | 1.31 (0.00 - 1637.48) |
| 2 Other Disorders | **0.84 (0.75 - 0.95)*** | - | - | | 1.11 (0.83 - 1.48) | **3.73 (1.28 - 10.89)*** | - | | - | 2.50 (0.00 - 3147.70) |
| 3 or more Other Disorders | **0.76 (0.61 - 0.96)*** | - | - | | 1.03 (0.63 - 1.66) | **5.06 (1.59 - 16.09)*** | - | | - | 4.17 (0.00 - 6903.64) |
| Number of Disorders experienced: F (df, p) | 4.13 (3, p = 0.007) | - | - | | 1.14 (3, p = 0.334) | 4.06 (3, p = 0.007) | - | | - | 0.05 (3, p = 0.987) |
| Abbreviations: RR, Risk Ratio, 95% CI, 95% Confidence Interval  Risk ratios and results are from multivariable Poisson distribution and robust standard errors. Data was multiply imputed, m = 20.  All univariable models controlled for year of survey and incentivisation  All multivariable models controlled for all variables included in the model, and for year of survey and incentivisation  Regression models were built hierarchically with socio-demographic variables entered in block 1, co-occurring mental disorders added in block 2, and number of co-occurring mental disorders added in block 3.  * denotes significance difference, p < .05. | | | | | | | | | | |

Supplementary Table C: The associations of demographics variables and common mental disorders with persistence of suicide-related outcomes, RR (95% CI)

|  | Suicidal Ideation |  |  |  | Suicide Plan |  |  |  |
| --- | --- | --- | --- | --- | --- | --- | --- | --- |
|  | Univariable | Block 1 | Block 2 | Block 3 | Univariable | Block 1 | Block 2 | Block 3 |
| **Age (Ref: 18 & Under)** |  |  |  |  |  |  |  |  |
| 19 | 0.96 (0.85 - 1.08) | 0.97 (0.86 - 1.09) | 0.96 (0.85 - 1.08) | 0.96 (0.85 - 1.08) | 0.99 (0.81 - 1.22) | 1.00 (0.82 - 1.21) | 0.98 (0.80 - 1.20) | 0.98 (0.80 - 1.19) |
| 20 & Older | **0.84 (0.74 - 0.95)*** | **0.85 (0.76 - 0.96)*** | **0.84 (0.75 - 0.95)*** | **0.84 (0.75 - 0.95)*** | 0.90 (0.74 - 1.09) | 0.90 (0.75 - 1.09) | 0.88 (0.73 - 1.06) | 0.87 (0.72 - 1.05) |
| Age Category: F (df, p) | 3.29 (2, p = 0.038) | 2.78 (2, p = 0.063) | 3.48 (2, p = 0.032) | 3.61 (2, p = 0.028) | 0.52 (2, p = 0.592) | 0.52 (2, p = 0.592) | 0.83 (2, p = 0.436) | 0.94 (2, p = 0.391) |
| **Gender (Ref: Female)** |  |  |  |  |  |  |  |  |
| Male | 0.95 (0.85 - 1.06) | 0.97 (0.87 - 1.09) | 1.06 (0.95 - 1.18) | 1.06 (0.95 - 1.18) | 0.87 (0.71 - 1.07) | 0.87 (0.70 - 1.07) | 0.97 (0.79 - 1.19) | 0.97 (0.79 - 1.20) |
| Gender Queer | **1.23 (1.05 - 1.44)*** | 0.93 (0.72 - 1.20) | 0.99 (0.76 - 1.29) | 0.99 (0.77 - 1.28) | 1.13 (0.85 - 1.50) | 0.72 (0.50 - 1.04) | 0.80 (0.55 - 1.16) | 0.81 (0.56 - 1.17) |
| Gender: F (df, p) | 4.39 (2, p = 0.013) | 0.19 (2, p = 0.825) | 0.61 (2, p = 0.542) | 0.62 (2, p = 0.540) | 1.35 (2, p = 0.260) | 1.66 (2, p = 0.191) | 0.63 (2, p = 0.532) | 0.58 (2, p = 0.560) |
| **Gender Modality (Ref: Cisgender)** |  |  |  |  |  |  |  |  |
| Transgender | **1.27 (1.11 - 1.46)*** | 1.19 (0.96 - 1.49) | 1.09 (0.86 - 1.37) | 1.08 (0.86 - 1.36) | 1.26 (1.00 - 1.58) | **1.46 (1.07 - 2.00)*** | 1.28 (0.94 - 1.75) | 1.26 (0.93 - 1.73) |
| **Sexual Orientation (Ref: Non-heterosexual)** |  |  |  |  |  |  |  |  |
| Non-heterosexual | **1.22 (1.11 - 1.34)*** | **1.17 (1.07 - 1.28)*** | **1.12 (1.02 - 1.22)*** | **1.12 (1.02 - 1.23)*** | **1.24 (1.06 - 1.45)*** | 1.16 (0.99 - 1.35) | 1.10 (0.94 - 1.29) | 1.10 (0.94 - 1.29) |
| **Enrolment Status (Ref: Full time)** |  |  |  |  |  |  |  |  |
| Part-time | 0.86 (0.69 - 1.08) | 0.92 (0.75 - 1.14) | 0.92 (0.74 - 1.14) | 0.93 (0.75 - 1.15) | 1.02 (0.71 - 1.47) | 1.04 (0.72 - 1.48) | 0.95 (0.67 - 1.35) | 0.96 (0.68 - 1.36) |
| **Parental Education (Ref: High School or Less)** |  |  |  |  |  |  |  |  |
| University or higher | 1.00 (0.90 - 1.11) | 0.97 (0.88 - 1.07) | 0.99 (0.90 - 1.09) | 0.99 (0.90 - 1.09) | 1.02 (0.86 - 1.21) | 0.99 (0.84 - 1.15) | 1.02 (0.87 - 1.18) | 1.02 (0.88 - 1.18) |
| **Ethnicity (Ref: Pākehā)** |  |  |  |  |  |  |  |  |
| Māori | 1.08 (0.92 - 1.27) | 1.07 (0.92 - 1.24) | 1.00 (0.87 - 1.15) | 1.00 (0.87 - 1.15) | 1.12 (0.85 - 1.47) | 1.15 (0.89 - 1.50) | 1.06 (0.83 - 1.35) | 1.06 (0.84 - 1.35) |
| Pacific Peoples | 1.04 (0.83 - 1.31) | 1.06 (0.86 - 1.30) | 1.01 (0.83 - 1.23) | 1.01 (0.83 - 1.23) | 1.09 (0.70 - 1.69) | 1.13 (0.74 - 1.72) | 1.04 (0.71 - 1.53) | 1.05 (0.71 - 1.54) |
| Asian | 0.95 (0.82 - 1.09) | 0.98 (0.85 - 1.12) | 1.02 (0.89 - 1.17) | 1.04 (0.90 - 1.19) | 1.00 (0.78 - 1.28) | 1.04 (0.82 - 1.32) | 1.05 (0.82 - 1.33) | 1.06 (0.83 - 1.35) |
| MELAA | 1.07 (0.85 - 1.34) | 1.07 (0.86 - 1.34) | 1.01 (0.82 - 1.24) | 1.01 (0.82 - 1.25) | 1.16 (0.76 - 1.77) | 1.20 (0.79 - 1.84) | 1.08 (0.72 - 1.61) | 1.09 (0.73 - 1.63) |
| Ethnicity: F (df, p) | 0.57 (4, p = 0.686) | 0.40 (4, p = 0.806) | 0.02 (4, p = 0.999) | 0.07 (4, p = 0.990) | 0.29 (4, p = 0.883) | 0.47 (4, p = 0.759) | 0.10 (4, p = 0.981) | 0.13 (4, p = 0.970) |
| **Course (Ref: Arts & Sciences)** |  |  |  |  |  |  |  |  |
| Medical Sciences | 0.95 (0.80 - 1.13) | 0.92 (0.78 - 1.09) | 0.90 (0.77 - 1.06) | 0.91 (0.77 - 1.06) | 0.97 (0.70 - 1.33) | 0.96 (0.72 - 1.27) | 0.92 (0.69 - 1.22) | 0.92 (0.69 - 1.23) |
| Commerce | 1.04 (0.93 - 1.17) | 1.03 (0.92 - 1.15) | 1.02 (0.91 - 1.14) | 1.02 (0.91 - 1.13) | 1.13 (0.94 - 1.35) | 1.10 (0.93 - 1.31) | 1.10 (0.93 - 1.30) | 1.09 (0.92 - 1.30) |
| Laws | 1.03 (0.81 - 1.29) | 1.02 (0.85 - 1.23) | 1.01 (0.85 - 1.21) | 1.01 (0.85 - 1.21) | 1.13 (0.85 - 1.51) | 1.13 (0.87 - 1.48) | 1.10 (0.83 - 1.46) | 1.12 (0.84 - 1.48) |
| Course: F (df, p) | 0.34 (3, p = 0.794) | 0.47 (3, p = 0.702) | 0.66 (3, p = 0.574) | 0.61 (3, p = 0.610) | 0.78 (3, p = 0.507) | 0.68 (3, p = 0.565) | 0.69 (3, p = 0.558) | 0.66 (3, p = 0.574) |
| **Experiences of co-occuring Common Mental Disorders** |  |  |  |  |  |  |  |  |
| MDD | **1.34 (1.21 - 1.48)*** | - | **1.25 (1.11 - 1.39)*** | **1.18 (1.04 - 1.35)*** | **1.35 (1.13 - 1.62)*** | - | **1.40 (1.10 - 1.77)*** | 1.31 (1.00 - 1.72) |
| BP | **1.31 (1.13 - 1.52)*** | - | **1.24 (1.06 - 1.44)*** | **1.20 (1.02 - 1.40)*** | **1.37 (1.10 - 1.71)*** | - | **1.50 (1.12 - 1.99)*** | **1.42 (1.06 - 1.92)*** |
| GAD | **1.36 (1.24 - 1.48)*** | - | **1.12 (1.02 - 1.24)*** | **1.11 (1.01 - 1.22)*** | **1.39 (1.16 - 1.67)*** | - | 1.14 (0.95 - 1.35) | 1.12 (0.94 - 1.33) |
| PD | **1.32 (1.20 - 1.46)*** | - | 1.05 (0.94 - 1.17) | 1.05 (0.94 - 1.17) | 1.30 (1.00 - 1.67) | - | 1.03 (0.85 - 1.24) | 1.03 (0.86 - 1.23) |
| PTSD | **1.34 (1.21 - 1.49)*** | - | **1.15 (1.04 - 1.27)*** | 1.10 (0.98 - 1.23) | **1.34 (1.09 - 1.65)*** | - | 1.10 (0.90 - 1.34) | 1.06 (0.86 - 1.31) |
| DUD | **1.19 (1.05 - 1.33)*** | - | 1.07 (0.94 - 1.21) | 1.06 (0.93 - 1.21) | **1.24 (1.02 - 1.49)*** | - | 1.18 (0.96 - 1.44) | 1.15 (0.93 - 1.43) |
| AUD | 1.09 (0.98 - 1.21) | - | 1.01 (0.91 - 1.11) | 0.92 (0.74 - 1.15) | 1.00 (0.83 - 1.20) | - | 0.92 (0.77 - 1.09) | 0.80 (0.55 - 1.16) |
| ADHD | **1.23 (1.11 - 1.37)*** | - | 1.06 (0.96 - 1.17) | 0.99 (0.78 - 1.26) | **1.27 (1.07 - 1.51)*** | - | 1.04 (0.87 - 1.25) | 0.92 (0.59 - 1.42) |
| **Number of co-occuring Common Mental Disorders experienced** |  |  |  |  |  |  |  |  |
| 1 Other Disorder | **1.34 (1.16 - 1.54)*** | - | - | 1.20 (0.91 - 1.58) | 1.24 (0.88 - 1.73) | - | - | 1.12 (0.73 - 1.72) |
| 2 Other Disorders | **1.58 (1.37 - 1.81)*** | - | - | 1.29 (0.85 - 1.96) | **1.70 (1.29 - 2.25)*** | - | - | 1.38 (0.73 - 2.64) |
| 3 or more Other Disorders | **1.64 (1.38 - 1.94)*** | - | - | 1.35 (0.73 - 2.49) | **1.71 (1.23 - 2.38)*** | - | - | 1.45 (0.54 - 3.91) |
| Number of Disorders experienced: F (df, p) | 15.74 (3, p < 0.001) | - | - | 0.74 (3, p = 0.527) | 6.4 (3, p = 0.000) | - | - | 0.56 (3, p = 0.639) |
| Abbreviations: RR, Risk Ratio, 95% CI, 95% Confidence Interval  Risk ratios and results are from multivariable Poisson distribution and robust standard errors. Data was multiply imputed, m = 20.  All univariable models controlled for year of survey and incentivisation  All multivariable models controlled for all variables included in the model, and for year of survey and incentivisation  Regression models were built hierarchically with socio-demographic variables entered in block 1, co-occurring mental disorders added in block 2, and number of co-occurring mental disorders added in block 3.  Co-occuring mental disorders are experienced in the past 12 months  * denotes significance difference, p < .05. | | | | | | | | |

Supplementary Table C CTNE : The associations of demographics variables and common mental disorders with persistence of suicide-related outcomes, RR (95% CI)

|  | Suicide Attempt | | | |
| --- | --- | --- | --- | --- |
|  | Univariable | Block 1 | Block 2 | Block 3 |
| **Age (Ref: 18 & Under)** |  |  |  |  |
| 19 | 0.71 (0.25 - 2.03) | 0.76 (0.30 - 1.93) | 0.65 (0.26 - 1.66) | 0.66 (0.26 - 1.67) |
| 20 & Older | 0.72 (0.31 - 1.67) | 0.78 (0.36 - 1.68) | 0.75 (0.39 - 1.46) | 0.74 (0.38 - 1.44) |
| Age Category: F (df, p) | 0.43 (2, p = 0.652) | 0.30 (2, p = 0.744) | 0.60 (2, p = 0.552) | 0.61 (2, p = 0.543) |
| **Gender (Ref: Female)** |  |  |  |  |
| Male | 0.82 (0.31 - 2.17) | 0.87 (0.35 - 2.13) | 1.00 (0.42 - 2.34) | 0.98 (0.42 - 2.31) |
| Gender Queer | 1.44 (0.60 - 3.44) | 0.73 (0.10 - 5.46) | 0.57 (0.07 - 4.44) | 0.55 (0.07 - 4.49) |
| Gender: F (df, p) | 0.45 (2, p = 0.635) | 0.08 (2, p = 0.927) | 0.15 (2, p = 0.863) | 0.16 (2, p = 0.849) |
| **Gender Modality (Ref: Cisgender)** |  |  |  |  |
| Transgender | 1.47 (0.69 - 3.13) | 1.60 (0.27 - 9.55) | 1.77 (0.26 - 11.94) | 1.87 (0.27 - 13.12) |
| **Sexual Orientation (Ref: Non-heterosexual)** |  |  |  |  |
| Non-heterosexual | 1.61 (0.84 - 3.10) | 1.43 (0.75 - 2.72) | 1.30 (0.66 - 2.53) | 1.30 (0.65 - 2.59) |
| **Enrolment Status (Ref: Full time)** |  |  |  |  |
| Part-time | 0.42 (0.00 - 643.33) | 0.93 (0.25 - 3.40) | 0.83 (0.25 - 2.76) | 0.84 (0.26 - 2.73) |
| **Parental Education (Ref: High School or Less)** |  |  |  |  |
| University or higher | 0.80 (0.42 - 1.52) | 0.79 (0.42 - 1.48) | 0.76 (0.39 - 1.48) | 0.75 (0.38 - 1.50) |
| **Ethnicity (Ref: Pākehā)** |  |  |  |  |
| Māori | 1.32 (0.54 - 3.23) | 1.32 (0.57 - 3.07) | 1.04 (0.47 - 2.32) | 1.06 (0.48 - 2.31) |
| Pacific Peoples | 1.84 (0.50 - 6.71) | 1.96 (0.52 - 7.39) | 1.76 (0.56 - 5.48) | 1.83 (0.59 - 5.71) |
| Asian | 0.82 (0.29 - 2.36) | 0.92 (0.33 - 2.57) | 1.33 (0.47 - 3.75) | 1.37 (0.47 - 3.97) |
| MELAA | 0.14 (0.00 - 2913.43) | 0.29 (0.00 - 527.50) | 0.31 (0.00 - 961.70) | 0.33 (0.00 - 960.46) |
| Ethnicity: F (df, p) | 0.17 (4, p = 0.955) | 0.15 (4, p = 0.964) | 0.14 (4, p = 0.968) | 0.14 (4, p = 0.966) |
| **Course (Ref: Arts & Sciences)** |  |  |  |  |
| Medical Sciences | 1.00 (0.36 - 2.78) | 0.95 (0.38 - 2.37) | 0.83 (0.31 - 2.19) | 0.83 (0.31 - 2.17) |
| Commerce | 0.86 (0.38 - 1.97) | 0.82 (0.38 - 1.80) | 0.83 (0.38 - 1.79) | 0.85 (0.39 - 1.85) |
| Laws | 1.29 (0.50 - 3.36) | 1.42 (0.55 - 3.69) | 1.41 (0.54 - 3.72) | 1.44 (0.56 - 3.70) |
| Course: F (df, p) | 0.16 (3, p = 0.921) | 0.34 (3, p = 0.798) | 0.36 (3, p = 0.780) | 0.36 (3, p = 0.779) |
| **Experiences of co-occuring Common Mental Disorders** |  |  |  |  |
| MDD | 1.04 (0.51 - 2.12) | - | 1.77 (0.64 - 4.87) | 1.95 (0.63 - 6.06) |
| BP | 1.93 (0.83 - 4.48) | - | 2.48 (0.79 - 7.79) | 2.73 (0.76 - 9.85) |
| GAD | 1.91 (0.93 - 3.95) | - | 1.19 (0.61 - 2.32) | 1.20 (0.61 - 2.37) |
| PD | 2.04 (0.92 - 4.54) | - | 1.52 (0.73 - 3.14) | 1.51 (0.73 - 3.15) |
| PTSD | **2.81 (1.04 - 7.57)*** | - | 1.80 (0.74 - 4.38) | 1.87 (0.72 - 4.86) |
| DUD | 1.72 (0.88 - 3.38) | - | 1.24 (0.62 - 2.46) | 1.13 (0.58 - 2.20) |
| AUD | 1.66 (0.81 - 3.40) | - | 1.22 (0.57 - 2.59) | 0.82 (0.17 - 4.03) |
| ADHD | 1.69 (0.89 - 3.21) | - | 1.05 (0.52 - 2.14) | 0.61 (0.07 - 5.25) |
| **Number of co-occuring Common Mental Disorders experienced** |  |  |  |  |
| 1 Other Disorder | 1.37 (0.34 - 5.41) | - | - | 1.84 (0.00 - 50391.69) |
| 2 Other Disorders | 2.76 (0.80 - 9.50) | - | - | 3.26 (0.00 - 83002.22) |
| 3 or more Other Disorders | 2.71 (0.61 - 12.02) | - | - | 5.58 (0.00 - 190282.84) |
| Number of Disorders experienced: F (df, p) | 1.79 (3, p = 0.149) | - | - | 0.03 (3, p = 0.992) |
| Abbreviations: RR, Risk Ratio, 95% CI, 95% Confidence Interval  Risk ratios and results are from multivariable Poisson distribution and robust standard errors. Data was multiply imputed, m = 20.  All univariable models controlled for year of survey and incentivisation  All multivariable models controlled for all variables included in the model, and for year of survey and incentivisation  Regression models were built hierarchically with socio-demographic variables entered in block 1, co-occurring mental disorders added in block 2, and number of co-occurring mental disorders added in block 3.  Co-occuring mental disorders are experienced in the past 12 months  * denotes significance difference, p < .05. | | | | |

Supplementary Table D: Year specific prevalence estimates of Lifetime and Past 12 Month Suicidal Thoughts and Behaviours

|  | Lifetime Outcomes (%, SE) | | | | Past 12 Month Outcomes (%, SE) | | | |
| --- | --- | --- | --- | --- | --- | --- | --- | --- |
|  | 2021 | 2022 | 2023 | F (df, p) | 2021 | 2022 | 2023 | F (df, p) |
| **Suicidal Thoughts or Behaviours** |  |  |  |  |  |  |  |  |
| Suicidal Ideation | 50.7 (1.9) | 54.9 (1.8) | 55.6 (1.6) | 2.49 (2, p = 0.083) | 35.1 (1.9) | 37.2 (1.9) | 38.1 (1.7) | 0.86 (2, p = 0.424) |
| Suicide Plan | 27.2 (1.8) | 29.4 (1.9) | 31.8 (1.6) | 1.63 (2, p = 0.196) | 17.3 (1.6) | 18.1 (1.5) | 19.1 (1.4) | 0.4 (2, p = 0.672) |
| Suicide Attempt | 9.2 (1.2) | 10.0 (1.2) | 11.0 (1.0) | 0.56 (2, p = 0.570) | 2.5 (0.6) | 2.6 (0.6) | 3.2 (0.7) | 0.27 (2, p = 0.766) |
| **Transition Outcomes given Ideation** |  |  |  |  |  |  |  |  |
| Suicidal Ideation Only | 43.6 (3.0) | 44.3 (2.9) | 40.4 (2.3) | 0.44 (2, p = 0.641) | 50.9 (3.7) | 51.0 (3.4) | 49.8 (3.0) | 0.01 (2, p = 0.987) |
| Plan if Ideation | 53.5 (3.0) | 53.6 (2.9) | 57.3 (2.3) | 0.54 (2, p = 0.585) | 49.0 (3.6) | 48.7 (3.4) | 49.9 (3.1) | 0.01 (2, p = 0.992) |
| Attempt if Ideation | 18.1 (2.4) | 18.3 (2.2) | 19.7 (1.8) | 0.21 (2, p = 0.808) | 7.0 (1.7) | 6.9 (1.7) | 8.3 (1.8) | 0.19 (2, p = 0.830) |
| **Transition Outcomes given Plan** |  |  |  |  |  |  |  |  |
| Plan without Attempt | 71.5 (3.8) | 69.9 (3.5) | 69.5 (3.0) | 0.09 (2, p = 0.912) | 86.0 (3.4) | 86.3 (3.3) | 84.0 (3.3) | 0.13 (2, p = 0.874) |
| Attempt if Plan | 28.5 (3.8) | 30.1 (3.5) | 30.5 (3.0) | 0.09 (2, p = 0.910) | 14.0 (3.4) | 13.7 (3.3) | 16.0 (3.3) | 0.14 (2, p = 0.869) |
| Abbreviations: SE, standard error, df, degrees of freedom  Prevalence estimates are weighted using post-stratification weights  F tests represents Wald-type F, showing whether the prevalence of the outcome differs across years | | | | | | | | |

Supplementary Table E: The associations of demographics variables and common mental disorders with lifetime suicide-related outcomes and transition along the suicidal trajectory, stratified by year, RR (95% CI)

|  | Suicide Ideation | | | Suicide Plan | | | Suicidal Ideation Only | | |
| --- | --- | --- | --- | --- | --- | --- | --- | --- | --- |
|  | 2021 (RR, 95% CI) | 2023 (RR, 95% CI) | F (df, p) | 2021 (RR, 95% CI) | 2023 (RR, 95% CI) | F (df, p) | 2021 (RR, 95% CI) | 2023 (RR, 95% CI) | F (df, p) |
| **Age (Ref: 18 & Under)** |  |  |  |  |  |  |  |  |  |
| 19 | 0.99 (0.80 - 1.22) | 1.05 (0.84 - 1.31) | 0.15 (2, 0.858) | 1.06 (0.71 - 1.59) | 1.13 (0.76 - 1.67) | 0.18 (2, 0.839) | 0.95 (0.62 - 1.45) | 0.95 (0.62 - 1.46) | 0.04 (2, 0.960) |
| 20 & Older | 1.01 (0.82 - 1.24) | 0.77 (0.63 - 0.94) | 4.22 (2, 0.015) | 1.15 (0.76 - 1.74) | 0.75 (0.50 - 1.12) | 2.23 (2, 0.108) | 0.79 (0.49 - 1.28) | 0.99 (0.63 - 1.57) | 0.55 (2, 0.578) |
| **Gender (Ref: Female)** |  |  |  |  |  |  |  |  |  |
| Male | 0.96 (0.77 - 1.20) | 0.92 (0.76 - 1.12) | 0.32 (2, 0.729) | 0.91 (0.57 - 1.43) | 0.95 (0.64 - 1.42) | 0.09 (2, 0.911) | 0.96 (0.64 - 1.45) | 0.95 (0.66 - 1.37) | 0.04 (2, 0.964) |
| Gender Queer | 1.61 (1.02 - 2.55) | 1.23 (0.81 - 1.89) | 2.06 (2, 0.128) | 1.20 (0.48 - 2.98) | 1.35 (0.55 - 3.29) | 0.21 (2, 0.807) | 87655.16 (1.19 - 6460439386.97) | 0.45 (0.00 - 744.88) | 4.06 (2, 0.025) |
| **Gender Modality (Ref: Cisgender)** | |  |  |  |  |  |  |  |  |
| Transgender | 0.69 (0.44 - 1.07) | 0.71 (0.48 - 1.05) | 1.90 (2, 0.150) | 0.82 (0.34 - 1.95) | 0.60 (0.27 - 1.35) | 0.72 (2, 0.488) | 0.00 (0.00 - 0.00) | 3.08 (0.00 - 5334.54) | 11.25 (2, 0.000) |
| **Sexual Orientation (Ref: Non-heterosexual)** | |  |  |  |  |  |  |  |  |
| Non-heterosexual | 0.84 (0.71 - 1.00) | 0.97 (0.83 - 1.13) | 2.08 (2, 0.126) | 0.81 (0.58 - 1.13) | 1.01 (0.72 - 1.41) | 1.10 (2, 0.334) | 0.97 (0.66 - 1.42) | 0.87 (0.57 - 1.31) | 0.29 (2, 0.751) |
| **Enrolment Status (Ref: Full time)** | |  |  |  |  |  |  |  |  |
| Part-time | 0.72 (0.48 - 1.09) | 0.96 (0.69 - 1.33) | 1.43 (2, 0.239) | 0.74 (0.34 - 1.63) | 0.94 (0.51 - 1.72) | 0.31 (2, 0.731) | 1.06 (0.48 - 2.35) | 1.22 (0.64 - 2.32) | 0.19 (2, 0.829) |
| **Parental Education (Ref: High School or Less)** | |  |  |  |  |  |  |  |  |
| University or higher | 0.90 (0.75 - 1.08) | 0.98 (0.84 - 1.16) | 0.72 (2, 0.488) | 0.83 (0.57 - 1.20) | 0.90 (0.66 - 1.22) | 0.55 (2, 0.577) | 1.11 (0.76 - 1.60) | 1.14 (0.81 - 1.59) | 0.31 (2, 0.736) |
| **Ethnicity (Ref: Pākehā)** |  |  |  |  |  |  |  |  |  |
| Māori | 1.28 (1.04 - 1.58) | 1.19 (0.98 - 1.44) | 2.85 (2, 0.058) | 1.17 (0.78 - 1.77) | 1.15 (0.79 - 1.66) | 0.33 (2, 0.720) | 1.05 (0.66 - 1.68) | 0.94 (0.58 - 1.52) | 0.13 (2, 0.878) |
| Pacific Peoples | 1.10 (0.79 - 1.54) | 1.04 (0.75 - 1.44) | 0.16 (2, 0.853) | 0.84 (0.40 - 1.75) | 1.04 (0.58 - 1.85) | 0.18 (2, 0.835) | 1.08 (0.52 - 2.22) | 1.01 (0.51 - 1.98) | 0.03 (2, 0.970) |
| Asian | 1.17 (0.88 - 1.56) | 1.00 (0.79 - 1.27) | 0.72 (2, 0.484) | 1.28 (0.75 - 2.18) | 1.22 (0.80 - 1.88) | 0.51 (2, 0.600) | 0.83 (0.54 - 1.29) | 0.75 (0.48 - 1.16) | 0.92 (2, 0.400) |
| MELAA | 1.17 (0.77 - 1.78) | 0.96 (0.63 - 1.47) | 0.51 (2, 0.602) | 1.21 (0.54 - 2.73) | 0.85 (0.38 - 1.90) | 0.53 (2, 0.591) | 0.63 (0.14 - 2.83) | 1.01 (0.39 - 2.60) | 0.23 (2, 0.795) |
| **Course (Ref: Arts & Sciences)** |  |  |  |  |  |  |  |  |  |
| Medical Sciences | 0.96 (0.70 - 1.30) | 0.89 (0.68 - 1.15) | 0.41 (2, 0.664) | 0.79 (0.40 - 1.54) | 1.24 (0.73 - 2.09) | 1.02 (2, 0.362) | 1.19 (0.78 - 1.82) | 0.75 (0.46 - 1.23) | 1.47 (2, 0.231) |
| Commerce | 1.01 (0.83 - 1.23) | 0.93 (0.78 - 1.12) | 0.41 (2, 0.665) | 0.96 (0.64 - 1.43) | 1.01 (0.70 - 1.46) | 0.04 (2, 0.959) | 1.01 (0.62 - 1.67) | 0.91 (0.57 - 1.45) | 0.13 (2, 0.882) |
| Laws | 0.99 (0.72 - 1.37) | 0.99 (0.75 - 1.32) | 0.00 (2, 0.998) | 0.98 (0.48 - 2.02) | 1.25 (0.70 - 2.21) | 0.39 (2, 0.681) | 0.92 (0.34 - 2.48) | 0.66 (0.28 - 1.57) | 0.45 (2, 0.636) |
| **Experiences of co-occuring Common Mental Disorders** | | |  |  |  |  |  |  |  |
| MDD | 1.18 (0.96 - 1.43) | 1.13 (0.94 - 1.35) | 1.39 (2, 0.248) | 1.29 (0.84 - 1.99) | 1.24 (0.79 - 1.95) | 0.69 (2, 0.501) | 0.83 (0.51 - 1.37) | 0.77 (0.45 - 1.33) | 0.52 (2, 0.593) |
| BP | 0.94 (0.69 - 1.28) | 0.98 (0.78 - 1.25) | 0.07 (2, 0.934) | 1.13 (0.61 - 2.09) | 0.99 (0.54 - 1.80) | 0.10 (2, 0.907) | 0.60 (0.06 - 5.94) | 1.24 (0.26 - 6.04) | 0.22 (2, 0.806) |
| GAD | 0.85 (0.71 - 1.02) | 0.78 (0.66 - 0.92) | 4.49 (2, 0.011) | 0.81 (0.56 - 1.18) | 0.71 (0.51 - 0.98) | 2.21 (2, 0.110) | 1.06 (0.61 - 1.81) | 1.16 (0.71 - 1.92) | 0.19 (2, 0.827) |
| PTSD | 0.98 (0.76 - 1.25) | 1.12 (0.87 - 1.44) | 0.66 (2, 0.517) | 1.08 (0.62 - 1.87) | 1.10 (0.69 - 1.76) | 0.09 (2, 0.918) | 0.90 (0.51 - 1.60) | 0.97 (0.57 - 1.68) | 0.07 (2, 0.928) |
| PD | 1.12 (0.92 - 1.37) | 1.10 (0.93 - 1.31) | 0.89 (2, 0.412) | 1.06 (0.70 - 1.62) | 1.15 (0.80 - 1.65) | 0.27 (2, 0.763) | 1.11 (0.48 - 2.58) | 0.86 (0.40 - 1.85) | 0.21 (2, 0.807) |
| DUD | 0.94 (0.72 - 1.23) | 1.13 (0.90 - 1.43) | 1.11 (2, 0.331) | 0.57 (0.34 - 0.95) | 1.03 (0.67 - 1.58) | 3.20 (2, 0.042) | 2.04 (1.09 - 3.79) | 1.13 (0.54 - 2.37) | 2.67 (2, 0.071) |
| AUD | 0.76 (0.52 - 1.10) | 1.01 (0.69 - 1.46) | 1.51 (2, 0.221) | 0.92 (0.45 - 1.88) | 1.31 (0.64 - 2.68) | 0.58 (2, 0.562) | 0.72 (0.36 - 1.44) | 0.74 (0.35 - 1.58) | 0.48 (2, 0.617) |
| ADHD | 0.75 (0.45 - 1.23) | 1.18 (0.76 - 1.81) | 1.76 (2, 0.173) | 0.77 (0.31 - 1.93) | 1.43 (0.64 - 3.20) | 1.13 (2, 0.325) | 1.09 (0.45 - 2.65) | 0.96 (0.40 - 2.32) | 0.04 (2, 0.959) |
| **Number of co-occuring Common Mental Disorders experienced** | | |  |  |  |  |  |  |  |
| 1 Other Disorder | 0.94 (0.58 - 1.53) | 0.79 (0.51 - 1.22) | 0.65 (2, 0.521) | 1.22 (0.43 - 3.42) | 0.87 (0.36 - 2.12) | 0.25 (2, 0.777) | 0.91 (0.43 - 1.92) | 0.95 (0.46 - 1.97) | 0.03 (2, 0.971) |
| 2 Other Disorders | 1.16 (0.60 - 2.24) | 0.68 (0.36 - 1.30) | 1.44 (2, 0.238) | 1.25 (0.30 - 5.27) | 0.56 (0.16 - 1.97) | 0.86 (2, 0.424) | 1.10 (0.29 - 4.20) | 1.32 (0.34 - 5.11) | 0.10 (2, 0.908) |
| 3 or more Other Disorders | 1.73 (0.64 - 4.73) | 0.60 (0.22 - 1.62) | 2.32 (2, 0.099) | 2.25 (0.29 - 17.66) | 0.46 (0.07 - 2.96) | 1.42 (2, 0.244) | 0.86 (0.10 - 7.75) | 1.26 (0.16 - 10.17) | 0.07 (2, 0.935) |
| Abbreviations: RR, Risk Ratio, 95% CI, 95% Confidence Interval  Risk ratios and results are from multivariable Poisson distribution with robust standard errors, testing the interaction with all predictors, adjusting for incentive. The predictors presented are all interacting with year, e.g., YEAR X predictor.  Data was multiply imputed, m = 20.  * denotes significance difference, p < .05. | | | | | | | | | |

Supplementary Table E CTNE: The associations of demographics variables and common mental disorders with lifetime suicide-related outcomes and transition along the suicidal trajectory, stratified by year, RR (95% CI)

|  | Plan if Ideation | | | Plan without Attempt | | |
| --- | --- | --- | --- | --- | --- | --- |
|  | 2021 (RR, 95% CI) | 2023 (RR, 95% CI) | F (df, p) | 2021 (RR, 95% CI) | 2023 (RR, 95% CI) | F (df, p) |
| **Age (Ref: 18 & Under)** |  |  |  |  |  |  |
| 19 | 1.07 (0.75 - 1.55) | 1.08 (0.76 - 1.54) | 0.11 (2, 0.895) | 0.97 (0.69 - 1.37) | 0.82 (0.58 - 1.15) | 0.71 (2, 0.492) |
| 20 & Older | 1.18 (0.81 - 1.72) | 0.97 (0.68 - 1.38) | 0.63 (2, 0.535) | 0.84 (0.55 - 1.27) | 0.91 (0.62 - 1.35) | 0.37 (2, 0.691) |
| **Gender (Ref: Female)** |  |  |  |  |  |  |
| Male | 0.93 (0.61 - 1.41) | 1.03 (0.73 - 1.48) | 0.16 (2, 0.856) | 1.08 (0.76 - 1.54) | 1.06 (0.78 - 1.43) | 0.13 (2, 0.882) |
| Gender Queer | 0.84 (0.38 - 1.87) | 1.19 (0.53 - 2.69) | 0.36 (2, 0.698) | 6.86 (0.33 - 140.71) | 0.97 (0.38 - 2.50) | 1.00 (2, 0.371) |
| **Gender Modality (Ref: Cisgender)** |  |  |  |  |  |  |
| Transgender | 1.07 (0.51 - 2.26) | 0.78 (0.37 - 1.64) | 0.40 (2, 0.673) | 0.15 (0.01 - 2.85) | 1.09 (0.48 - 2.50) | 1.10 (2, 0.335) |
| **Sexual Orientation (Ref: Non-heterosexual)** |  |  |  |  |  |  |
| Non**-**heterosexual | 0.94 (0.70 - 1.28) | 1.05 (0.78 - 1.42) | 0.26 (2, 0.769) | 0.86 (0.61 - 1.22) | 1.00 (0.76 - 1.31) | 0.56 (2, 0.574) |
| **Enrolment Status (Ref: Full time)** |  |  |  |  |  |  |
| Part**-**time | 0.97 (0.47 - 2.00) | 0.93 (0.52 - 1.66) | 0.03 (2, 0.974) | 1.24 (0.61 - 2.52) | 0.96 (0.50 - 1.85) | 0.36 (2, 0.699) |
| **Parental Education (Ref: High School or Less)** |  |  |  |  |  |  |
| University or higher | 0.89 (0.65 - 1.24) | 0.91 (0.70 - 1.18) | 0.31 (2, 0.732) | 1.00 (0.76 - 1.33) | 1.00 (0.77 - 1.30) | 0.00 (2, 0.999) |
| **Ethnicity (Ref: Pākehā)** |  |  |  |  |  |  |
| Māori | 0.94 (0.66 - 1.34) | 0.99 (0.72 - 1.37) | 0.07 (2, 0.936) | 0.90 (0.63 - 1.27) | 0.89 (0.60 - 1.31) | 0.25 (2, 0.780) |
| Pacific Peoples | 0.75 (0.38 - 1.49) | 0.95 (0.57 - 1.58) | 0.39 (2, 0.679) | 1.53 (0.81 - 2.90) | 1.33 (0.67 - 2.63) | 0.83 (2, 0.438) |
| Asian | 1.18 (0.73 - 1.90) | 1.25 (0.86 - 1.84) | 0.62 (2, 0.538) | 1.03 (0.66 - 1.61) | 1.07 (0.70 - 1.62) | 0.05 (2, 0.947) |
| MELAA | 1.06 (0.53 - 2.11) | 0.92 (0.45 - 1.89) | 0.10 (2, 0.904) | 1.23 (0.68 - 2.23) | 1.16 (0.58 - 2.32) | 0.22 (2, 0.803) |
| **Course (Ref: Arts & Sciences)** |  |  |  |  |  |  |
| Medical Sciences | 0.79 (0.43 - 1.45) | 1.32 (0.82 - 2.11) | 1.79 (2, 0.168) | 1.06 (0.64 - 1.77) | 1.20 (0.76 - 1.89) | 0.36 (2, 0.700) |
| Commerce | 0.96 (0.67 - 1.39) | 1.08 (0.78 - 1.50) | 0.27 (2, 0.765) | 0.92 (0.65 - 1.32) | 0.96 (0.68 - 1.37) | 0.10 (2, 0.902) |
| Laws | 1.01 (0.52 - 1.94) | 1.29 (0.78 - 2.16) | 0.59 (2, 0.554) | 0.82 (0.29 - 2.37) | 1.20 (0.64 - 2.27) | 0.42 (2, 0.657) |
| **Experiences of co-occuring Common Mental Disorders** |  |  |  |  |  |  |
| MDD | 1.10 (0.75 - 1.59) | 1.10 (0.74 - 1.65) | 0.15 (2, 0.859) | 1.09 (0.76 - 1.57) | 0.90 (0.62 - 1.29) | 0.60 (2, 0.547) |
| BP | 1.16 (0.69 - 1.95) | 1.01 (0.61 - 1.67) | 0.17 (2, 0.841) | 0.94 (0.33 - 2.69) | 1.02 (0.41 - 2.53) | 0.01 (2, 0.990) |
| GAD | 0.95 (0.69 - 1.30) | 0.89 (0.66 - 1.20) | 0.29 (2, 0.749) | 0.92 (0.62 - 1.38) | 0.96 (0.65 - 1.44) | 0.09 (2, 0.918) |
| PTSD | 1.09 (0.68 - 1.73) | 0.97 (0.65 - 1.46) | 0.13 (2, 0.879) | 1.05 (0.73 - 1.51) | 0.95 (0.62 - 1.44) | 0.15 (2, 0.865) |
| PD | 0.95 (0.62 - 1.45) | 1.05 (0.75 - 1.47) | 0.16 (2, 0.854) | 0.93 (0.60 - 1.44) | 1.05 (0.72 - 1.54) | 0.18 (2, 0.838) |
| DUD | 0.59 (0.38 - 0.93) | 0.89 (0.62 - 1.29) | 2.78 (2, 0.064) | 1.04 (0.58 - 1.87) | 1.05 (0.66 - 1.67) | 0.02 (2, 0.976) |
| AUD | 1.22 (0.61 - 2.43) | 1.33 (0.69 - 2.56) | 0.41 (2, 0.665) | 1.12 (0.63 - 2.00) | 0.87 (0.45 - 1.68) | 0.38 (2, 0.686) |
| ADHD | 1.01 (0.44 - 2.34) | 1.19 (0.56 - 2.52) | 0.15 (2, 0.857) | 1.08 (0.55 - 2.12) | 0.81 (0.39 - 1.67) | 0.37 (2, 0.691) |
| **Number of co-occuring Common Mental Disorders experienced** |  |  |  |  |  |  |
| 1 Other Disorder | 1.31 (0.54 - 3.23) | 1.13 (0.55 - 2.36) | 0.18 (2, 0.833) | 0.92 (0.53 - 1.60) | 1.02 (0.57 - 1.81) | 0.07 (2, 0.929) |
| 2 Other Disorders | 1.14 (0.31 - 4.20) | 0.83 (0.28 - 2.52) | 0.15 (2, 0.865) | 0.82 (0.32 - 2.09) | 1.21 (0.44 - 3.36) | 0.35 (2, 0.704) |
| 3 or more Other Disorders | 1.41 (0.21 - 9.36) | 0.78 (0.14 - 4.22) | 0.23 (2, 0.798) | 0.86 (0.16 - 4.65) | 1.38 (0.24 - 7.92) | 0.18 (2, 0.835) |
| Abbreviations: RR, Risk Ratio, 95% CI, 95% Confidence Interval  Risk ratios and results are from multivariable Poisson distribution with robust standard errors, testing the interaction with all predictors, adjusting for incentive. The predictors presented are all interacting with year, e.g., YEAR X predictor.  Data was multiply imputed, m = 20.  * denotes significance difference, p < .05. | | | | | | |

Supplementary Table F: The associations of demographics variables and common mental disorders with past 12 month suicide-related outcomes and transition along the suicidal trajectory, stratified by year, RR (95% CI)

|  | Suicidal Ideation | | | Suicide Plan | | | Suicide Attempt | | |
| --- | --- | --- | --- | --- | --- | --- | --- | --- | --- |
|  | 2021 (RR, 95% CI) | 2023 (RR, 95% CI) | F (df, p) | 2021 (RR, 95% CI) | 2023 (RR, 95% CI) | F (df, p) | 2021 (RR, 95% CI) | 2023 (RR, 95% CI) | F (df, p) |
| **Age (Ref: 18 & Under)** |  |  |  |  |  |  |  |  |  |
| 19 | 1.00 (0.74 - 1.36) | 1.11 (0.79 - 1.55) | 0.25 (2, 0.782) | 1.17 (0.69 - 2.00) | 1.33 (0.81 - 2.17) | 0.55 (2, 0.578) | 0.86 (0.57 - 1.29) | 0.78 (0.51 - 1.19) | 0.60 (2, 0.550) |
| 20 & Older | 1.01 (0.73 - 1.42) | 0.71 (0.51 - 0.99) | 2.58 (2, 0.077) | 1.15 (0.67 - 1.98) | 0.63 (0.36 - 1.10) | 2.31 (2, 0.100) | 0.81 (0.52 - 1.28) | 1.16 (0.75 - 1.78) | 1.12 (2, 0.326) |
| **Gender (Ref: Female)** |  |  |  |  |  |  |  |  |  |
| Male | 0.87 (0.61 - 1.24) | 0.90 (0.64 - 1.26) | 0.35 (2, 0.704) | 0.97 (0.52 - 1.82) | 1.21 (0.71 - 2.08) | 0.39 (2, 0.679) | 0.89 (0.57 - 1.37) | 0.74 (0.50 - 1.10) | 1.15 (2, 0.317) |
| Gender Queer | 1.52 (0.69 - 3.34) | 1.01 (0.45 - 2.27) | 0.67 (2, 0.511) | 1.21 (0.33 - 4.44) | 1.19 (0.32 - 4.48) | 0.05 (2, 0.949) | 383415.56 (21731.31 - 6764777.32) | 0.74 (0.07 - 7.97) | 58.89 (2, 0.000) |
| **Gender Modality (Ref: Cisgender)** |  |  |  |  |  |  |  |  |  |
| Transgender | 0.70 (0.34 - 1.43) | 0.87 (0.41 - 1.87) | 0.46 (2, 0.632) | 0.79 (0.27 - 2.31) | 0.77 (0.24 - 2.51) | 0.13 (2, 0.874) | 0.00 (0.00 - 0.00) | 1.09 (0.12 - 9.53) | 80.98 (2, 0.000) |
| **Sexual Orientation (Ref: Non-heterosexual)** |  |  |  |  |  |  |  |  |  |
| Non**-**heterosexual | 0.80 (0.61 - 1.05) | 0.98 (0.76 - 1.28) | 1.63 (2, 0.197) | 0.83 (0.50 - 1.38) | 1.09 (0.66 - 1.81) | 0.66 (2, 0.519) | 1.01 (0.64 - 1.60) | 0.93 (0.62 - 1.41) | 0.10 (2, 0.902) |
| **Enrolment Status (Ref: Full time)** |  |  |  |  |  |  |  |  |  |
| Part**-**time | 0.54 (0.27 - 1.07) | 0.89 (0.50 - 1.58) | 1.86 (2, 0.156) | 0.70 (0.27 - 1.82) | 1.06 (0.43 - 2.61) | 0.45 (2, 0.637) | 0.86 (0.38 - 1.96) | 0.82 (0.38 - 1.76) | 0.13 (2, 0.875) |
| **Parental Education (Ref: High School or Less)** |  |  |  |  |  |  |  |  |  |
| University or higher | 0.92 (0.70 - 1.21) | 1.10 (0.86 - 1.40) | 0.93 (2, 0.394) | 0.85 (0.54 - 1.35) | 0.99 (0.63 - 1.54) | 0.26 (2, 0.771) | 1.09 (0.77 - 1.56) | 1.12 (0.79 - 1.60) | 0.23 (2, 0.792) |
| **Ethnicity (Ref: Pākehā)** |  |  |  |  |  |  |  |  |  |
| Māori | 1.27 (0.92 - 1.76) | 1.32 (0.98 - 1.78) | 1.87 (2, 0.154) | 1.01 (0.57 - 1.79) | 1.14 (0.70 - 1.84) | 0.17 (2, 0.844) | 1.26 (0.75 - 2.11) | 1.07 (0.66 - 1.73) | 0.42 (2, 0.661) |
| Pacific Peoples | 1.17 (0.71 - 1.92) | 1.14 (0.73 - 1.79) | 0.23 (2, 0.793) | 0.80 (0.29 - 2.23) | 1.18 (0.53 - 2.61) | 0.30 (2, 0.740) | 1.16 (0.57 - 2.35) | 0.92 (0.48 - 1.75) | 0.27 (2, 0.767) |
| Asian | 1.10 (0.72 - 1.69) | 1.00 (0.69 - 1.43) | 0.14 (2, 0.872) | 0.80 (0.38 - 1.66) | 1.04 (0.58 - 1.84) | 0.31 (2, 0.733) | 1.16 (0.70 - 1.93) | 0.88 (0.56 - 1.38) | 0.61 (2, 0.543) |
| MELAA | 2.14 (1.08 - 4.21) | 1.52 (0.79 - 2.94) | 2.42 (2, 0.089) | 1.90 (0.53 - 6.84) | 1.00 (0.29 - 3.45) | 0.87 (2, 0.422) | 0.83 (0.12 - 5.66) | 1.33 (0.31 - 5.80) | 0.23 (2, 0.792) |
| **Course (Ref: Arts & Sciences)** |  |  |  |  |  |  |  |  |  |
| Medical Sciences | 0.91 (0.58 - 1.42) | 0.96 (0.64 - 1.43) | 0.09 (2, 0.915) | 0.59 (0.21 - 1.68) | 1.32 (0.67 - 2.59) | 1.27 (2, 0.281) | 1.24 (0.73 - 2.11) | 0.75 (0.42 - 1.34) | 1.31 (2, 0.271) |
| Commerce | 1.07 (0.78 - 1.49) | 0.94 (0.70 - 1.26) | 0.43 (2, 0.651) | 0.91 (0.52 - 1.60) | 0.93 (0.51 - 1.69) | 0.05 (2, 0.947) | 1.13 (0.62 - 2.06) | 1.02 (0.57 - 1.83) | 0.11 (2, 0.894) |
| Laws | 1.32 (0.78 - 2.23) | 1.26 (0.79 - 2.02) | 0.69 (2, 0.504) | 1.28 (0.47 - 3.47) | 1.55 (0.71 - 3.40) | 0.57 (2, 0.566) | 0.84 (0.31 - 2.29) | 0.68 (0.31 - 1.49) | 0.41 (2, 0.664) |
| **Experiences of co-occuring Common Mental Disorders** |  |  |  |  |  |  |  |  |  |
| MDD | 1.17 (0.82 - 1.66) | 1.15 (0.82 - 1.61) | 0.46 (2, 0.629) | 1.48 (0.71 - 3.11) | 1.19 (0.60 - 2.37) | 0.53 (2, 0.590) | 0.82 (0.46 - 1.45) | 0.90 (0.49 - 1.64) | 0.23 (2, 0.794) |
| BP | 1.14 (0.69 - 1.88) | 1.09 (0.70 - 1.71) | 0.15 (2, 0.863) | 1.32 (0.50 - 3.48) | 0.87 (0.37 - 2.04) | 0.38 (2, 0.686) | 0.79 (0.16 - 3.96) | 1.52 (0.42 - 5.50) | 0.47 (2, 0.624) |
| GAD | 0.73 (0.54 - 0.97) | 0.72 (0.54 - 0.95) | 3.35 (2, 0.036) | 0.71 (0.42 - 1.21) | 0.68 (0.43 - 1.09) | 1.45 (2, 0.237) | 1.00 (0.56 - 1.76) | 1.12 (0.65 - 1.90) | 0.12 (2, 0.886) |
| PTSD | 1.09 (0.74 - 1.61) | 1.01 (0.74 - 1.38) | 0.15 (2, 0.859) | 1.17 (0.57 - 2.38) | 0.85 (0.49 - 1.46) | 0.54 (2, 0.585) | 0.94 (0.49 - 1.79) | 1.11 (0.64 - 1.94) | 0.17 (2, 0.842) |
| PD | 1.09 (0.81 - 1.48) | 1.08 (0.81 - 1.43) | 0.21 (2, 0.812) | 1.22 (0.71 - 2.10) | 1.37 (0.85 - 2.23) | 0.80 (2, 0.452) | 0.88 (0.45 - 1.72) | 0.76 (0.43 - 1.35) | 0.39 (2, 0.677) |
| DUD | 0.84 (0.56 - 1.26) | 0.97 (0.68 - 1.40) | 0.42 (2, 0.657) | 0.72 (0.36 - 1.45) | 0.99 (0.57 - 1.75) | 0.54 (2, 0.583) | 1.25 (0.61 - 2.57) | 1.03 (0.56 - 1.90) | 0.22 (2, 0.805) |
| AUD | 0.84 (0.49 - 1.46) | 0.91 (0.54 - 1.53) | 0.17 (2, 0.844) | 1.04 (0.37 - 2.93) | 1.26 (0.49 - 3.28) | 0.15 (2, 0.861) | 0.72 (0.34 - 1.54) | 0.83 (0.39 - 1.76) | 0.35 (2, 0.705) |
| ADHD | 0.97 (0.47 - 2.01) | 0.98 (0.55 - 1.74) | 0.00 (2, 0.996) | 0.93 (0.25 - 3.50) | 1.30 (0.42 - 4.02) | 0.19 (2, 0.827) | 0.98 (0.41 - 2.35) | 1.01 (0.40 - 2.52) | 0.00 (2, 0.998) |
| **Number of co-occuring Common Mental Disorders experienced** |  |  |  |  |  |  |  |  |  |
| 1 Other Disorder | 0.82 (0.42 - 1.59) | 0.97 (0.52 - 1.81) | 0.20 (2, 0.819) | 0.62 (0.15 - 2.61) | 1.23 (0.35 - 4.26) | 0.55 (2, 0.577) | 1.30 (0.54 - 3.10) | 0.86 (0.38 - 1.95) | 0.50 (2, 0.606) |
| 2 Other Disorders | 0.97 (0.34 - 2.75) | 0.97 (0.40 - 2.38) | 0.00 (2, 0.997) | 0.58 (0.07 - 4.89) | 0.79 (0.12 - 5.40) | 0.13 (2, 0.875) | 1.64 (0.37 - 7.34) | 1.10 (0.24 - 4.93) | 0.24 (2, 0.785) |
| 3 or more Other Disorders | 1.21 (0.25 - 5.77) | 1.04 (0.27 - 4.05) | 0.03 (2, 0.967) | 0.90 (0.04 - 19.18) | 0.96 (0.06 - 14.58) | 0.00 (2, 0.998) | 1.25 (0.14 - 11.31) | 0.64 (0.06 - 6.87) | 0.18 (2, 0.833) |
| Abbreviations: RR, Risk Ratio, 95% CI, 95% Confidence IntervalRisk ratios and results are from multivariable Poisson distribution with robust standard errors, testing the interaction with all predictors, adjusting for incentive. The predictors presented are all interacting with year, e.g., YEAR X predictor.  Data was multiply imputed, m = 20.  * denotes significance difference, p < .05. F tests represents Wald-type F, showing whether the point estimate of the outcome differs across years | | | | | | | | | |

Supplementary Table F CTNE: The associations of demographics variables and common mental disorders with past 12 month suicide-related outcomes and transition along the suicidal trajectory, stratified by year, RR (95% CI)

|  | Suicidal Ideation Only | | | Suicide Plan without Attempt | | |
| --- | --- | --- | --- | --- | --- | --- |
|  | 2021 (RR, 95% CI) | 2023 (RR, 95% CI) | F (df, p) | 2021 (RR, 95% CI) | 2023 (RR, 95% CI) | F (df, p) |
| **Age (Ref: 18 & Under)** |  |  |  |  |  |  |
| 19 | 1.19 (0.74 - 1.91) | 1.26 (0.82 - 1.93) | 0.50 (2, 0.605) | 1.10 (0.81 - 1.50) | 1.05 (0.77 - 1.43) | 0.22 (2, 0.800) |
| 20 & Older | 1.19 (0.75 - 1.87) | 0.83 (0.50 - 1.37) | 1.01 (2, 0.367) | 0.90 (0.67 - 1.21) | 0.94 (0.69 - 1.27) | 0.28 (2, 0.756) |
| **Gender (Ref: Female)** |  |  |  |  |  |  |
| Male | 1.10 (0.62 - 1.98) | 1.41 (0.86 - 2.32) | 1.04 (2, 0.356) | 1.01 (0.75 - 1.38) | 0.95 (0.75 - 1.20) | 0.12 (2, 0.887) |
| Gender Queer | 0.96 (0.31 - 2.98) | 1.22 (0.42 - 3.56) | 0.11 (2, 0.894) | 1.06 (0.46 - 2.47) | 0.97 (0.42 - 2.25) | 0.02 (2, 0.981) |
| **Gender Modality (Ref: Cisgender)** |  |  |  |  |  |  |
| Transgender | 1.00 (0.41 - 2.46) | 0.91 (0.35 - 2.37) | 0.03 (2, 0.974) | 0.98 (0.44 - 2.15) | 0.94 (0.48 - 1.83) | 0.01 (2, 0.986) |
| **Sexual Orientation (Ref: Non-heterosexual)** |  |  |  |  |  |  |
| Non**-**heterosexual | 0.98 (0.63 - 1.51) | 1.10 (0.72 - 1.67) | 0.17 (2, 0.840) | 0.97 (0.77 - 1.23) | 0.98 (0.82 - 1.18) | 0.03 (2, 0.972) |
| **Enrolment Status (Ref: Full time)** |  |  |  |  |  |  |
| Part**-**time | 1.15 (0.49 - 2.69) | 1.09 (0.49 - 2.42) | 0.05 (2, 0.954) | 1.26 (0.74 - 2.16) | 0.91 (0.43 - 1.94) | 0.64 (2, 0.530) |
| **Parental Education (Ref: High School or Less)** |  |  |  |  |  |  |
| University or higher | 0.90 (0.61 - 1.33) | 0.88 (0.60 - 1.29) | 0.24 (2, 0.787) | 0.97 (0.77 - 1.22) | 0.99 (0.81 - 1.22) | 0.05 (2, 0.955) |
| **Ethnicity (Ref: Pākehā)** |  |  |  |  |  |  |
| Māori | 0.83 (0.51 - 1.35) | 0.88 (0.59 - 1.32) | 0.33 (2, 0.722) | 1.09 (0.81 - 1.47) | 0.97 (0.75 - 1.25) | 0.36 (2, 0.700) |
| Pacific Peoples | 0.77 (0.31 - 1.89) | 1.05 (0.52 - 2.12) | 0.26 (2, 0.773) | 1.29 (0.84 - 1.96) | 1.00 (0.58 - 1.72) | 1.09 (2, 0.338) |
| Asian | 0.83 (0.42 - 1.64) | 1.10 (0.67 - 1.80) | 0.41 (2, 0.666) | 0.96 (0.69 - 1.34) | 0.88 (0.68 - 1.14) | 0.44 (2, 0.647) |
| MELAA | 1.06 (0.34 - 3.37) | 0.78 (0.24 - 2.50) | 0.23 (2, 0.796) | 1.15 (0.52 - 2.57) | 0.98 (0.37 - 2.61) | 0.17 (2, 0.840) |
| **Course (Ref: Arts & Sciences)** |  |  |  |  |  |  |
| Medical Sciences | 0.64 (0.24 - 1.71) | 1.40 (0.74 - 2.64) | 1.53 (2, 0.218) | 0.99 (0.59 - 1.65) | 0.92 (0.67 - 1.26) | 0.14 (2, 0.869) |
| Commerce | 0.91 (0.55 - 1.52) | 1.00 (0.61 - 1.65) | 0.09 (2, 0.914) | 1.01 (0.81 - 1.27) | 0.97 (0.77 - 1.23) | 0.06 (2, 0.945) |
| Laws | 1.11 (0.44 - 2.80) | 1.35 (0.70 - 2.59) | 0.37 (2, 0.691) | 0.83 (0.33 - 2.10) | 1.09 (0.56 - 2.13) | 0.25 (2, 0.781) |
| **Experiences of co-occuring Common Mental Disorders** |  |  |  |  |  |  |
| MDD | 1.24 (0.66 - 2.33) | 1.06 (0.55 - 2.02) | 0.22 (2, 0.805) | 1.03 (0.78 - 1.35) | 1.16 (0.90 - 1.49) | 0.64 (2, 0.527) |
| BP | 1.16 (0.50 - 2.67) | 0.83 (0.39 - 1.78) | 0.33 (2, 0.716) | 1.02 (0.55 - 1.89) | 1.02 (0.54 - 1.93) | 0.00 (2, 0.998) |
| GAD | 0.97 (0.63 - 1.51) | 0.92 (0.60 - 1.42) | 0.07 (2, 0.929) | 1.06 (0.85 - 1.33) | 1.11 (0.85 - 1.43) | 0.35 (2, 0.704) |
| PTSD | 1.07 (0.60 - 1.88) | 0.87 (0.55 - 1.36) | 0.36 (2, 0.696) | 1.02 (0.80 - 1.29) | 0.97 (0.75 - 1.26) | 0.06 (2, 0.942) |
| PD | 1.13 (0.71 - 1.79) | 1.25 (0.86 - 1.82) | 0.59 (2, 0.557) | 0.99 (0.67 - 1.45) | 1.11 (0.79 - 1.56) | 0.26 (2, 0.774) |
| DUD | 0.79 (0.43 - 1.45) | 0.96 (0.60 - 1.53) | 0.31 (2, 0.733) | 1.15 (0.73 - 1.81) | 1.21 (0.82 - 1.80) | 0.50 (2, 0.607) |
| AUD | 1.44 (0.61 - 3.41) | 1.52 (0.65 - 3.55) | 0.57 (2, 0.567) | 1.26 (0.69 - 2.30) | 1.26 (0.82 - 1.93) | 0.58 (2, 0.561) |
| ADHD | 1.10 (0.38 - 3.19) | 1.37 (0.50 - 3.76) | 0.24 (2, 0.786) | 1.24 (0.70 - 2.19) | 1.25 (0.78 - 2.00) | 0.53 (2, 0.592) |
| **Number of co-occuring Common Mental Disorders experienced** |  |  |  |  |  |  |
| 1 Other Disorder | 0.74 (0.22 - 2.48) | 1.34 (0.44 - 4.03) | 0.54 (2, 0.585) | 0.96 (0.62 - 1.48) | 0.83 (0.57 - 1.22) | 0.41 (2, 0.664) |
| 2 Other Disorders | 0.56 (0.09 - 3.34) | 0.81 (0.14 - 4.72) | 0.20 (2, 0.817) | 0.73 (0.31 - 1.74) | 0.62 (0.32 - 1.20) | 0.86 (2, 0.424) |
| 3 or more Other Disorders | 0.61 (0.05 - 7.56) | 0.87 (0.07 - 10.75) | 0.07 (2, 0.933) | 0.54 (0.12 - 2.36) | 0.46 (0.15 - 1.38) | 0.90 (2, 0.410) |
| Abbreviations: RR, Risk Ratio, 95% CI, 95% Confidence Interval  Risk ratios and results are from multivariable Poisson distribution with robust standard errors, testing the interaction with all predictors, adjusting for incentive. The predictors presented are all interacting with year, e.g., YEAR X predictor.  Data was multiply imputed, m = 20.  * denotes significance difference, p < .05.  F tests represents Wald-type F, showing whether the point estimate of the outcome differs across years | | | | | | |
